# Supplementary material for: Mapping brain-wide monosynaptic inputs to single neurons with ROInet-seq
Source: Cell Rep Methods. 2026 Jul 7;6(9):101512. doi: 10.1016/j.crmeth.2026.101512 (PMC13615506; doi:10.1016/j.crmeth.2026.101512)
Supplement: Supplementary file 1 [file mmc1.pdf]

Cell Reports Methods, Volume *6*

Supplemental information

Mapping brain-wide monosynaptic inputs to single neurons with ROInet-seq

Zhige Lin, Osnat Ophir, Thorsten Trimbuch, Bettina Gann, Muhammad Tibi, Hermann Schmidt, Christian Rosenmund, Hannah Hochgerner, and Amit Zeisel

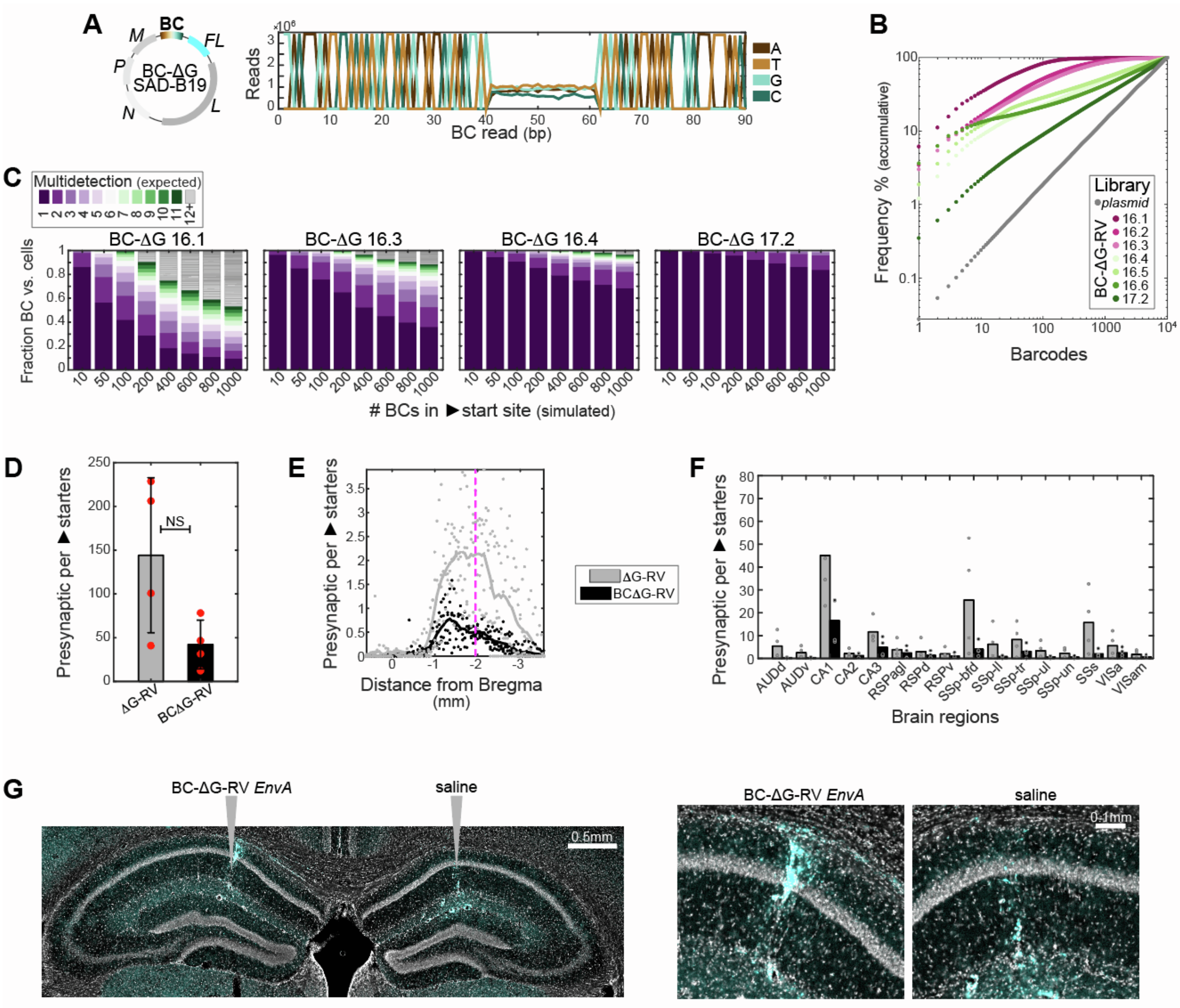

Figure S1. Characterization of BC-DG-RV libraries. Related to Figure 1.

(**A**) Sequencing verification of a uniformly random 21nt BC insert, shown as pseudo-Sanger bp frequency plot.

(**B**) Accumulative frequency of top 10k most frequent barcodes per RV-library, vs. the optimally uniform plasmid library.

(**C**) Expected detection of a unique BC in 1-12+ cells, given 8 scenarios based on 10-1000 starter cells, per library.

(**D**) Quantification of presynaptic-per-starter cells after infection to the CA1 (unilateral) with DG-RV (n=4; grey; 144.1 +/-88.5) and BC-DG-RV (n=4; black; 42.2+/-27.9).

(**E**) As (**D**), quantification of presynaptic-per-starter neurons resolved to the sections along the anterior-posterior axis. Pink line, injection coordinates.

(**F**) As (**D**), quantification of presynaptic-per-starter neurons, summed by brain region.

(**G**) *In vivo* EnvA pseudotype test. Without prior infection with AAV helper virus, we unilaterally injected either BC-DG-RV (left) or saline (right). Both injections show signs of tissue damage along the injections' trajectories (increased autofluorescence), but no labelling of neuronal soma.

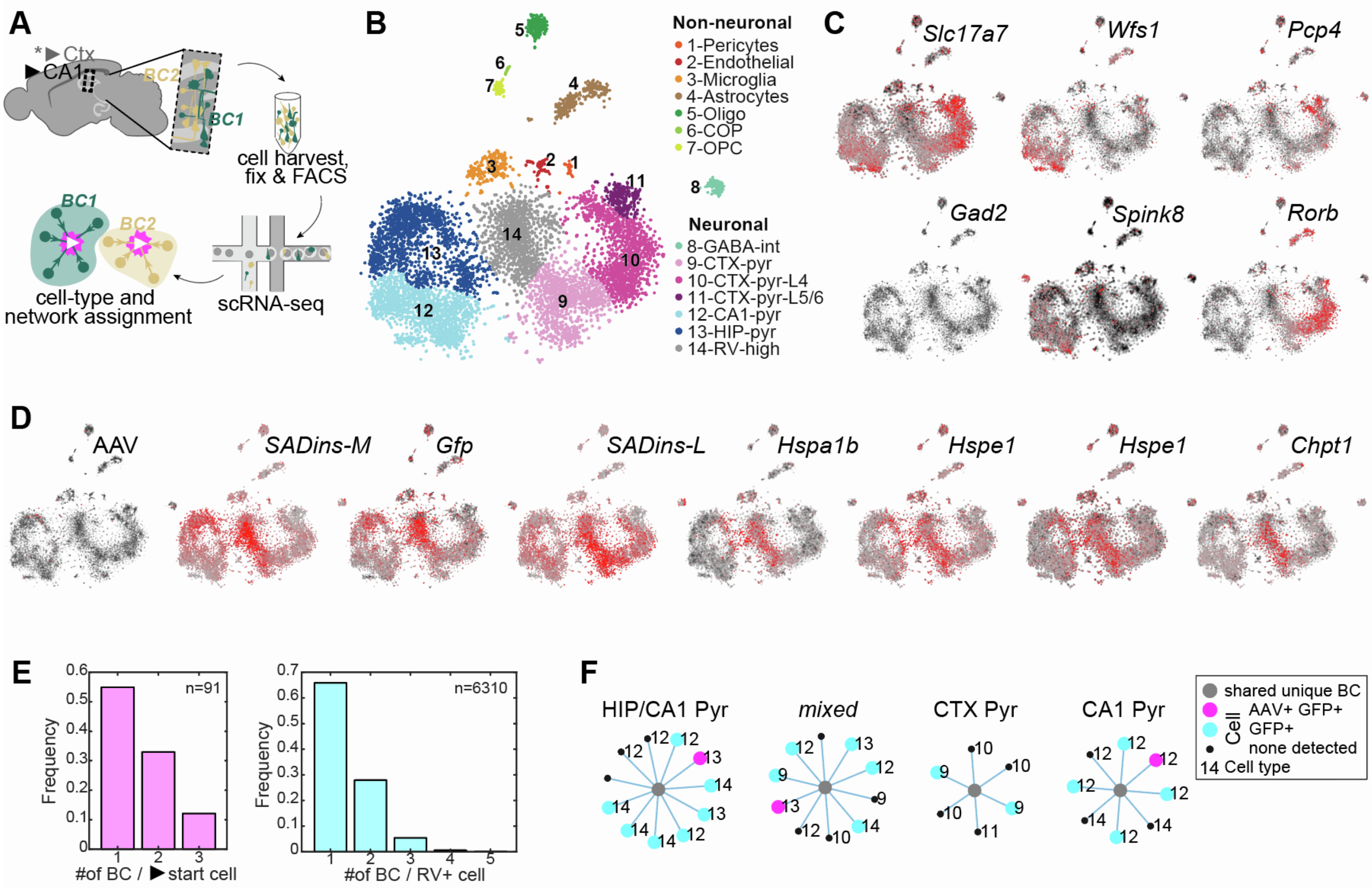

Figure S2. Single-cell RNA-seq on BC-DG-RV-infected neurons *in vivo*. Related to Figure 1.

(**A**) Experimental schematic, BC-DG-RV infection of hippocampus CA1, with secondary infection in isocortex dorsal to CA1, followed by dissociation, fixation, FACS and scRNA-seq; to simultaneously detect transcriptomes and BCs.

(**B**) tSNE visualization of 8032 cells, colored by cell type. Among 6979 neuronal transcriptomes, 129 were GABAergic interneurons, 2744 had a predominantly cortical pyramidal signature (1566 of which L4/5/6) and 2937 a hippocampal pyramidal signature (1418 of which CA1).

(**C**) Expression of six neuronal genes, in all cells visualized in (**B**). Grey, low; red, high; black circle, 0.

(**D**) In all cells visualized in (**B**), expression of AAV (any gene of *TVA, oG, mCherry*), and genes enriched to RV-high cluster 14. RV transcripts *SADins-M* and *Gfp* and *SADins-L*, and four further genes highly correlated with *SADins-M* expression. Grey, low; red, high; black circle, 0.

(**E**) Frequency of detecting single or multiple BCs in a single cell; in starter cells (91 neurons, as in Fig. 1F) or all BC-DG-RV+ cells (total, 6310 BC+ neurons).

(**F**) Four examples of potential single-cell networks; i.e., cells sharing a unique barcode. Over all networks, most have no verified starter cell (cell with AAV and GFP detected), and most have some degree of mixing of transcriptomic types from CTX and HIP, as shown in one example.

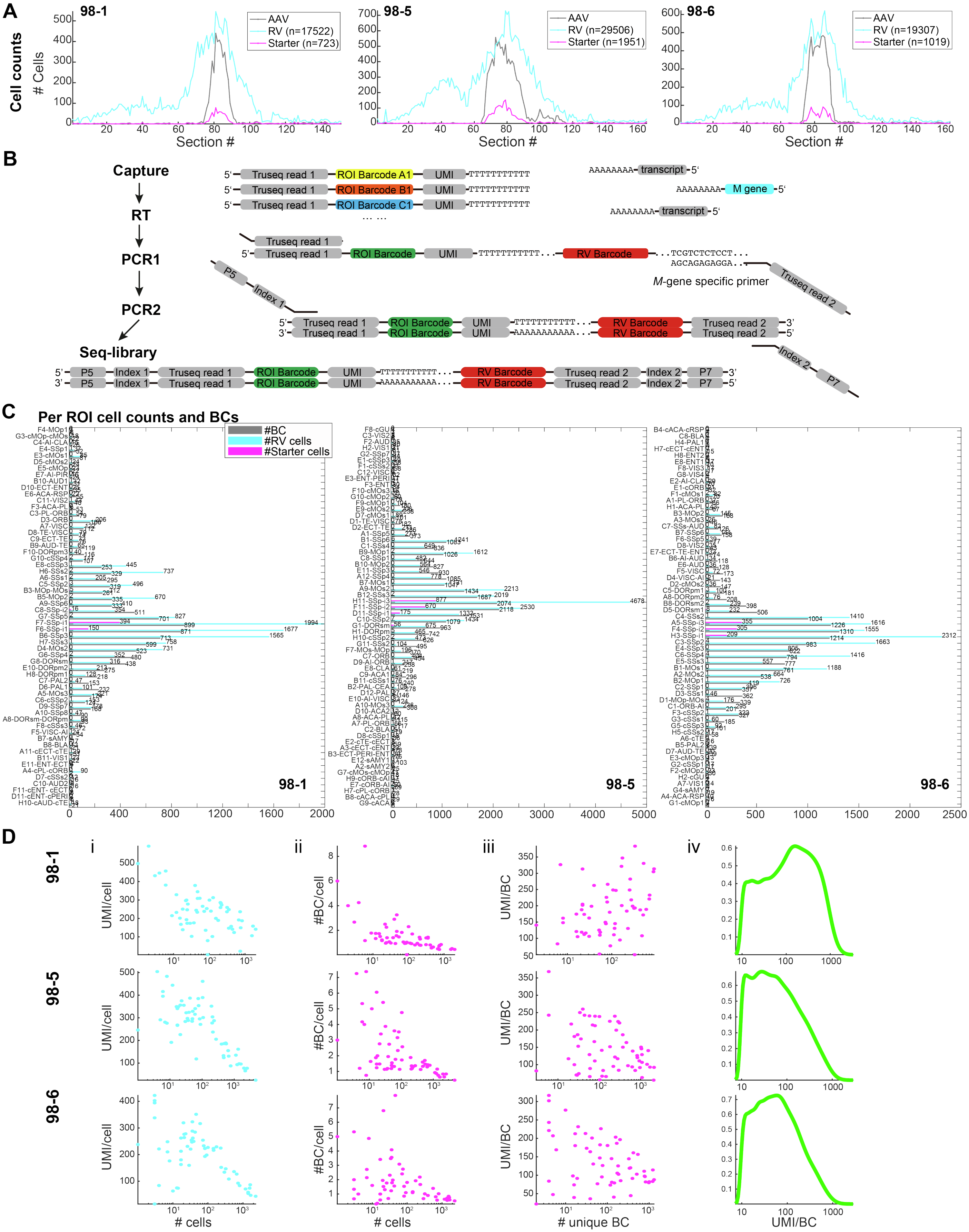

Figure S3. ROInet-seq analysis pipeline: Cell counts, workflow and raw BC detection. Related to Figures 2 and 3.

Figure S3, continued.

(**A**) After BC-DG-RV infection of the SSp, automated cell detection of AAV+ and RV+ cells across the full brain in anterior-posterior section order (50µm, gapless collection). Fluorescent overlap of signals (AAV+RV+) is considered a starter cell.

(**B**) Schematic of RT and BC-specific amplification from fixed tissue ROIs in ROInet-seq. Each ROI is collected to a separate well, loaded with lysis buffer and polyT primer carrying a unique ROI barcode (spatial index). In reverse transcription (RT), the indexed polyT captures polyA transcripts present in that ROI, including RV-genes such as *M* (with the network BC in its 3'UTR). cDNA is amplified in two consecutive PCR reactions, first using an oligo specific to a sequence flanking the *M*-gene (and BC), and then sequencing library prep to introduce library indexes and P5/P7.

(**C**) Summary of all ROIs per experiment, with imaging-based cell counts and BC detection.

(**D**) Per-ROI quantifications of cells, BCs and UMIs in ROInet-seq, per experiment (row). Each ROI is one dot (**i-iii**) (**i**) Scatterplot of UMI/cell as a function of number of cells detected (imaging). (**ii**) Scatterplot of BC/cell as a function of number of cells detected (imaging). (**iii**) Scatterplot of UMI/BC as a function of BCs. (**iv**) Distribution of copies (UMI) detected per unique BC.

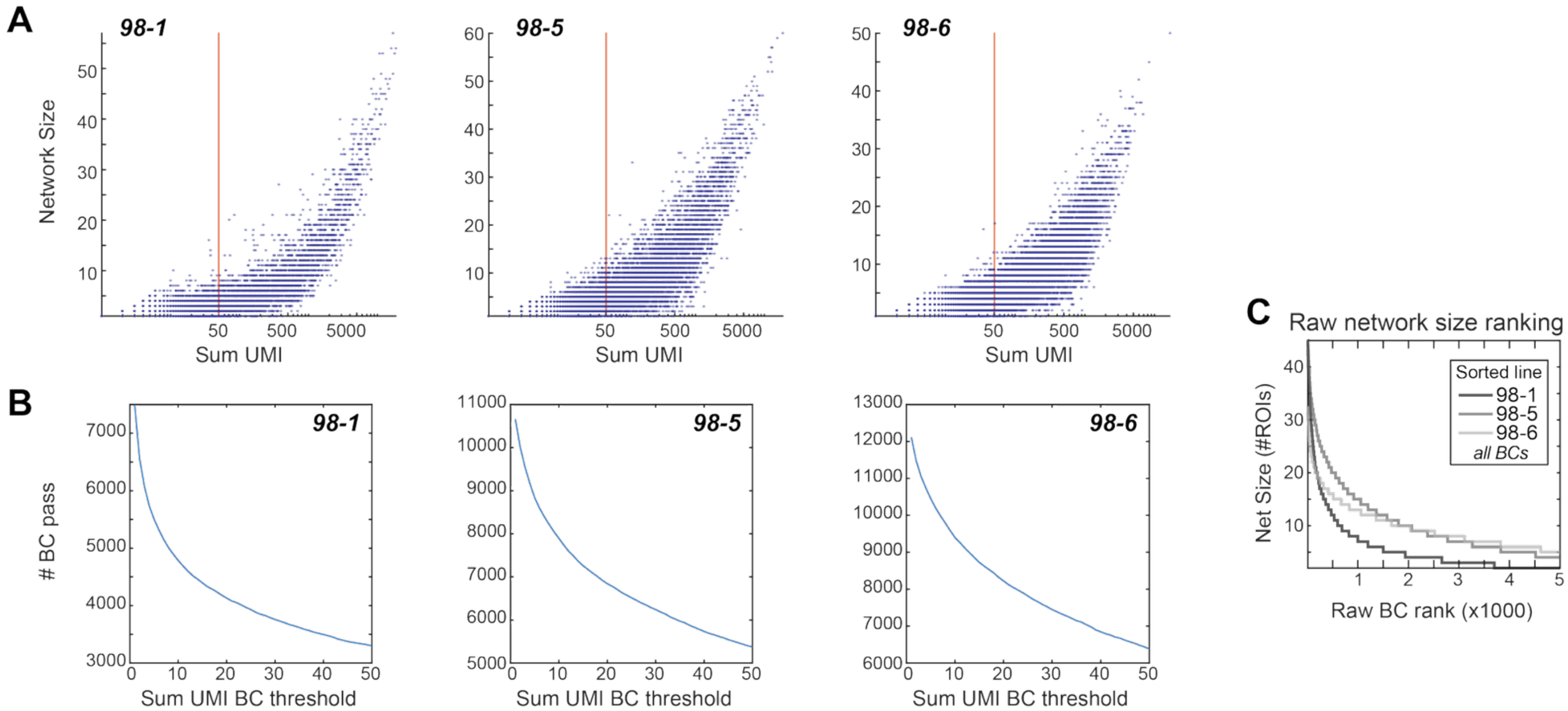

Figure S4. ROInet-seq analysis pipeline: BC detection and threshold evaluation for experiments 98-1, 98-5, 98-6. Related to Figure 4.

(**A**) For all BCs and pre-filtering, total number of UMIs and their potential network size (# of ROIs detected at ≥1UMI).

(**B**) Simulation of the number of BCs included in analysis, by Sum BC thresholds (total detected UMIs) of 1-50UMIs.

(**C**) BC ranking by network size, including all BCs (pre BC-thresholds).

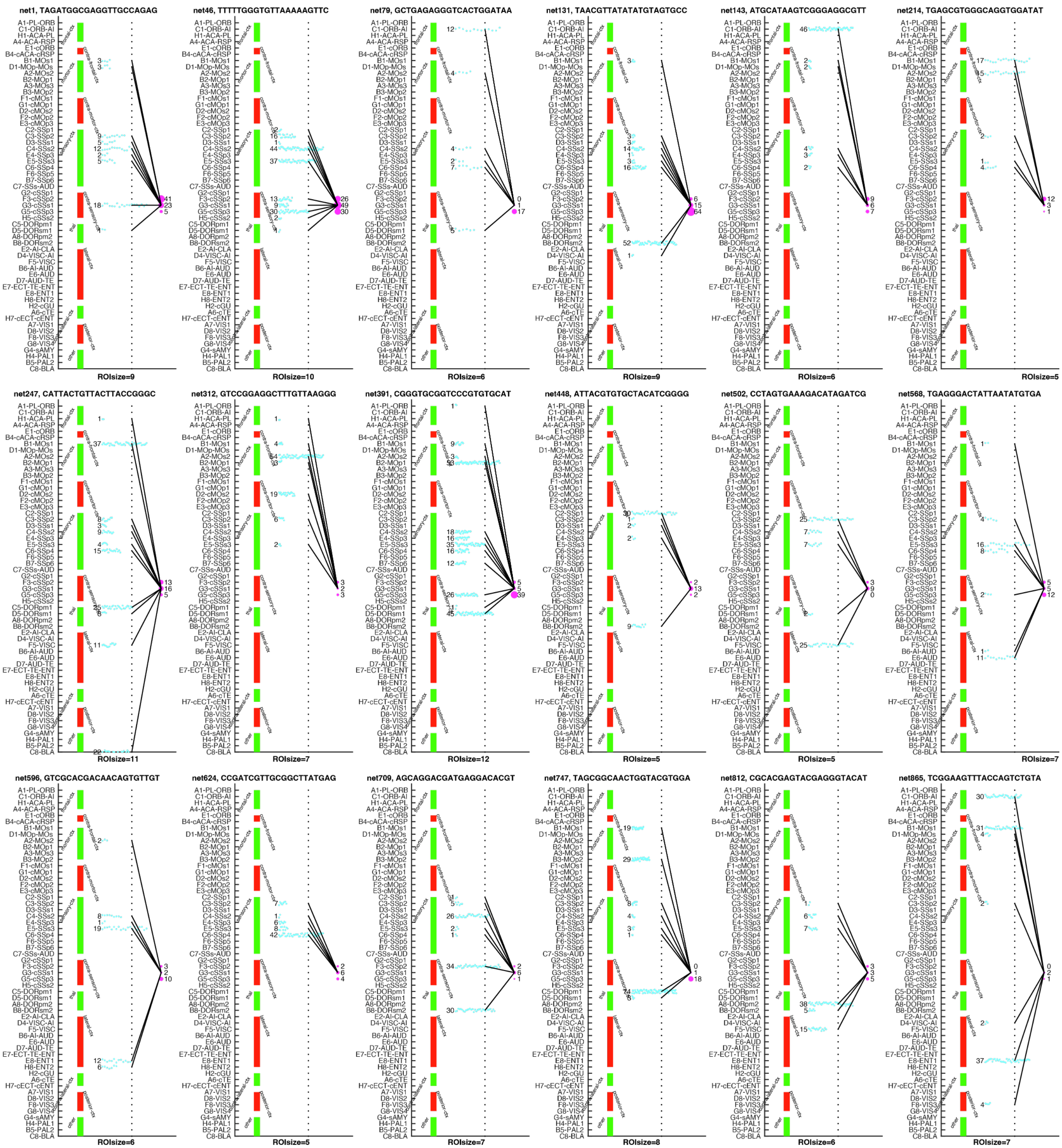

Figure S5. Input networks to single SSp neurons. Related to Figure 5.

Brain-wide input networks to 18 single SSp start site neurons in brain 98-6 at ROI resolution, visualized as dots, where each cyan dot represents detection of 10 unique network BC copies (UMI) in input ROIs, and magenta blob size represents unique BC copies in SSp start site.

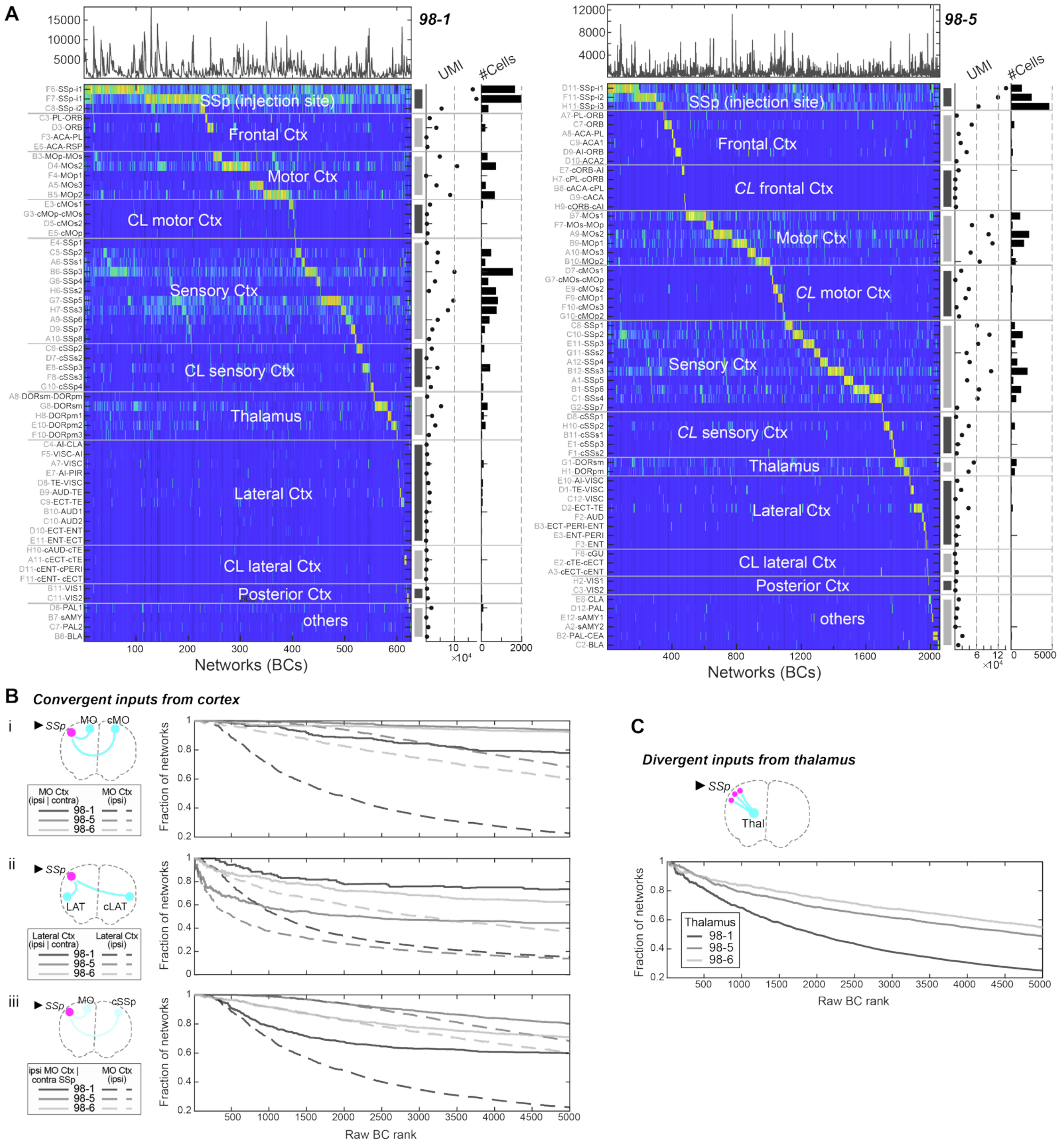

Figure S6. Brain-wide input networks and reproducible motives of convergent and divergent inputs to single SSp start site neurons. Related to Figure 5.

(**A**) Single-cell networks across all ROIs in brains 98-5, 98-6, ordered by dominantly detected ROI. Colormap represents network BC copies (UMI) (blue, low; yellow, high).

(**B-C**) Before thresholds, all BCs were ranked by their detection in #ROIs, from most to least widely spread BC. Lines indicate, per experiment, the fraction of networks that include the indicated ROIs; schematics to the left.

Figure S6, continued.

(**B**) Convergent inputs from ipsi- and contralateral cortical areas. Full lines show ipsilateral inputs conditioned on the simultaneous detection of contralateral inputs; i.e., the fraction of SSp neurons with ipsilateral inputs, given they received a contralateral input: (i) ipsi given contra in the MO cortex, (ii) ipsi given contra in the Lateral cortex, and (iii) ipsi MO cortex given contra SSp cortex. Dashed lines show the fraction of ipsilateral inputs alone; i.e., the fraction expected at chance. For bilateral input convergence within MO and Lat areas ((i) and (ii)), conditional and expected lines quickly separate; i.e., the convergent bilateral network motif is overrepresented (higher than expected). In (iii), in the case of the "unrelated" areas, ipsilateral MO inputs converge with contralateral SSp inputs at a rate as expected by chance (see long overlap of full and dashed lines in 98-5 and 98-6).

(**C**) Fraction of networks that include thalamus, showing that the thalamus maintains a high degree of connectivity to the SSp start site independent of rigorous BC thresholds or exclusion of smaller networks. As discussed in Fig. 3G, thalamic inputs to SSp were considered divergent as the regions had only ~4.5% of fluorescently labeled input cells, while ~57% of BCs were detected in the thalamus (after BC thresholds).

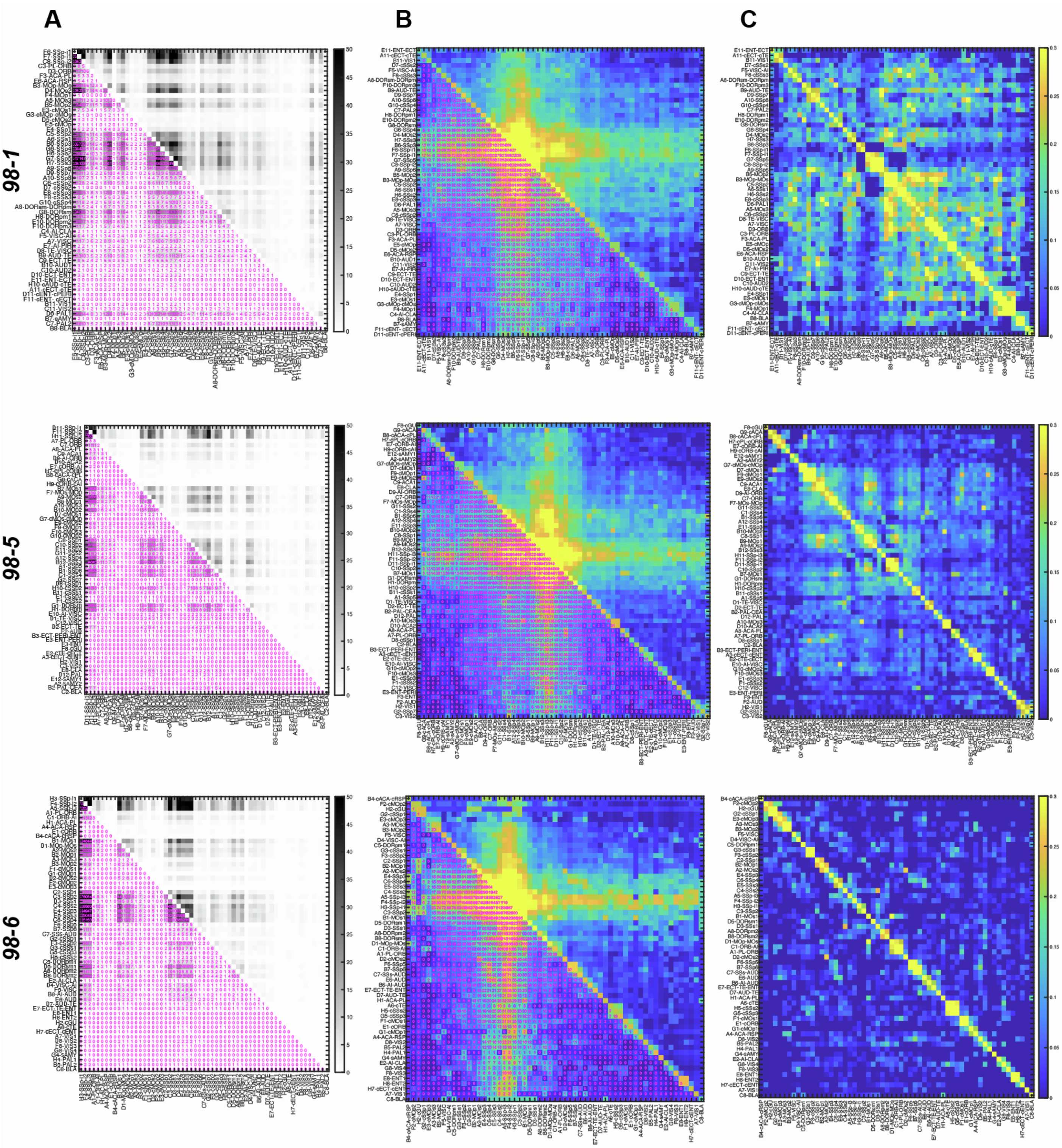

Figure S7. Brain-wide regional overlaps of SSp-cell network. Related to Figure 5.

Convergent regional inputs to single SSp start site neurons in brains 98-1, 98-5 and 98-6. Co-detection of network BCs between individual ROIs;

(**A**) Absolute overlap of all detected network BCs, in ROI order (as Fig. 5, S6);

(**B**) Absolute overlap of all detected network BCs, in order of similarity;

(**C**) As pairwise correlation, in order of similarity (as (**B**)).

Color-/greyscale represents min to max values normalized per ROI (excluding the diagonal).

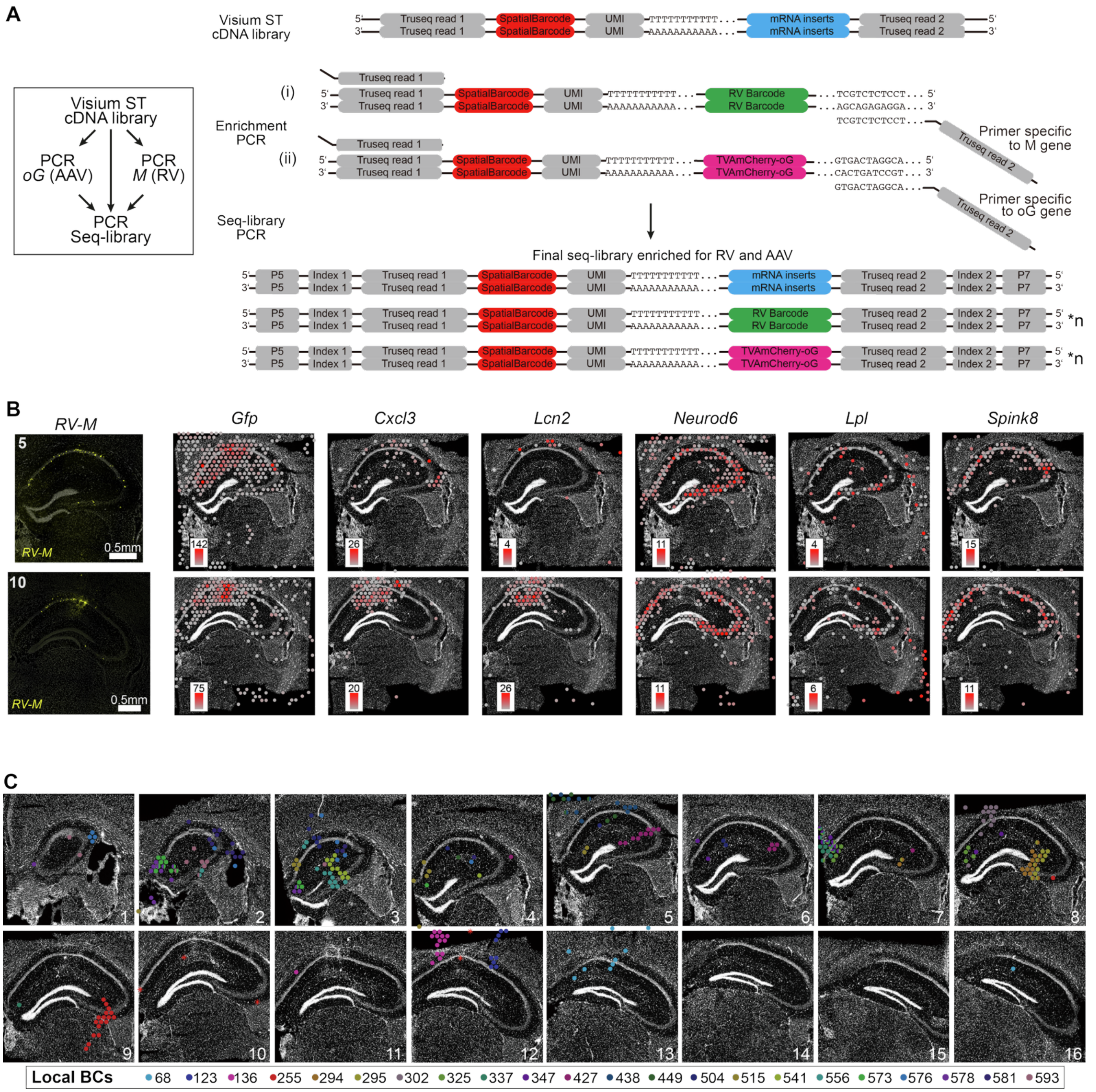

Figure S8. Visium Spatial Transcriptomics (ST) for increased resolution of local network reconstruction. Related to Figure 6.

(**A**) Workflow for amplification of RV (BC) and AAV (TVA) -related transcripts, from full cDNA.

(**B**) Visium ST gene expression of RV (*Gfp*), pyramidal neuron (*Neurod6, Spink8, Lpl*) and inflammatory markers (*Lcn2, Cxcl13*) in sections 5 and 10; with single molecule FISH-validation of RV matrix gene (*M*) expression in consecutive sections shown on the left. Grey, low; red, high; maximum UMI indicated per section. Scale bars, 0.5mm.

(**C**) Spatially resolved detection of 22 abundant, but locally restricted barcodes (blue in Fig. 4E), in 16 a.-p. hippocampus sections of Visium ST; white, nuclei counterstain (DAPI).

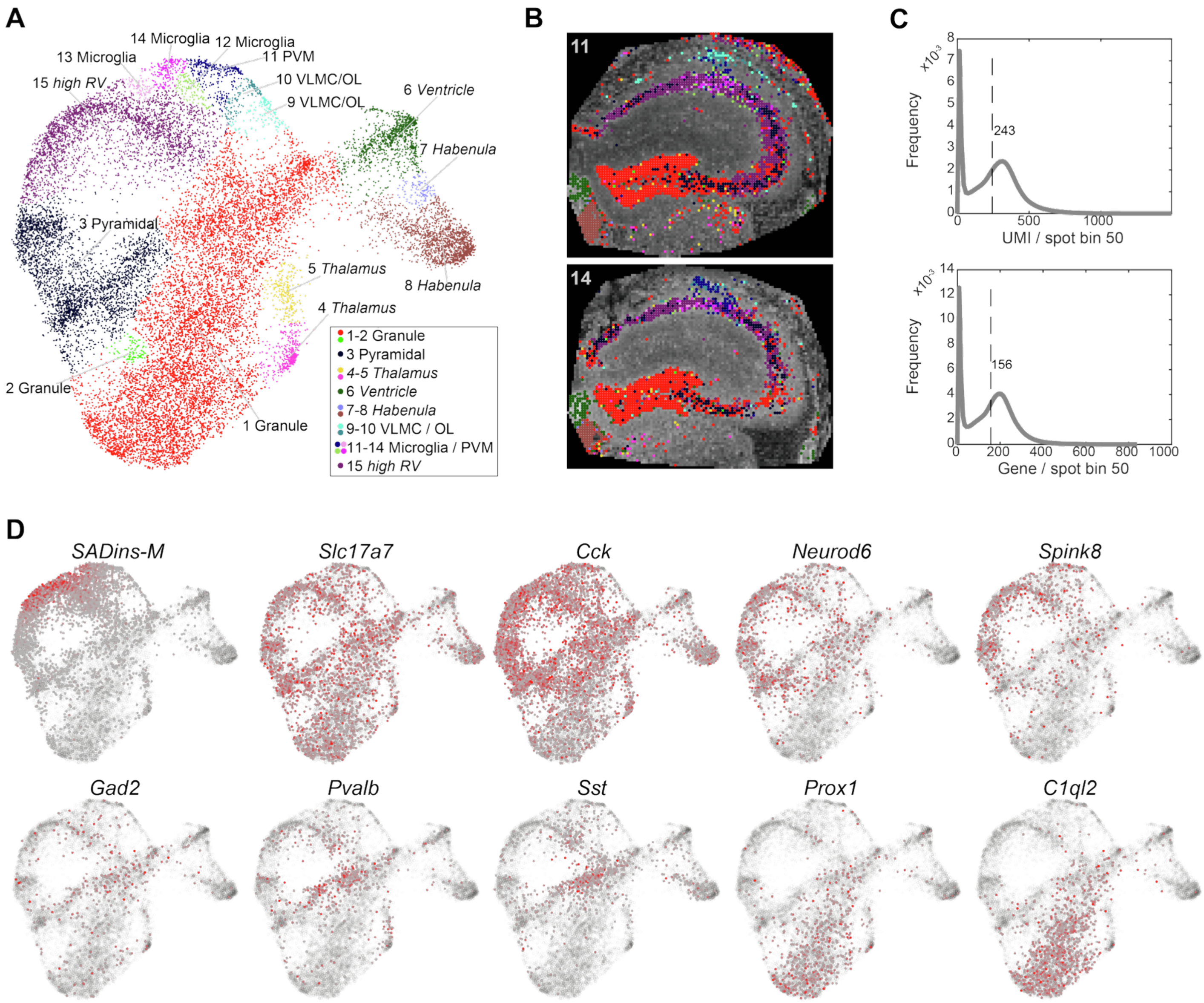

Figure S9. Clustering and gene expression in Stereo-seq STOmics. Related to Figure 7.

(**A**) UMAP clustering of spatial spots, bin 50, colored by molecular type.

(**B**) Molecular types according to UMAP clustering overlayed on sections 11 and 14.

(**C**) UMI and genes detected (bin 50) with median values indicated.

(**D**) Detection of the SAD-B19 RV-transcript M, and excitatory neuron markers (*Slc17a7, Cck*, *Neurod6, Spink8*)*,* GABAergic type markers (*Gad2, Pvalb, Sst*) and granule markers (*Prox1, C1ql2*).

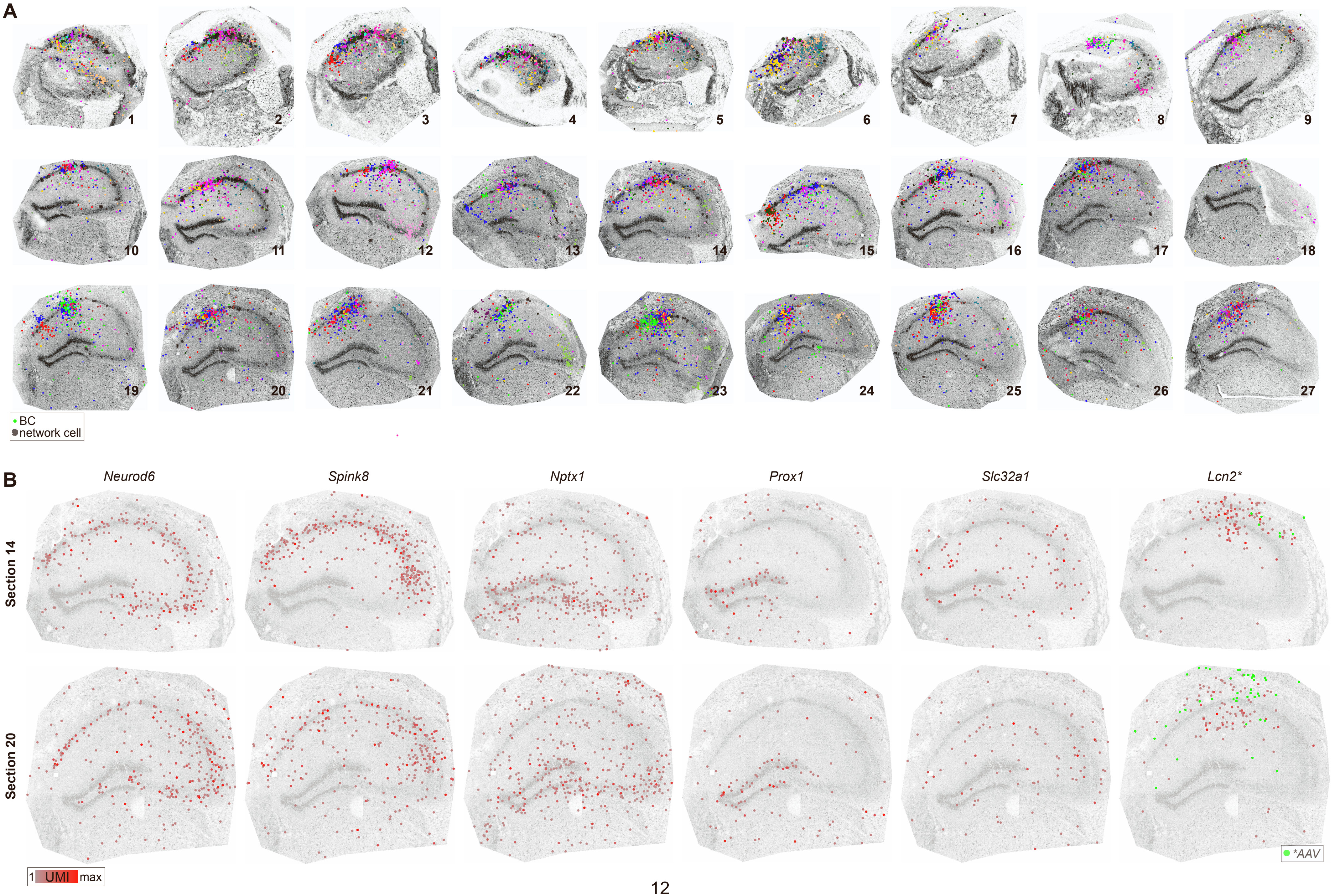
A
1
2
3
4
5
6
7
8
9
10
11
12
13
14
15
16
17
18
19
20
21
22
23
24
25
26
27
BC
network cell
B
Neurod6
Spink8
Nptx1
Prox1
Slc32a1
Lcn2*
Section 14
Section 20
*AAV
1 UMI max

Figure S10. Cell-resolved hippocampus networks on Stereo-seq STOmics, Related to Figure 7.

(**A**) Spatially resolved detection of the top 20 abundant barcodes (colors), in all 27 a.-p. hippocampus sections. Black, network cell outlines. The grey scale showing section anatomy is based on total detected molecules; where dark is the maximum detected molecules.

(**B**) Spatial gene expression of neuronal markers *Neurod6* (pyramidal), *Spink8* (CA1-CA2), *Nptx1* (CA3), *Prox1* (DG), *Slc32a1* (inhibitory) and inflammatory marker *Lcn2*, as well as AAV-transcripts (green) to indicate the injection site, in sections 14 and 20. Maximum spot expression (bin 1) is 4 UMI, except *Slc32a1* section 14 (max=3) and *Nptx1* (max=5 both sections). Grey scale, total detected molecules; where dark is maximum.
