# Supplementary material for: Mapping brain-wide monosynaptic inputs to single neurons with ROInet-seq
Source: Cell Rep Methods. 2026 Jul 7;6(9):101512. doi: 10.1016/j.crmeth.2026.101512 (PMC13615506; doi:10.1016/j.crmeth.2026.101512)
Supplement: Supplementary file 2 [file mmc3.pdf]

Cell Reports Methods

Article

Mapping brain-wide monosynaptic inputs to single neurons with ROInet-seq

Graphical abstract

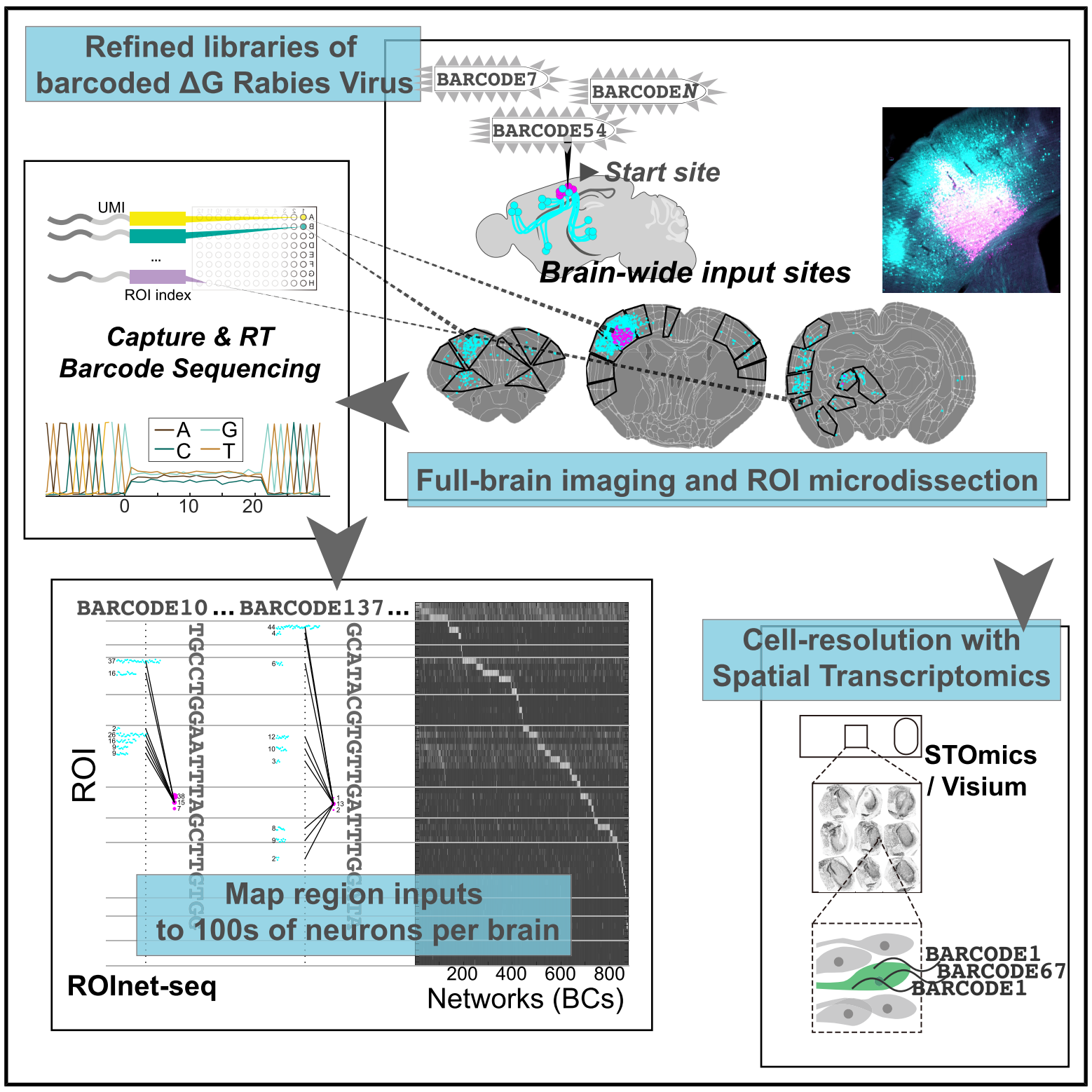

Authors

Zhige Lin, Osnat Ophir, Thorsten Trimbuch, ..., Christian Rosenmund, Hannah Hochgerner, Amit Zeisel

Correspondence

hhoch@campus.technion.ac.il (H.H.), amit.zeisel@technion.ac.il (A.Z.)

In brief

Lin et al. introduce ROInet-seq, a scalable and low-cost spatial sequencing approach that combines barcoded rabies virus tracing with region-of-interest sampling to map single-neuron input networks. The method enables brain-wide connectivity profiling following standard histology workflows and reveals structured network motifs.

Highlights

- Highly complex and uniform barcoded rabies virus for monosynaptic tracing
- Full-brain fluorescent imaging and region-of-interest network barcode sequencing
- Brain-wide reconstruction of parallel short- and long-range inputs to single neurons
- Convergent cortical inputs and divergent thalamic inputs to SSp neurons

Lin et al., 2026, Cell Reports Methods 6, 101512
September 21, 2026 © 2026 The Author(s). Published by Elsevier Inc.
https://doi.org/10.1016/j.crmeth.2026.101512

CellPress

Cell Reports Methods

CellPress
OPEN ACCESS

Article

Mapping brain-wide monosynaptic inputs to single neurons with ROInet-seq

Zhige Lin,[1] Osnat Ophir,[1] Thorsten Trimbuch,[2,3] Bettina Gann,[2,3] Muhammad Tibi,[1] Hermann Schmidt,[5] Christian Rosenmund,[2,3,4] Hannah Hochgerner,[1,*] and Amit Zeisel[1,6,*]

[1]Technion – Israel Institute of Technology, Faculty of Biotechnology and Food Engineering, Haifa 320003, Israel
[2]Viral Core Facility, Charité-Universitätsmedizin, Berlin 10117, Germany
[3]Institute of Neurophysiology, Charité-Universitätsmedizin, Berlin 10117, Germany
[4]NeuroCure Cluster of Excellence, Charité-Universitätsmedizin, Berlin 10117, Germany
[5]DNA Cloning Service, Hamburg 22609, Germany
[6]Lead contact
*Correspondence: hhoch@campus.technion.ac.il (H.H.), amit.zeisel@technion.ac.il (A.Z.)
https://doi.org/10.1016/j.crmeth.2026.101512

MOTIVATION Several strategies have been deployed to map regional connectomes of mammalian brains. Monosynaptic rabies virus (ΔG-RV) tracing defines presynaptic inputs but relies on microscopy and cannot resolve many single neurons in parallel. Even though barcoded rabies virus strategies and spatial transcriptomics offer potential solutions, existing approaches remain limited in throughput, cost, and feasibility for brain-wide analysis. There is, therefore, a need for accessible, scalable methods that enable parallel reconstruction of single-neuron input networks across large spatial domains.

SUMMARY

Viral projection tracing strategies help build regional connectomes of mammalian brains. G-deleted rabies virus (ΔG-RV) establishes monosynaptic input connectivity but cannot distinguish networks at cell resolution. Here, we implement a barcoded ΔG-RV for network tracing by quantitative RNA-sequencing. At optimized library complexity and uniformity, barcode detection reliably distinguished individual monosynaptic input networks of multiple infected neurons in parallel. To scale this approach to hundreds of cells and full-brain inputs, we develop regions-of-interest network sequencing (ROInet-seq)—an accessible, scalable, and low-cost spatial assay. ROInet-seq combines routine fluorescent imaging of fixed tissue sections with targeted barcode sequencing, enabling brain-wide mapping of single-neuron networks. In the somatosensory cortex, the assay reveals dominant regional contributions that inputs from a handful of thalamic neurons diverged to multiple neurons and frequent convergent ipsilateral and contralateral cortical inputs. Integration with commercial spatial transcriptomics assays improves resolution, together establishing a scalable framework to resolve single-cell network architectures.

INTRODUCTION

Neuronal networks are the biological basis for all higher functions and behaviors in animals. Tasks such as memory acquisition and retrieval or sensing and processing social cues require extraordinary flexibility, versatility, and computational power. These capabilities are achieved by the brain's multiregional circuits and local modifying networks of neurons, but revealing the architecture of their networks remains a major undertaking. Mapping neuronal networks has been approached across different scales of connectivity. At the most detailed level, great efforts are undertaken to reconstruct detailed maps of single neurons and their complete synaptic connectivity using transmission electron microscopy[1–3] or expansion microscopy.[4,5] Naturally, these approaches remain highly complex and costly. Combining electrophysiology with scRNA-seq (e.g., Patch-Seq[6]) can measure neural connectivity and identity simultaneously, but it is typically limited to several pairs of neurons. Long-range projections between distant brain regions cannot be recorded, making it challenging to obtain a complete map of single-cell neuronal connections. On the other end of the scale, viral projection tracing uses modified neurotrophic virus, such as fluorescent marker-expressing adeno-associated virus (AAV). Depending on the virus' tropism, the region-of-interest's (ROIs') input or output regions are then fluorescently labeled, revealing brain-region connectivity on a mesoscale level.[7,8] However, these projection maps generally do not reveal synaptic connectivity and lack single-neuron resolution.

© 2026 The Author(s). Published by Elsevier Inc.
This is an open access article under the CC BY license (http://creativecommons.org/licenses/by/4.0/).

In sum, connectivity tools ideally map synaptic neuronal networks of single-neurons across substantial volumes of a mammalian brain but typically navigate concessions between resolution and scale. Transsynaptic viral tracing strategies circumvent some limitations by visually labeling neurons that are synaptically connected to each other. To map synaptic input networks, a genetically modified virus (for example, G-deleted rabies virus [ΔG-RV]),[9] is injected to a site of interest (start site), and when provided with G *in trans*, can transfer to input neurons via its synapses. In this manner, ΔG-RV labels neurons infected in the start site and their input neurons, revealing monosynaptic-connected input networks across full brain volumes *in vivo*. When multiple fluorophores and selective receptors are used, independent circuits can be revealed in mutliplex.[10] Yet, typically, multiple cells are labeled with the same fluorescence tag, which hinders distinguishing input networks of single neurons in the brain. Similar to a modification used in viral projection tracing,[8,11,12] several groups, therefore, recently developed a variant of ΔG-RV that carries a library of unique genetic barcode (BC) sequences. BCs are read by sequencing, and they increase the number of distinguishable markers that uniquely label individual networks in a single experiment. This allowed tracking glial interactions[13] and monosynaptic retrograde neuronal connectivity *in vitro.*[14] *In vivo,* two groups[15,16] recently used *in situ* sequencing to read network BC sequences and characterize the cells' molecular identities besides providing valuable spatial context of single cells in the network. This method is practically limited to small areas or local networks and costly to implement at scale. Despite some limitations, the genetic barcoding strategy shows great potential in improving the resolution and scale of neuronal connectivity mapping.

Here, we generated high-complexity libraries of distinguishable rabies virus particles by inserting a 21 nt random BC into the 3′ UTR region of RV-matrix (*M*) gene. We characterized *in vivo* properties of barcoded ΔG rabies virus (BC-ΔG-RV) in droplet-based scRNA-seq and detected network BC and cell transcriptomes simultaneously. We then implemented sequencing-based assays that navigate the concessions between resolution and scale to resolve synaptic inputs to single cells. At the center of these efforts, we developed ROInet-seq, an accessible, coarse spatial assay compatible with standard histology and molecular biology tools, to reconstruct single-neuron input networks at low sequencing costs. We also demonstrated how commercial spatial transcriptomics (ST) assays (Visium ST and STOmics Stereo-seq) can improve the method's resolution of local networks in densely wired brain regions. Our work provides quantitative and qualitative insights into cortical connectivity, including the robust occurrence of simultaneous inputs from multiple distal sites to a single cortical cell, and distinct, region-specific network cytoarchitectures.

RESULTS

To insert a 21 nt random BC to the RV genome at an optimal position, we first tested the genome's coverage in ΔG-RV-infected HEK cells using droplet-based 3′ scRNA-seq by 10× Genomics. Of five genes in the ΔG-RV genome (*N*, *P*, *M*, *GFP*, and *L*), matrix protein-encoding *M* achieved the highest coverage, and we inserted the BC at its 3′ UTR (Figure 1A). Then, rabies particles were recovered from HEK-cell transfection with the BC-ΔG-RV plasmid library and pseudotyped with the avian envelope EnvA (Figure 1B, see STAR Methods). For uniform representation of all BCs in the libraries of RV plasmids and particles, it was vital to eliminate bias introduced at any step of the protocol (Figures 1C and 1D). Low-complexity and/or non-uniform libraries, such as when few BCs are overrepresented, would lower the confidence that a given detected BC in fact represents a single, initially infected starter cell. Therefore, we first introduced several measures to achieve highly upscaled uniform RV plasmid and particle production (see STAR Methods).

For example, to maximize RV particles titer for *in vivo* tracing, plasmid-transfected HEK cells are typically amplified multiple times. However, during amplification, any fast-growing subset of cells would lead to an overrepresentation of the BCs it produces. Highly amplified particles then occupy a large portion of the entire library (high frequency), and depending on sequencing depth, would, in practice, reduce overall library complexity (e.g., library 16.1, Figures 1C and 1D). To overcome this limitation in subsequent BC-ΔG-RV libraries, HEK cells were transfected with the RV-plasmid library on a large, parallel scale, and we limited production time to a single round rather than amplifying cells multiple times (e.g., libraries 16.4, 16.5, 16.6, 17.2; Figures 1C and 1D). This balanced cell growth and drastically improved uniformity of the barcoded viral particles library. The optimized production strategy resulted in final libraries with acceptable uniformity. Measured by unique molecular identifier (UMI)-based amplicon sequencing, the top BCs occupied ∼0.1%–1% of the total library, and the probability of multiple cells infected by the same BC at infection of around 200 starter cells was 10% or less (Figures 1C and 1D).

We observed 2–6× lower titers of BC-ΔG-RV in virus production, hinting that insertion of a 21 nt BC to the 3′ site of *M* may have compromised the virus' replication or packaging capacity. Therefore, we next tested whether *in vivo* retrograde transfer efficacy may have been compromised too. Indeed, compared with standard ΔG-RV, the BC-ΔG-RV showed overall lower labeling efficacy of neurons in the mouse hippocampus CA1 and decreased efficiency of monosynaptic transmission to CA1 input cells (i.e., presynaptic-per-starter cells). At the same time, variability between animals and experimental batches was large for both virus variants (Figure S1).

To assess how BC-ΔG-RV performs at cell resolution *in vivo*, we isolated BC-ΔG-RV-infected cells for single-cell (sc) RNA-seq (Figure S2). We harvested one brain 8 days after BC-ΔG-RV injection to the CA1, then gently microdissected and dissociated fluorescent regions. Cell suspensions were quickly fixed, sorted by fluorescence-activated cell sorting (FACS), and BC-ΔG-RV (GFP+) cells were processed for scRNA-seq (10× Chromium). Out of 6,310 recovered neurons, 66.4% cells contained BC-ΔG-RV transcripts, and 1.4% were confirmed starter cells (i.e., they also expressed genes derived from the AAV helper virus). This means that on an average, each starter cell transynaptically labeled 47.4 input cells (''presynaptic-per-starter''), which was in line with quantifications from imaging (Figure S1). RV-labelled network cells were predominantly glutamatergic cortical or hippocampal pyramidal cells. Neurons with particularly high

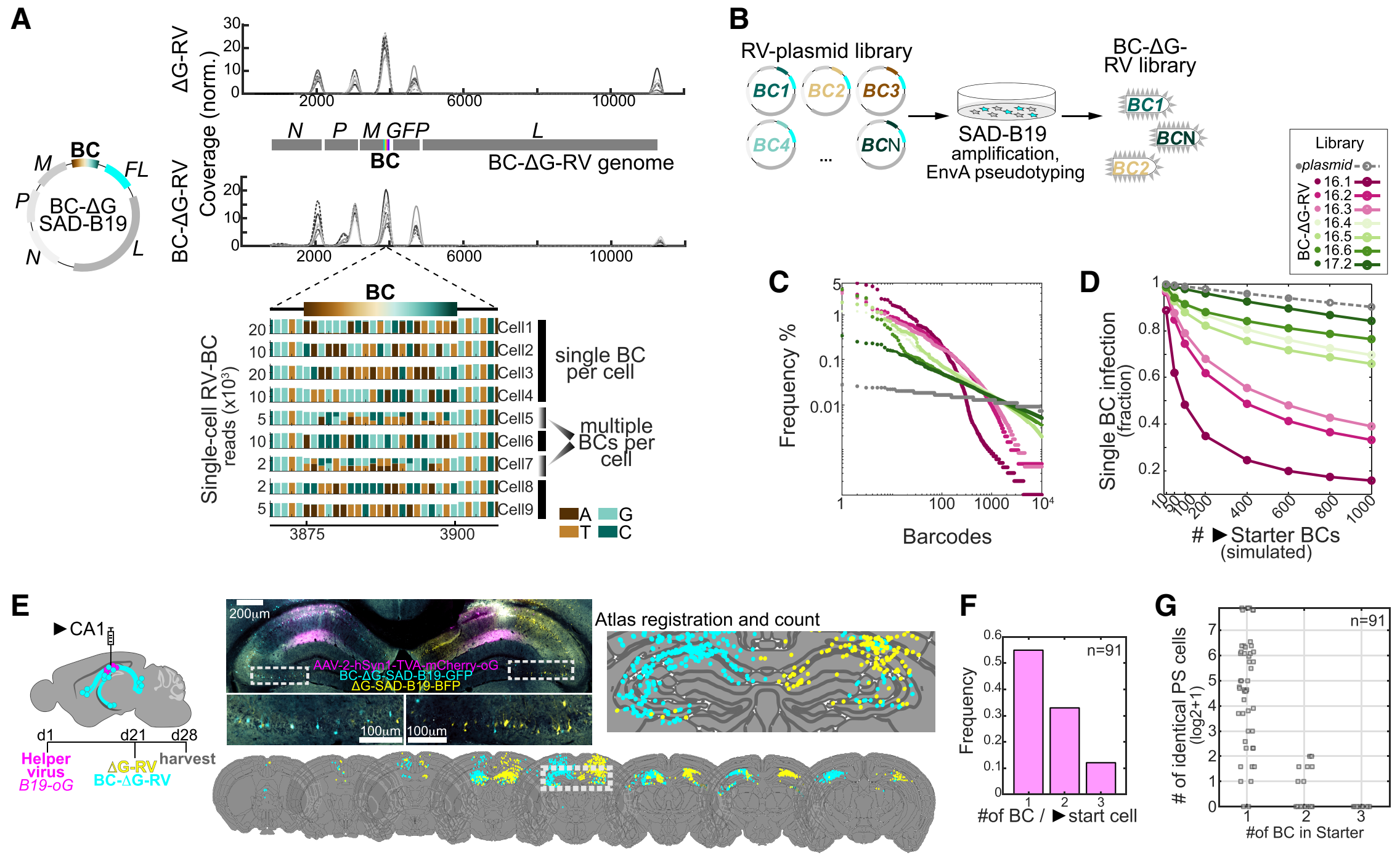

Figure 1. Generation of barcoded G-deleted rabies virus and *in vivo* application for network tracing

(A) A 21 nt random barcode was inserted to the 3′ site of the highly detected *M* gene in G-deleted SAD B19 rabies virus. Right: coverage of the rabies genome in 10× Chromium 3′ scRNA-seq in ΔG-RV and BC-ΔG-RV. Bottom: reads in the BC region from 9 single BC-ΔG-RV-infected cells, detected by 10× Chromium 3′ scRNA-seq. Most cells contained a single barcode; others contained multiple barcodes.

(B) The BC-ΔG-RV library is recovered, amplified, and pseudotyped with avian envelope (EnvA).

(C) Frequency of the top 10^4 detected barcodes in the RV-plasmid library and increasingly more complex BC-ΔG-RV libraries.

(D) Simulation of the expected cell fraction with singlet infection of increasingly more complex BC-ΔG-RV libraries and hypothetical comparison to the RV-plasmid, as a function of starter cells number.

(E) Histological comparison of ΔG-RV and BC-ΔG-RV *in vivo*. Equal titers (0.8×10^8IU/mL) of BC-ΔG-SAD-B19-GFP and ΔG-SAD-B19-BFP control each, injected to unilateral CA1, display similar infection and transmission patterns.

(F) In single-cell RNA-seq, frequency of detecting single or multiple BCs in a single starter cell (91 neurons).

(G) In single-cell RNA-seq, scatterplot of 91 verified starter neurons, with 1–3 unique BCs detected, and the indicated number of presynaptic cells with the same BC (combination).

expression of RV transcripts also had stress-related signatures (e.g., heat shock protein-encoding), and lower expression of neuronal marker genes compromised finer subtyping (Figure S2). We observed that in the start site, multi-BC-infected starter cells were nearly as common as single BC-infected starter cells, as 45% of all starter cells had multiple BCs (Figures 1F and S2). It was also observed that 34% of all RV+ cells had >1 BC (Figure S2). However, since most of these had BC combinations that did not overlap with those observed in any individual starter cell, BC combinations likely resulted from RV+ cells participating in multiple networks. Only in a minority of cases did we observe that multi-BC-infected starter cells transferred more than one BC to their input networks (Figure 1G). For example, out of 30 starter cells with 2 BCs, 76% transferred only one BC to input neurons. The combination of same 2 BCs was detected only in 7 input networks and in no more than 3 neurons per network, which was well below the average network size. Observing the same cell's connection multiple times due to multi-BC infection, therefore, likely accounts only for a minority of cases and is unlikely to overestimate connectivity. Finally, in the 91 sequenced starter cells, we did not detect incidents of either multi-cell infection (i.e., one BC infecting several starter cells) or connected starter cells (i.e., starter cell-to-starter cell transmission), as none of the 91 starter cells contained reads of the same BC sequence.

Next, we aimed to apply BC-ΔG-RV to characterize single-neuron input networks *in vivo.* Here, we assumed that each BC virtually represents a single neuron, based on the following observations: (1) each starter cell was dominantly infected by one or several BC-ΔG-RV particles, where only one is likely to be transferred to most input neurons; (2) the BC library was highly complex, such that BCs can serve as a unique molecular tag; and (3) BCs could be captured and reliably read by a universal RNA-seq protocol. Therefore, with good characterization of BC-ΔG-RV *in vivo* performance and important improvements in library uniformity, we concluded that detection of a unique BC in a cell or region *in vivo* faithfully described synaptic input of that cell or region to the start site. We argued that reconstructing input networks to a start site of interest required a sensitive, accessible method scalable to the full volume of the brain,

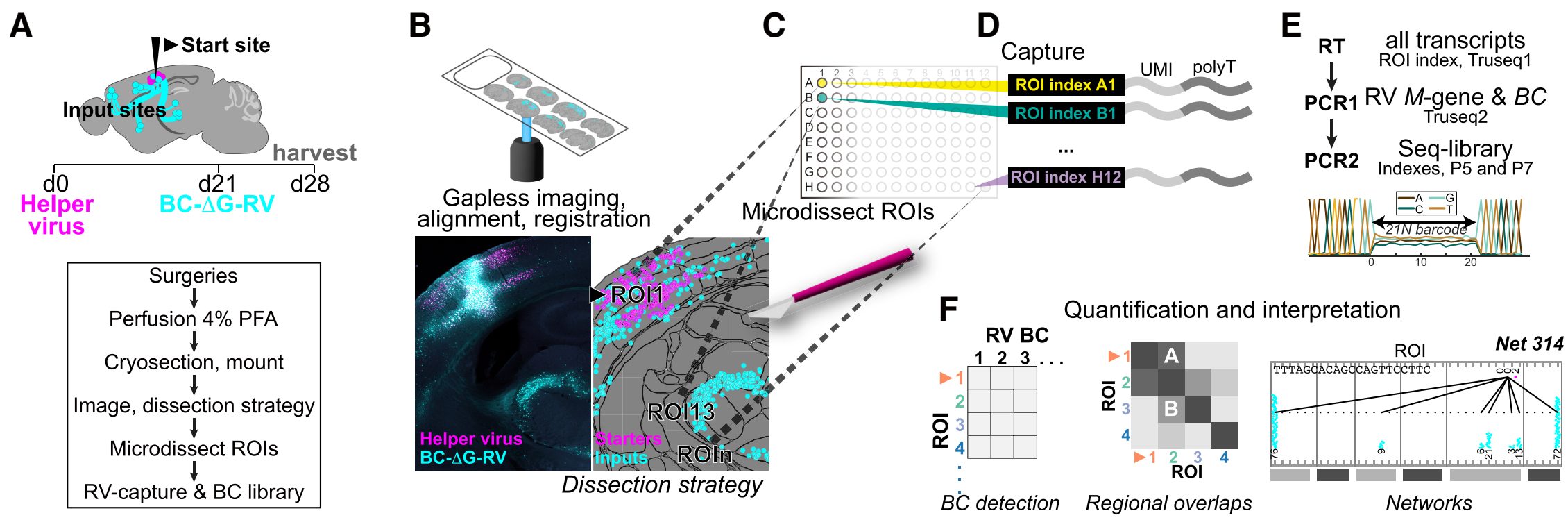

Figure 2. ROInet-seq for full-volume cell-resolved region-of-interest input network maps to the somatosensory cortex

(A and B) Experimental outline. Top: helper virus was injected into the starter site on day 1, followed by BC-ΔG-RV on day 21; brains were harvested on day 28 by 4% PFA perfusion (A). Brains were then post-fixed, cryoprotected, cryosectioned, mounted, and imaged through the full volume (B). All sections are aligned to the Allen Mouse Brain Atlas, and cells/ROInets are registered and quantified, informing the dissection strategy. Note: after registration (left), magenta represents starter cells (AAV+RV+). AAV+RV− cells are disregarded.

(C and D) ROIs are microdissected (C) and transferred to individual wells in a multiwell plate containing lysis buffer with barcoded polyT-capture oligos (D) to introduce a single barcode to all poly-adenylated transcripts of the same ROI.

(E) Reverse transcription, targeted PCR to amplify the RV-*M* gene with the BC fragment and PCR for final sequencing libraries. Bottom: pseudo-Sanger sequencing view of the nucleotide stretch containing the barcode sequence, as sampled by ROInet-seq.

(F) Sequencing results in a list of unique detected ΔG-RV barcodes per ROI with molecular counts, allowing reconstruction of (higher-order) monosynaptic input networks.

and given the limited network cell recovery and reduced transcriptomic fidelity in scRNA-seq observed by us (Figure S2) and others,[15,17] a spatial assay on fixed tissue was preferable over cell isolation. Therefore, we developed "region of interest-network sequencing", or ROInet-seq, where we specifically sequenced all BCs from individual BC-ΔG-RV-infected regions after full-volume fluorescent imaging. Briefly, 7 days after BC-ΔG-RV surgeries, brains were harvested by transcardial paraformaldehyde (PFA)-perfusion, cryoprotected, cryosectioned (50μm), mounted, and imaged (Figures 2A and 2B). A semi-automated pipeline achieved full-brain registration to the Allen Mouse Brain Atlas (hereinafter referred to as "atlas") and cell counting. Based on this analysis, ROIs were selected and microdissected (Figures 2B and 2C). Each ROI was collected to lysis mix, where all transcripts were captured with an individually indexed poly-T capture oligo (ROI-index), reverse transcribed, and the RV-*M* gene (flanked by the BC sequence) is specifically amplified (Figures 2D, 2E, and S3). Finally, ROIs were pooled and processed for sequencing (Figure S3 and STAR Methods). Each experiment resulted in an array of unique BCs (rows) on ROIs (columns), where entries are molecule counts (UMIs). Further analysis could extract pairwise ROI BC overlaps, input networks of individual starter cells, and so forth.

In three mice, we targeted the primary somatosensory cortex (SSp) (Figure 3A). We imaged virus-labelled cells along the full brain volume and performed atlas registration and cell detection for all sections, resulting in per-region cell counts (Figures 3B and S3). For all three brain samples, starter cells (AAV+RV+) primarily mapped to the deep layers of the barrel field (SSp-bfd), with some starter cells also detected in the adjacent nose field (SSp-n) (Figure 3B). The majority of input cells (AAV-RV+) came from ipsilateral and contralateral cortical sites within the SSp, the secondary somatosensory cortex (SSs) and the primary and secondary motor cortex areas (MOp, MOs), and ipsilateral subcortical regions as the thalamus (ventral posterior- [VP] and posterior [PO] complexes) and striatum (caudoputamen, CP).

Based on the cell counts and atlas alignment, we designed a consistent microdissection strategy and collected >60 ROIs along the full anterior-posterior axis of the forebrain, per brain (Figures 3C and S3; Table S1). All amplified BC fragments were sequenced with UMIs. For each brain, we analyzed 941–2763 putative networks, based on the most confident and widely detected unique BCs (Figures 4A, 4B, and S4; STAR Methods). Size and network participation of ROIs greatly varied, but for ROIs in all three brains, the count of UMIs and unique BCs both scaled with the number of cells detected per ROI (Figures 3D and S3): On average, ROInet-seq detected ∼100 copies (UMIs) of 1 BC per cell; although this ratio varied between approximately 10-1,000. We further observed that BC detection was not binary. Instead, when considering individual BC's molecule counts, BC abundances in all ROIs followed non-uniform distributions (Figure 3E). This implied that RV-transmission was not uniform and suggested that regarding BC detection as a binary rather than a quantitative feature would be simplistic.

Detection of BCs, starter cells, and input cells was reproducible between ROIs of the three brain samples (Figures 3F and S3). Unsurprisingly, almost all unique BCs were detected in the SSp start sites, but most unique BCs were detected in other ROIs, too (Figure 3G). This indicated that starter cells usually received inputs from outside their immediate neighborhood (start site). Ipsilateral SSp ROIs were most connected, recapitulating observations of a recent RV retrograde tracing study, where single electroporated SS-bfd neurons received ∼80% of their inputs from local SSp cells.[18] Yet, other regions also showed great overlap: ROIs in motor areas contained between

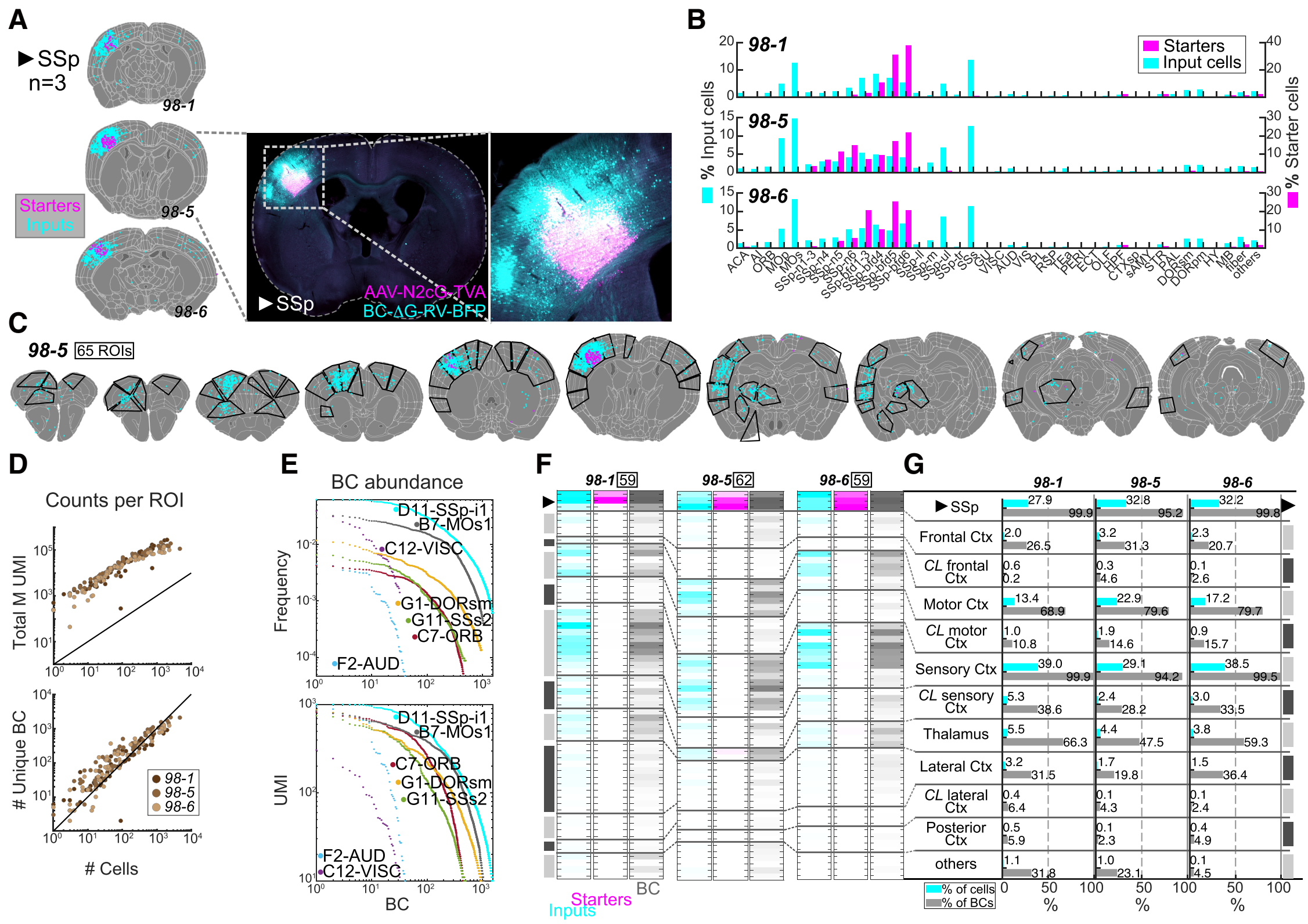

Figure 3. ROInet-seq for the primary somatosensory cortex barrel field

(A) Monosynaptic input tracing to the mouse somatosensory cortex (SSp) in three mice (animal IDs 98-1, 98-5, and 98-6) showing coronal sections containing the SSp start site. Note: after atlas alignment and cell registration (left), magenta represents starter cells (AAV+RV+).

(B) Quantification of starter and input cells after atlas registration, per experiment.

(C) Example coronal sections of alignment and microdissection strategy (ROIs encircled), along the anterior-posterior axis.

(D) For every ROI, counts of unique BCs and BC UMIs (sequencing) vs. automated cell counts (imaging).

(E) BCs abundance frequency (as quantified by molecule counts, UMIs), shown for seven ROIs.

(F) Per-ROI detection of input cells (cyan), starter cells (magenta), and BCs (gray; 98-1, 942; 98-5, 2,764; and 98-6, 1,381), in experiments 98-1, 98-5, and 98-6, normalized per column. ROIs are arranged and grouped by broad regional category; number of valid ROIs indicated next to experimental ID.

(G) Percentage of cells (cyan) and barcodes (gray) detected in the full brain, per the region's higher-order category.

~70% and 80% of all unique BCs, thalamic areas ~48%–66%, and contralateral somatosensory regions ~30% of BCs. Outside of this "core" of SSp-input regions (Figures 3F and 3G), frontal cortex areas, and lateral cortical association areas (including auditory, visceral, and entorhinal) also each contained ~20%–30% of BCs (Figure 3G).

Compared with fluorescent cell counts alone, our per-ROI BC quantifications gave striking additional insights into SSp input network architecture. For example, outside the SSp start site, other ipsilateral somatosensory areas were most dense in input cells, making up ~30% of all fluorescently labeled input cells (Figure 3G). At the same time, these somatosensory areas also contained close to 100% of all BCs detected, meaning that the labeled neurons in these regions connected to almost all SSp starter neurons. In another example, thalamic regions together accounted for only ~4.5% of fluorescently labeled input cells, but BC detection suggested that these cells connected to ~57% of all SSp starter cells, suggesting substantial synaptic input divergence from thalamus. Together, ROInet-seq uniquely revealed qualitative regional differences in input connectivity that is missed in transsynaptic viral connectivity tracing with fluorescent labels alone.

Finally, to move from BC detection to network mapping, we removed BCs at risk of multi-cell infection due to limits in library complexity and uniformity. We detected 941–2,763 unique BCs in the SSp start site ROIs alone and therefore expected that >20% of BCs could infect more than one neuron (Figures 1C and 1D, library 17.2). Since this would greatly hamper downstream interpretation of individual networks, we removed all BCs with frequencies $>10^{-4}$ in either all three mice, or a deeply sequenced *in vitro* library (Figure 4C). This step disproportionally removed BCs detected to label large networks more so than smaller networks (Figure 4D), suggesting that more abundant BCs may have indeed infected multiple neurons in the start site that would then erroneously resemble large networks. The final pool of robustly detected, and unique BCs we analyzed labeled 627–2,057 input networks that mostly spanned 5 or more ROIs, in all three experiments (Figure 4E). In line with recent results describing brain-wide inputs to single, electroporated SSp barrel field neurons,[18] ROInet-seq thus uniquely revealed

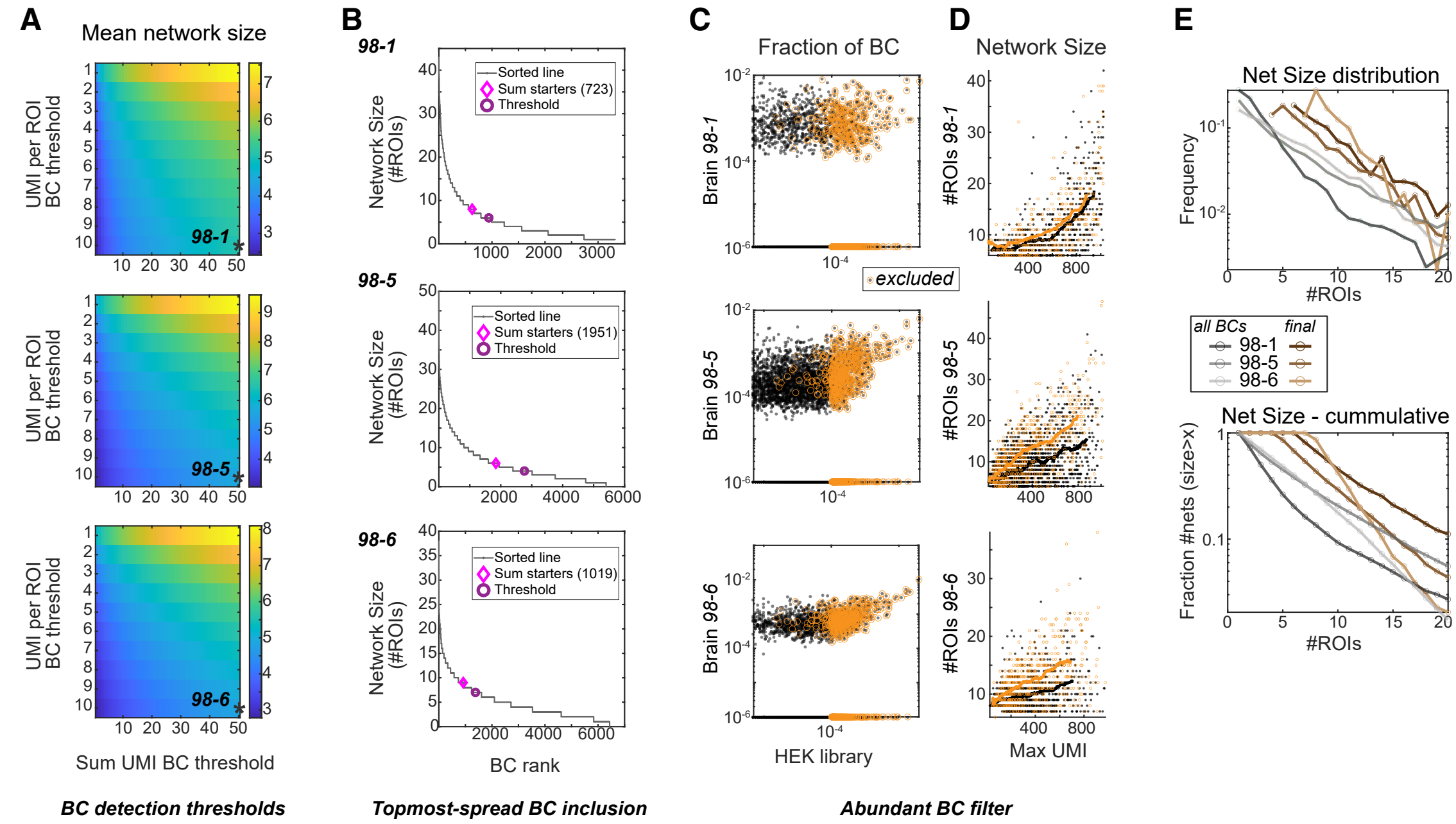

Figure 4. From ROInet-seq barcode detection to single-cell input networks

(A) Simulation of average network size by BC detection thresholds (UMIs). UMI per ROI locally included BCs detected 1–10 UMIs (10 UMIs in our analysis), and sum UMI globally excluded BCs detected 1–50 UMIs (50 UMIs in our analysis).

(B) BC ranking by network size after BC thresholds. Marks along the sorted line indicate number of counted starter cells, and the 1.5× starter cell threshold used to select the topmost widely spread BCs for network analysis (see STAR Methods).

(C) Unique BCs detected at high abundances *in vivo* (animal IDs: 98-1, 98-5, and 98-6) or *in vitro* were excluded for network analysis due to the risk of multiple infections in the start site. Black, all BCs; orange, abundant, discarded BCs. Numbers refer to BCs detected in the relevant experiment.

(D) Network size distribution before and after removal of highly abundant BCs (orange, as in C); shown as quantification of unique BCs (*x* axis) detected in # of ROIs (*y* axis). Lines indicate BCs fit before (orange) and after (black) removal.

(E) Network size distribution before BC threshold steps (gray lines) and in final dataset as absolute (top) or cumulative (bottom).

widespread patterns of multi-region inputs, but to hundreds of single SSp starter neurons per experiment.

We next examined individual neurons' networks in detail. We plotted the detection of every individual BC in each ROI and visualized these SSp input networks together with BC molecule copy numbers (UMIs) (Figure 5A and S5). Globally, we observed that even though BCs described co-inputs from multiple ROIs, inputs from one region quantitatively dominated each network (Figures 5B and S6). Most commonly, BCs were dominantly detected via somatosensory inputs and even locally in SSp start site ROIs. Dominant distal inputs came mainly from the motor cortex and the thalamus. These quantiative differences in distal input network architecture may reflect the starter neurons' distinct functions. In retrograde RV tracing, single electroporated SSp-bfd neurons that were active in movement (whisker deflection) received dominant inputs from thalamus, while non-movement associated SSp-bfd neurons were more dominantly innervated by the motor cortex.[18]

We next examined pairwise shared BCs (Figures 5C, S6, and S7) and found that several brain regions, and even functionally distinct areas, often co-input to single SSp starter cells. For example, over all experiments, 68%–75% of BCs described co-inputs of somatosensory and motor regions, 41%–63% were co-inputs of thalamus and somatosensory regions, and 35%–44% were co-inputs from thalamus and motor cortex. Further, we found that inputs from contralateral cortex regions almost always overlapped with their respective ipsilateral regions. For example, contralateral somatosensory regions accounted for 24%–37% of all BCs, and the same fraction of all network BCs described co-inputs of ipsilateral and contralateral somatosensory cortex (Figure 5C). The high frequency of these co-inputs may be unsurprising, as these regions also gave most overall inputs to the SSp start site. However, compared with their frequencies, several other co-input motifs were overrepresented (Figure 5C): For example, contralateral motor cortex consistuted only 10–13% of SSp inputs, and 10%–12% of all SSp inputs came from both ipsi- and contralateral motor areas; similar overrepresentation was observed for bilateral co-inputs from lateral cortical areas. Thus, our analysis revealed overrepresented motifs of synaptic convergence of ipsilateral and contralateral cortical regions with shared function to single SSp neurons, a network feature that was reproduced independent of stringent BC inclusion filters (Figure S6). Together, ROInet-seq uniquely detected and resolved higher-order input subnetworks of individual neurons from multiple remote sites, a connectivity property that is exclusively obtained only when single-cell resolution and brain-wide throughput are achieved in parallel.

To overcome the limited spatial resolution of ROInet-seq, we next applied BC-ΔG-RV for connectivity mapping on the commercial sequencing-based ST platforms Visium ST (10×

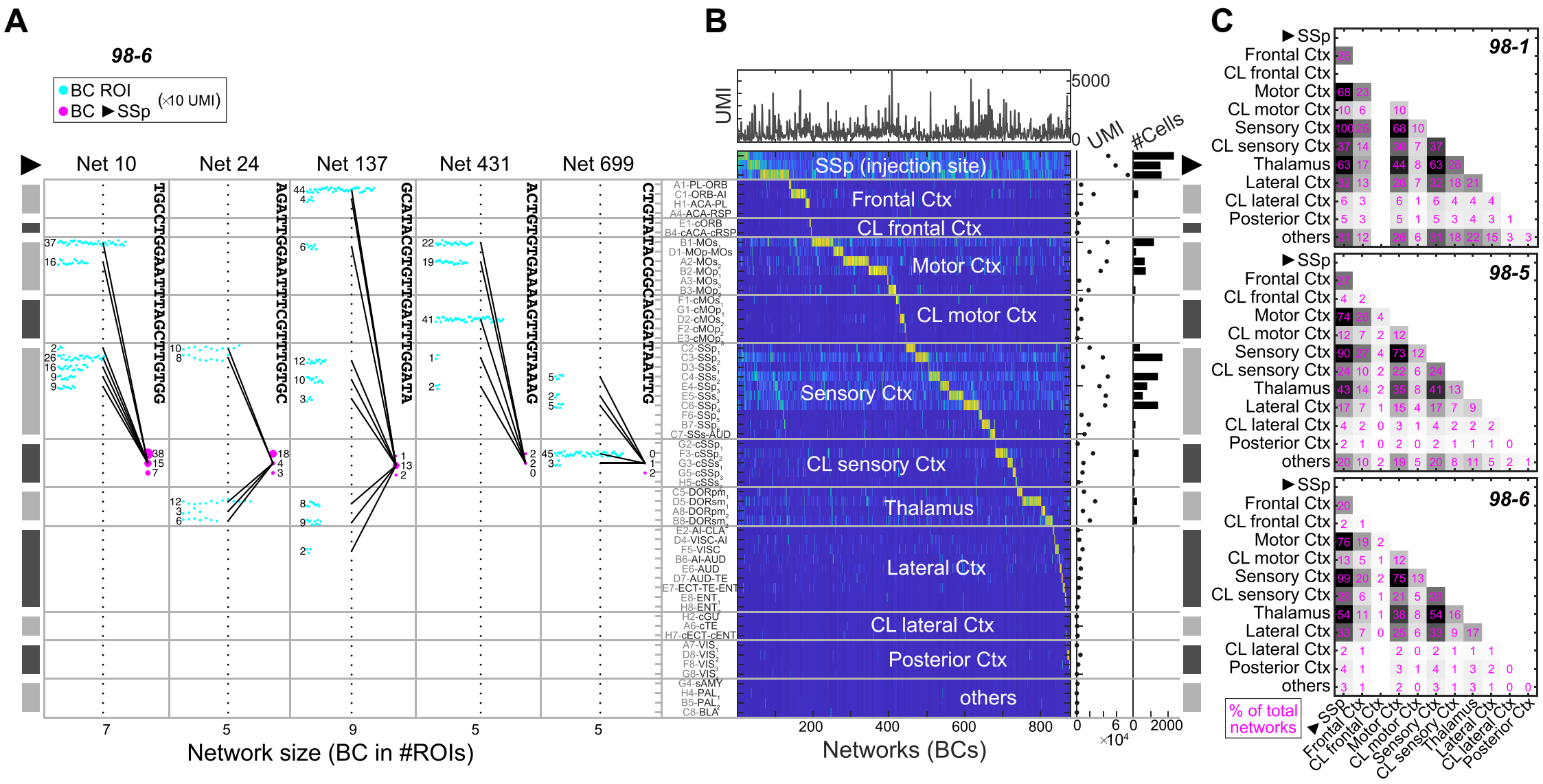

Figure 5. Quantification and convergence in SSp single-neuron input networks

(A) Brain-wide input networks to five single SSp start site neurons in brain 98-6 at ROI resolution, visualized as dots, where each cyan dot represents detection of 10 unique network BC copies (UMI) in input ROIs, and magenta blob size represents unique BC copies in SSp start site.

(B) Single-cell networks across 55 ROIs in brain 98-1, ordered by dominantly detected ROI. Colormap represents network BC copies (UMI) (blue, low; yellow, high).

(C) Convergent regional inputs to single SSp start site neurons in brains 98-1, 98-5, and 98-6. Co-detection of network BCs between 12 regions (as annotated in B), in % overlap of all detected network BCs. Greyscale represents minimum to maximum values standardized per ROI (excluding the diagonal).

Genomics) (Figure 6A) and Sterero-seq (STOmics) (Figure 7A). Given these platforms' limits in throughput and scale, we chose the hippocampus CA1 as start site, as most of its inputs come from within the hippocampal formation (CA1 and CA3). We injected BC-ΔG-RV in the CA1 (unilateral) and collected 10 μm fresh-frozen cryosections to the capture areas. In Visium, a chip of four 6.5 × 6.5 mm capture areas accommodated 16 coronal sections spanning the anterior-posterior axis of the hippocampus at 100 μm spacing (Figure 6A). In Stereo-seq, three chips with a capture area of 10 × 10 mm accommodated 27 coronal sections spanning the anterior-posterior axis of the hippocampus at 50 μm spacing (Figure 7A). For both platforms, we followed the standard protocol to generate transcriptome-wide cDNA libraries with spatial indices. For Visium, we additionally amplified the open reading frames (ORFs) of AAV-optimized glycoprotein (*oG*) and RV-*M* gene (flanked in its 3′ UTR by the BC sequence) (Figure S8). The final sequencing library allowed us to detect transcriptome-wide gene expression, network BCs, and viral infection, spatially resolved to capture spots that, in Visium, contained several cells (diameter 55 μm, center-to-center 110 μm) and in Stereo-seq, were of subcellular resolution (spot size ∼0.5 μm). Neither platforms allowed for pre-assay imaging of native fluorescence, so viral infection was confirmed post hoc using *in situ* hybridization (osmFISH)[19] against RV *M* gene on adjacent sections collected to separate slides (Figure 6B). In both assays, we detected elevated expression of inflammatory genes, such as *Cxcl13* and *Lcn2* (Figure S8), in the section most focal to the injection site. In Visium ST, this section also featured an unexpectedly low number of BCs (Figure 6D), together hinting at tissue damage in the start site.

Clustering of Stereo-seq spots confirmed microglial and macrophageal signatures among RV-transcript+ spots (Figure S9). As expected, RV expression was largely restricted to spots with CA1 pyramidal molecular signatures, but spots with high expression of RV-transcripts lacked distinct expression profiles of neuronal subtypes (Figure S9). The stereotypic cytoarchitecture of the hippocampus still allowed their confident mapping to the CA1 and key highly expressed interneuron markers (e.g., *Sst* and *Pvalb*) were confidently detected.

In a total sampling area spanning 18,177 capture spots, Visium ST detected 629 BCs distributed over 1,848 spots. Most BCs were detected in 5 spots or less, and each BC spot commonly featured 1 or 2 BCs (Figure 6C). BCs were typically restricted within <200 μm anterior-posterior axis, although 13 BCs were detected across 1500 μm sampled (Figure 6D). The most abundantly detected BCs in the dataset, representing the best-connected starter neurons, typically also had the widest anterior-posterior spread, while less-detected BCs, representing less-connected starter neurons, often received inputs from a single site in the CA1 or CA3 that was typically anterior to the start site (Figures 6E–6G and S8). These findings suggest that CA1 neurons can exhibit substantially distinct input network architectures—from single localized CA3 inputs to remarkably widespread inter-CA1 inputs spanning large anterior-posterior distances (Figures 6F–6H and S8).

In one drawback, the spot resolution of Visium ST could not distinguish whether the detected BC spread stemmed from actual network cells, their projections (X-Y-Z), or even just diffusion of transcripts on the array (X-Y). When we observed similar spread of RV transcripts on the Stereo-seq STOmics array, the

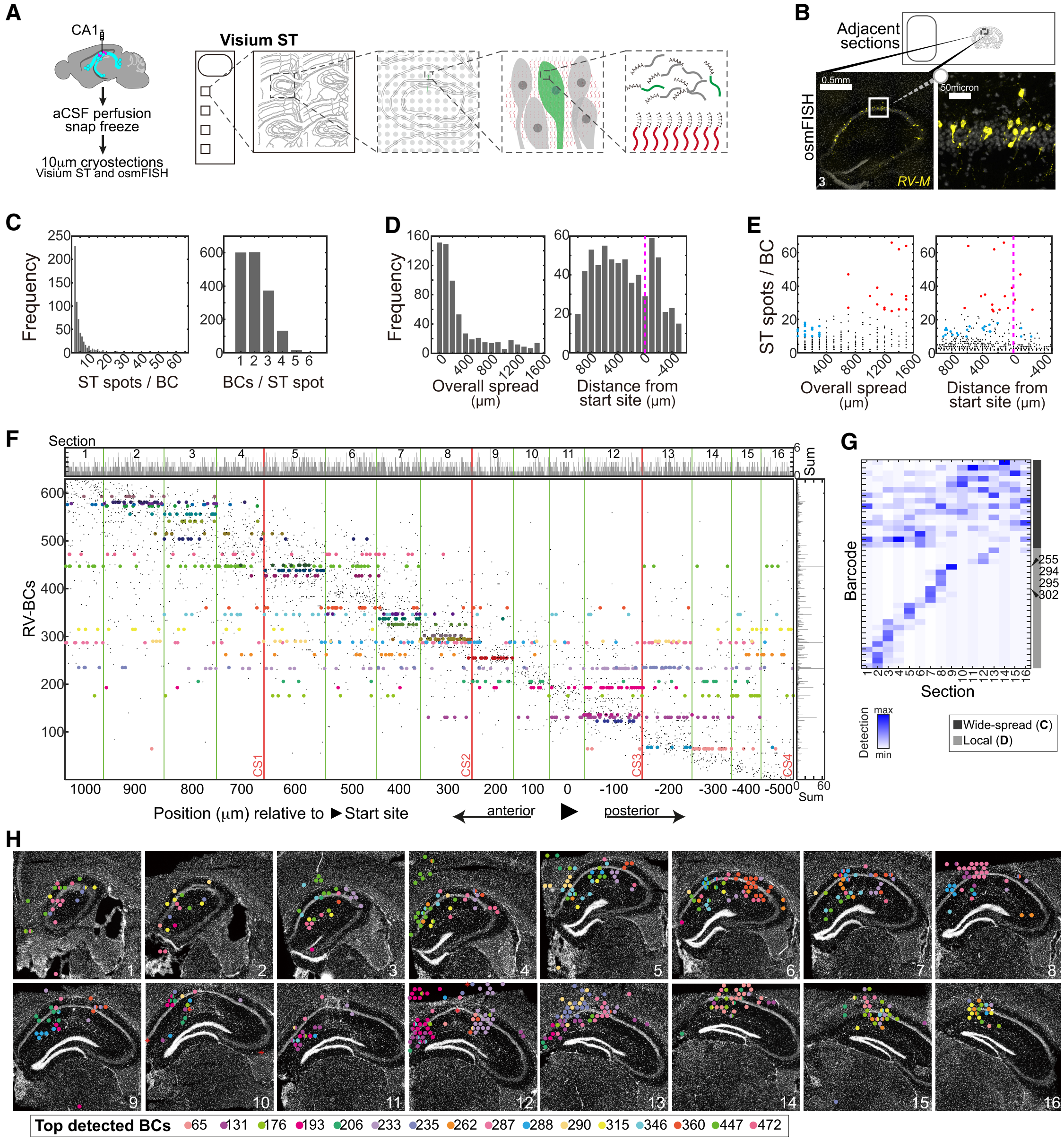

Figure 6. Visium spatial transcriptomics for increased resolution of local network reconstruction

(A) Experimental outline of Visium ST experiment: collection of 16 coronal cryosections of the hippocampus, spanning the anterior-posterior axis at 100 mm spacing. Per ST capture area, 4 sections were collected. After cDNA library construction, sequences of interests (AAV and RV) were enriched and sequenced.

(B) Validation of RV infection in sections consecutive to the Visium ST sampling, using single molecule FISH (osmFISH) against the RV matrix gene (*M*). Example and zoom in on osmFISH section adjacent to Visium ST section 3.

(C) Detection of RV barcodes (BCs) in Visium ST: left, detection of barcodes in ST spots, where most BCs were restricted to 5 spots; right, number of BCs per spot, where most RV+ spots contained one or two barcodes.

(D) Left: distribution of total spreading distance of barcodes; right: spreading distance relative to the start site.

(E) Left: scatterplot of quantified barcode detection, by total spreading distance; right: scatterplot of quantified barcode detection, by relative position to the start site (average per BC). Every spot represents one barcode; red labels the top 16 most abundantly detected BCs; and blue denotes 22 of the most locally restricted BCs.

(F) Distribution map of all 629 detected barcodes (rows) across all RV+ 1,848 Visium ST spots (columns), spanning16 anterior-posterior sections.

(G) Detection of 16 top detected and 22 locally mapping BCs, across 16 anterior-posterior sections. Blue, high; white, low.

(H) Spatially resolved detection of the top 16 abundant barcodes in 16 anterior-posterior hippocampus sections of Visium ST; white, nuclei counterstain (DAPI).

CellPress
OPEN ACCESS

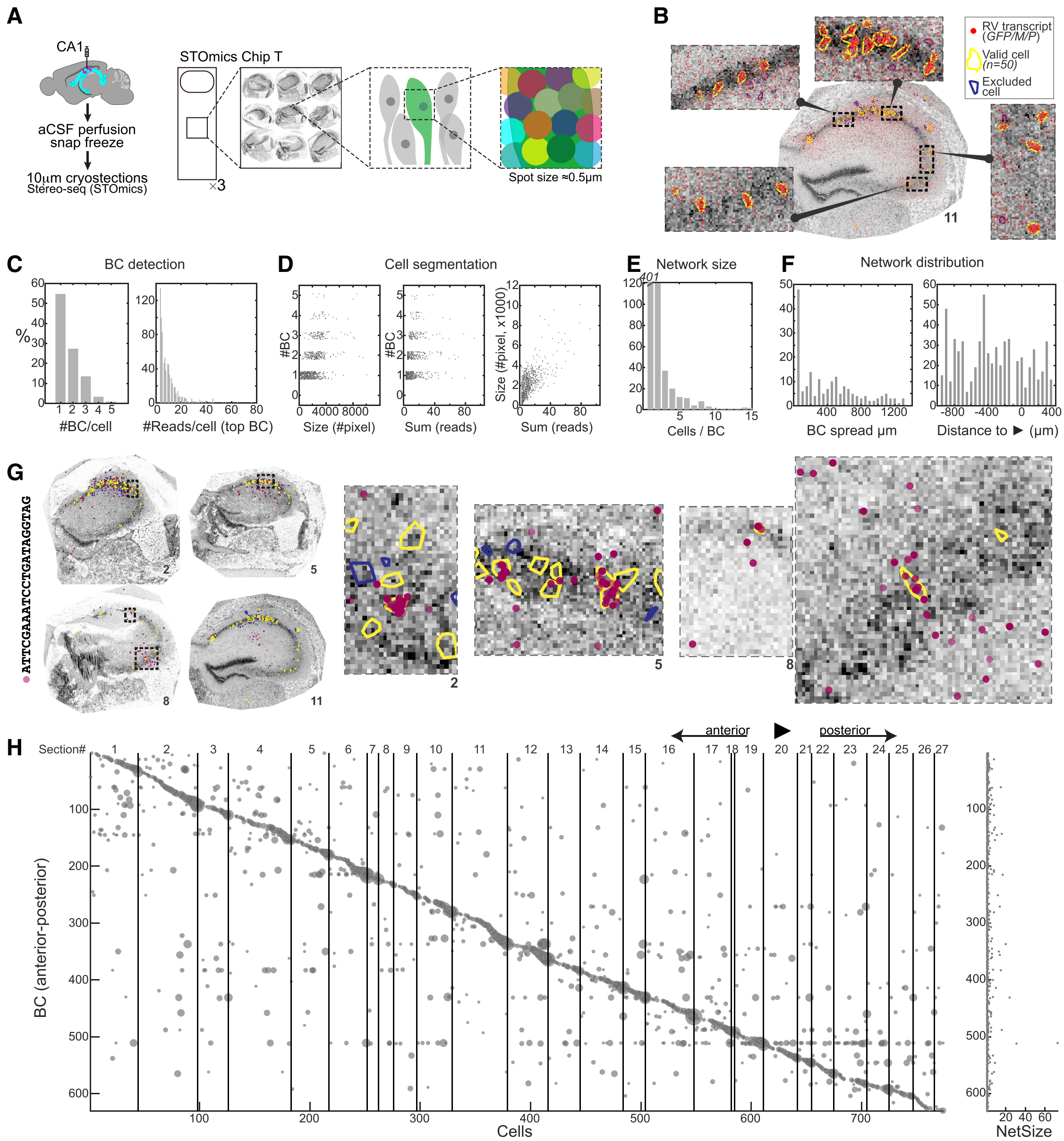

Figure 7. STOmics Stereo-seq for hippocampus input network resolution

(A) Workflow for input network tracing in the hippocampus CA1 using the STOmics Stereo-seq platform. We performed BC-ΔG-RV tracing after unilateral injection to the CA1 and collected 27 10-µm fresh-frozen cryosections to 3 STOmics slides (10 × 10 mm).

(B) Network cell segmentation example in section 11 based on RV transcript clusters, detecting 50 putative single network cell soma (yellow), with zoom ins on CA1 (top) and CA3 (bottom and right). Red dots, expression levels of RV transcripts *GFP*, *M*, and *P* (bin 1); yellow outlines, segmented network cells; blue outlines, segmented cells below size or detection threshold.

(C) Distribution of per-cell BC detection (left) and the number of reads of the most-expressed BC per cell (median = 7 reads).

(D) Number of BCs detected per segmented putative network cell as a function of cell size (left) and RV reads (*GFP*, *M*, and *P*) in the cell (middle) and network cell size as a function of RV reads (right). Most segmented network cells contained a single BC; both BC detection and total RV reads correlated with cell size.

(E) Network size distribution, as number of cells per unique BC.

(F) Anterior-posterior distribution of BCs in absolute distance (left) and relative to the start site (right).

(G) Example of a single input network, visualized by BC detection (purple dots; BC sequence, left), in 4 sections. BCs detected outside the bounds of putative network cell somas (yellow outlines) are disregarded. Blue outlines depict segmented cells below size or detection threshold.

(H) Anterior-posterior distribution and detection of individual BCs in all network cells, in section order. Blob size represents the number of BC reads.

assay's subcellular spot resolution allowed for data-led cell segmentation: reasonably-sized spatial spot clusters expressing RV-transcripts and BCs were interpreted as single labeled network cells (Figure 7B, STAR Methods), and we analyzed only BCs detected within the bounds of these likely cell bodies (Figures 7B and 7C). Spurious detection of RV-transcripts (including BCs) outside of spatial clusters was disregarded. Across 27 sections, we detected 774 network cells containing, in total, 631 unique BCs. In line with scRNA-seq (Figures 1F and 1G), most network cells contained a single BC (Figure 7C). Cells with multiple BCs tended to be larger, reserving the possibility that some multi-BC cells may be unsegmented neighbor cells (Figures 7B and 7D). Most BCs were detected in only one cell, but the method also detected 230 multicell (>1) networks, with 53 spanning 5 or more cells (Figures 7E and S10). Networks were mostly restricted to ~400 μm in anterior-posterior distribution, with a slight bias to network labeling anterior to the start site (Figure 7F); as exemplified also in Figure 7G. RV genes were the most highly detected genes in network cells. In a subset of network cells, we also detected expression of transcripts that indicated their cell identity (Figures S9and S10). For example, glutamatergic markers labeled ~142 of the network cells, and most expressed pyramidal marker *Neurod6*, with 124 positive for CA1-2 marker *Spink8*, and 38 expressing CA3 marker *Nptx1*, while only ~20 cells expressed a general GABAergic signature (*Gad2*, *Gad1*, and *Slc32a1*). More highly expressed neuropeptides *Sst* and *Npy,* marking GABAergic interneuron subpopulations, were better detected, in ~40 cells each.

Together, we demonstrated the application of single-cell input network tracing at spatial and single-cell resolution on two commercially available ST platforms. In the hippocampus, this revealed both locally restricted and wide-spread input network motives, and the platforms' spatial resolution allowed to combine network information with cell location. In brain regions with complex and intermingled cellular diversity, these available methods already have the potential to reveal local input network architecture of molecularly defined cell types.

DISCUSSION

To systematically describe neuronal network architecture at single-cell resolution, we developed ROInet-seq, a scalable approach that combines monosynaptic input tracing with BC-ΔG-RV. Each starter cell receives a unique BC, and brain ROIs that contain network cells are microdissected and sequenced in bulk. Because BCs are unique per network, input networks to single cells can be reconstructed, at ROI resolution. ROInet-seq integrates seamlessly with standard histology, requires only simple molecular processing and shallow sequencing, and—using a highly complex, uniform viral library—resolves hundreds of input networks in parallel within a single experiment.

Applied to the SSp-bfd, our results are coherent over independent experiments and consistent with previous characterization of bulk input organization.[20,21] Beyond imaging-based quantification, sequencing revealed diverse connectivity patterns. For instance, although thalamic neurons accounted for only ~4.5% of imaged presynaptic cells, they provided input to ~57% of starter neurons, pointing at thalamic input divergence. Compared with *in situ* sequencing studies,[15,16] ROInet-seq reconstructed larger brain-spanning networks and uncovered extensive convergence from multiple distant sites onto single SSp neurons. This included enriched motifs of simultaneous ipsilateral and contralateral inputs from functionally overlapping cortical regions. Incorporating UMIs enabled molecular counting of BC copies, providing quantitative measures such as regional input dominance. While variability in detection limits exact quantification, the linear scaling between UMI counts and imaged cells supports this interpretation.

Notably, several key findings were independently confirmed by Inácio et al.,[18] who combined two-photon calcium imaging in SSp-bfd with monosynaptic tracing from a single neuron per brain. First, the authors described expected massive local connectivity from within the SSp-bfd (~80% of presynaptic cells), reflected in our findings via a large portion of BCs dominating within the SSp. Further, the authors described wide-spread, converging inputs from 6 to 7 larger brain regions, similar to our observed network sizes. Finally, Inácio et al. observed quantitative input dominance depending on neuronal functional state (i.e., movement-correlated or not). It will be of great interest to test whether the quantitative input differences we observed in ROInet-seq may reflect similar functional specializations, and perhaps even network plasticity, of the traced postsynaptic neurons. These parallels suggest that ROInet-seq not only recapitulates quantitative and qualitative connectivity features but also extends them to hundreds of neurons simultaneously, providing robust and sensitive network maps at unmatched throughput. Importantly, the agreement with single-cell electroporation studies underscores the reliability of our approach.

We further applied ROInet-seq in combination with commercial ST (Visium ST and STOmics Stereo-seq). In hippocampus, these assays enabled anterior-posterior sampling and higher spatial resolution of local BC-ΔG-RV networks. STOmics achieved single-cell resolution and confirmed *in vivo* infection parameters also observed in scRNA-seq, such as multi-BC infection. However, both fresh-tissue ST and scRNA-seq suffered from sparse recovery of network cells—due to cell loss during dissociation, as reported previously, [15] or limited spatial scale—and reduced transcriptome quality of RV-infected cells. Consequently, detailed cell-type annotation of network members was limited. In contrast, ROInet-seq provided gapless, brain-wide coverage of inputs to SSp, albeit without capturing transcriptomes of individual presynaptic neurons. Therefore, we maintain that on the long run, cell-resolution spatial platforms compatible with sampling fixed, intact tissue, and with ever-larger sampling area are needed to overcome remaining issues in network cell recovery and transcriptomic fidelity of RV-infected neurons.

The remaining trade-offs between single-cell resolution and gapless sampling in BC-ΔG-RV also currently restrict the possibility to describe network motives such as reciprocal neuronal connectivity (A↔B). Given a uniform BC-ΔG-RV library and gapless spatial sequencing within the start site, however, the method would allow detection of reciprocal connections between starter cells, when the same combination of unique BCs is detected in two starter cells. In practice, however, reciprocal connections are sparse compared with overall connectivity[22]

and synaptic transfer efficiencies of RV would have to be near optimal (discussed in the following text) to detect them. The detection of such motives could also be aided by genetic targeting of subpopulations (e.g., layer-specific) with known interconnectivity or by reporter expression conditioned on the mixing of two BC-ΔG-RV library versions.

Monosynaptic rabies tracing has already revealed circuit differences linked to sex dimorphism[23] and drug-induced circuit plasticity.[24,25] BC-ΔG-RV tracing with ROInet-seq can be an accessible, high-throughput method for single-cell resolved connectivity mapping, well-suited for comparative and functional neuroscience. However, compared with current EM-based reconstructions,[22] monosynaptic input tracing underestimates overall connectivity, both due to inefficient retrograde synaptic transfer, especially to distal inputs, and due to cytotoxicity.[6,26] Yet, recent advances, such as double-deletion B19 variants enabling long-term survival[27] and improved N2c substrains with higher transsynaptic efficiency, labeling distance and viability[26,28] suggest that continued improvements in viral design will further enhance the sensitivity and functional relevance of BC-ΔG-RV. Additionally, perhaps counterintuitively, it was argued that since transsynaptic transfer efficiency is related to connectivity strength via the number of synaptic contacts and size of postsynaptic density, only transfer efficiencies far below 100% would make the method sensitive to functional connection strength.[29]

Even while rabies-based network reconstruction might be incomplete, ROInet-seq sub-sampled networks of hundreds of cells in parallel and could give valuable insights to regional network motives via its scalability as long as the virus had no inherent bias in synaptic transfer. In this study, scRNA-seq and STOmics Stereo-seq detected substantially more RV-labeled excitatory neurons. This is largely expected, given the dominance of excitatory neurons[30,31] in isocortex and CA1. Here, we did not formally assess any potential remaining bias, not least since confident cell-type assignment was limited by sequencing depth and the dominant expression of RV-transcripts. Elsewhere, monosynaptic tracing with RV reported numbers[17,32] similar to EM-reconstruction,[22] and the efficiency of monosynaptic rabies virus transfer was also explicitly shown not to be biased between several distinct molecular cell types.[29]

Additional efforts linking connectivity and function would be feasible using BC-ΔG-RV with ROInet-seq. For example, it would be exciting to introduce functional recordings prior to mapping the recorded cells' brain-wide input networks for dozens of cells in parallel. This could be achieved when two-photon calcium imaging under a certain task precedes BC-ΔG-RV tracing with that same area as the start site. Maps of the recorded cells would need to be co-registered to ST (e.g., with STOmics Stereo-seq), to relate calcium traces to BCs at single-cell resolution in the recorded start site. Though a practical hurdle, this would also add valuable transcriptome information of the recorded cells and could provide exciting scale and detail to network motifs, such as those described for direction sensitivity in the V1[32]. In the rest of the brain, a bulk-level assay such as ROInet-seq could then provide sufficient regional resolution and quantitative insights to relate cell activity to distal inputs, as was beautifully demonstrated for differential connectivity of single neurons with distinct functions in a motor task.[18] Together, brain-wide monosynaptic tracing with BC-ΔG-RV is a versatile tool in mapping networks that could continue to be adapted to a range of applications and functional settings, and ROInet-seq provides the throughput and price tag to enable comparative sampling.

Limitations of the study

Like all approaches for the reconstruction of synaptic networks, ROInet-seq involves several trade-offs. First, although UMI-based molecular counting provided valuable measures of regional input dominance, variability in detection prevents strictly quantitative estimates of connectivity strength. Second, the observed convergence and divergence patterns (e.g., thalamic inputs) cannot be fully disentangled from potential biases introduced by BC detection or viral transfer efficiency. Third, the method sacrifices transcriptomic and refined spatial information of presynaptic neurons in exchange for brain-wide coverage and throughput, leaving the connectome-transcriptome link unresolved. Given the immense diversity of neuronal cells in the brain readily characterized by single-cell and ST tools,[33–37] the transcriptome-connectome link will be critical to implement. Fourth, rabies-based tracing itself remains limited by incomplete retrograde transfer and cytotoxicity, which constrain the recovery and fidelity of network neurons. Finally, the bulk ROI-based design lacks the single-cell anatomical precision achievable with ST or *in situ* methods, though these remain hampered by cost, tissue compatibility, and scale. These remaining limitations in not only rabies virus transsynaptic efficiency but also in resolution and coverage or scale of downstream assays also mean that no current implementation of BC-ΔG-RV tracing can describe network motives such as reciprocal connectivity.

Despite these limitations, our results demonstrate that BC-ΔG-RV with ROInet-seq provides robust, reproducible maps of neuronal input networks at unprecedented throughput. Future improvements in viral design, integration with transcriptomic profiling, and advances in ST, including emerging sequencing-based ST tools with ever-larger sampling areas and compatible with fixed tissue, are likely to further close the gap between connectomics resolution, molecular identity, and functional interpretation—bringing brain-wide single-cell network reconstruction within reach.

RESOURCE AVAILABILITY

Lead contact

Requests for further information and resources should be directed to and will be fulfilled by the lead contact, Amit Zeisel (amit.zeisel@technion.ac.il).

Materials availability

There are restrictions to the availability of barcoded libraries (plasmid and rabies virus) because of the lack of an external centralized repository for its distribution and our need to maintain the stock. We are glad to share the libraries with reasonable compensation from the requestor for its processing and shipping.

Data and code availability

- Spatial transcriptomics data using the 10× Genomics Visium platform and scRNA-seq data using the 10× Genomics Chromium as processed

expression tables with annotations and metadata have been deposited at Figshare (https://doi.org/10.6084/m9.figshare.29952065). Spatial transcriptomics data using the STOmics Stereo-seq platform have been deposited at Figshare at a resolution of bin10 (https://doi.org/10.6084/m9.figshare.32204544). All are publicly available as of the date of publication. Source Data for ROInet-seq (BC count tables and metadata) are provided in this paper (Table S1).

- All original code has been deposited at Figshare (https://doi.org/10.6084/m9.figshare.29952065) and in GitHub (https://github.com/zeiselamit/ROInetseq) and is publicly available at as of the date of publication.
- Any additional information required to reanalyze the data reported in this paper is available from the lead contact upon request.

ACKNOWLEDGMENTS

This research was supported by the European Research Council (TYPEWIRE-852786 to A.Z.) and Human Frontiers Science Program (CDA-0039/2019-C to A.Z.). We thank the Azrieli Technion Genomics Center and the FACS unit of the Technion LS&E Infrastructure Center for technical assistance and former Zeisel lab member Etay Aloni for critical discussion.

AUTHOR CONTRIBUTIONS

Z.L., H.H., and A.Z. designed the study and planned experiments. Z.L. performed *in vitro* validation, viral injections, histology and ROI-microdissections, osmFISH, and Visium ST and Stereo-seq analyses. O.O. performed ROInet-seq, *in vitro* validation, and Visium ST and Stereo-seq analyses. T.T., B.G., and C.T. designed and performed RV library preparations and optimizations. H.S. constructed and produced RV plasmid libraries. H.H. performed scRNA-seq. Z.L. and A.Z. analyzed *in vitro* validation data and ROInet-seq data. A.Z. and H.H. analyzed scRNA-seq data. M.T. analyzed Visium ST data. H.H., A.Z., and Z.L. interpreted all data. H.H. assembled figures and wrote the paper, with help from A.Z. and input from all authors.

DECLARATION OF INTERESTS

The authors declare no competing interests.

STAR★METHODS

Detailed methods are provided in the online version of this paper and include the following:

- KEY RESOURCES TABLE
- EXPERIMENTAL MODEL AND STUDY PARTICIPANT DETAILS
- METHOD DETAILS
  - Plasmid library generation ΔG-SAD-B19
  - Barcoded ΔG-SAD-B19 rabies virus generation
  - BR-16.1
  - BR-16.2/BR-16.3
  - *BR-16.4*/*BR-16.5*/*BR-16.6* and *BR-17.1*
  - Quality control barcoded ΔG-SAD-B19 *in vitro*
  - Viral injections
  - scRNA-seq of barcoded δg-SAD-B19 injected brains
  - Histology and ROInet-seq
  - Stereo-seq cell-resolved spatial transcriptomics networks reconstruction
  - Visium ST networks sequencing and analysis
  - osmFISH
- QUANTIFICATION AND STATISTICAL ANALYSIS
  - ROInet-seq data processing and analysis
  - Visium ST networks sequencing and analysis
  - Stereo-seq cell-resolved spatial transcriptomics networks reconstruction

SUPPLEMENTAL INFORMATION

Supplemental information can be found online at https://doi.org/10.1016/j.crmeth.2026.101512.

Received: August 21, 2025
Revised: January 23, 2026
Accepted: June 9, 2026
Published: July 7, 2026

REFERENCES

1. Mahalingam, G., Torres, R., Kapner, D., Trautman, E.T., Fliss, T., Seshamani, S., Perlman, E., Young, R., Kinn, S., Buchanan, J., et al. (2022). A scalable and modular automated pipeline for stitching of large electron microscopy datasets. eLife *11*, e76534. https://doi.org/10.7554/eLife.76534.
2. Turner, N.L., Macrina, T., Bae, J.A., Yang, R., Wilson, A.M., Schneider-Mizell, C., Lee, K., Lu, R., Wu, J., Bodor, A.L., et al. (2022). Reconstruction of neocortex: Organelles, compartments, cells, circuits, and activity. Cell *185*, 1082–1100.e24. https://doi.org/10.1016/j.cell.2022.01.023.
3. Yin, W., Brittain, D., Borseth, J., Scott, M.E., Williams, D., Perkins, J., Own, C.S., Murfitt, M., Torres, R.M., Kapner, D., et al. (2020). A petascale automated imaging pipeline for mapping neuronal circuits with high-throughput transmission electron microscopy. Nat. Commun. *11*, 4949. https://doi.org/10.1038/s41467-020-18659-3.
4. Michalska, J.M., Lyudchik, J., Velicky, P., Štefaničková, H., Watson, J.F., Cenameri, A., Sommer, C., Amberg, N., Venturino, A., Roessler, K., et al. (2023). Imaging brain tissue architecture across millimeter to nanometer scales. Nat. Biotechnol. *42*, 1051–1064. https://doi.org/10.1038/s41587-023-01911-8.
5. Tavakoli, M.R., Lyudchik, J., Januszewski, M., Vistunou, V., Agudelo Dueñas, N., Vorlaufer, J., Sommer, C., Kreuzinger, C., Oliveira, B., Cenameri, A., et al. (2025). Light-microscopy-based connectomic reconstruction of mammalian brain tissue. Nature *642*, 398–410. https://doi.org/10.1038/s41586-025-08985-1.
6. Fuzik, J., Zeisel, A., Máté, Z., Calvigioni, D., Yanagawa, Y., Szabó, G., Linnarsson, S., and Harkany, T. (2016). Integration of electrophysiological recordings with single-cell RNA-seq data identifies neuronal subtypes. Nat. Biotechnol. *34*, 175–183. https://doi.org/10.1038/nbt.3443.
7. Oh, S.W., Harris, J.A., Ng, L., Winslow, B., Cain, N., Mihalas, S., Wang, Q., Lau, C., Kuan, L., Henry, A.M., et al. (2014). A mesoscale connectome of the mouse brain. Nature *508*, 207–214. https://doi.org/10.1038/nature13186.
8. Qiu, S., Hu, Y., Huang, Y., Gao, T., Wang, X., Wang, D., Ren, B., Shi, X., Chen, Y., Wang, X., et al. (2024). Whole-brain spatial organization of hippocampal single-neuron projectomes. Science *383*, eadj9198. https://doi.org/10.1126/science.adj9198.
9. Wickersham, I.R., Lyon, D.C., Barnard, R.J.O., Mori, T., Finke, S., Conzelmann, K.-K., Young, J.A.T., and Callaway, E.M. (2007). Monosynaptic Restriction of Transsynaptic Tracing from Single, Genetically Targeted Neurons. Neuron *53*, 639–647. https://doi.org/10.1016/j.neuron.2007.01.033.
10. Suzuki, T., Morimoto, N., Akaike, A., and Osakada, F. (2020). Multiplex Neural Circuit Tracing With G-Deleted Rabies Viral Vectors. Front. Neural Circ. *13*, 77. https://doi.org/10.3389/fncir.2019.00077.
11. Chen, X., Sun, Y.-C., Zhan, H., Kebschull, J.M., Fischer, S., Matho, K., Huang, Z.J., Gillis, J., and Zador, A.M. (2019). High-Throughput Mapping of Long-Range Neuronal Projection Using In Situ Sequencing. Cell *179*, 772–786.e19. https://doi.org/10.1016/j.cell.2019.09.023.
12. Kebschull, J.M., Garcia Da Silva, P., Reid, A.P., Peikon, I.D., Albeanu, D.F., and Zador, A.M. (2016). High-Throughput Mapping of Single-Neuron Projections by Sequencing of Barcoded RNA. Neuron *91*, 975–987. https://doi.org/10.1016/j.neuron.2016.07.036.
13. Clark, I.C., Gutiérrez-Vázquez, C., Wheeler, M.A., Li, Z., Rothhammer, V., Linnerbauer, M., Sanmarco, L.M., Guo, L., Blain, M., Zandee, S.E.J., et al. (2021). Barcoded viral tracing of single-cell interactions in central nervous system inflammation. Science *372*. https://doi.org/10.1126/science.abf1230.

CellPress
OPEN ACCESS

14. Saunders, A., Huang, K.W., Vondrak, C., Hughes, C., Smolyar, K., Sen, H., Philson, A.C., Nemesh, J., Wysoker, A., Kashin, S., et al. (2022). Ascertaining cells' synaptic connections and RNA expression simultaneously with barcoded rabies virus libraries. Nat. Commun. *13*, 6993. https://doi.org/10.1038/s41467-022-34334-1.

15. Zhang, A., Jin, L., Yao, S., Matsuyama, M., van Velthoven, C., Sullivan, H., Sun, N., Kellis, M., Tasic, B., Tasic, B., et al. (2023). Rabies virus-based barcoded neuroanatomy resolved by single-cell RNA and in situ sequencing. eLife *12*. https://doi.org/10.7554/eLife.87866.1.

16. Becalick, A., Blot, A., Strom, M., and Znamenskiy, P. (2025). Barcoded Rabies in Situ Connectomics for High-Throughput Reconstruction of Neural Circuits (Preprint at Cold Spring Harbor Laboratory). https://doi.org/10.1101/2025.07.16.665048.

17. Patiño, M., Lagos, W.N., Patne, N.S., Tasic, B., Zeng, H., and Callaway, E.M. (2022). Single-cell transcriptomic classification of rabies-infected cortical neurons. Proc. Natl. Acad. Sci. *119*, e2203677119. https://doi.org/10.1073/pnas.2203677119.

18. Inácio, A.R., Lam, K.C., Zhao, Y., Pereira, F., Gerfen, C.R., and Lee, S. (2025). Brain-wide presynaptic networks of functionally distinct cortical neurons. Nature *641*, 1–11. https://doi.org/10.1038/s41586-025-08631-w.

19. Codeluppi, S., Borm, L.E., Zeisel, A., La Manno, G., van Lunteren, J.A., Svensson, C.I., and Linnarsson, S. (2018). Spatial organization of the somatosensory cortex revealed by osmFISH. Nat. Methods *15*, 932–935. https://doi.org/10.1038/s41592-018-0175-z.

20. Feldmeyer, D. (2012). Excitatory neuronal connectivity in the barrel cortex. Front. Neuroanat. *6*, 24. https://doi.org/10.3389/fnana.2012.00024.

21. DeNardo, L.A., Berns, D.S., DeLoach, K., and Luo, L. (2015). Connectivity of mouse somatosensory and prefrontal cortex examined with trans-synaptic tracing. Nat. Neurosci. *18*, 1687–1697. https://doi.org/10.1038/nn.4131.

22. Bae, J.A., Baptiste, M., Baptiste, M.R., Bishop, C.A., Bodor, A.L., Brittain, D., Brooks, V., Buchanan, J., Bumbarger, D.J., Castro, M.A., et al. (2025). Functional connectomics spanning multiple areas of mouse visual cortex. Nature *640*, 435–447. https://doi.org/10.1038/s41586-025-08790-w.

23. Aloni, E., Tibi, M., Hochgerner, H., and Zeisel, A. (2023). Sexual dimorphism in synaptic inputs to the mouse amygdala and orbital cortex. Front. Neurosci. *17*, 1258284. https://doi.org/10.3389/fnins.2023.1258284.

24. Beier, K.T., Kim, C.K., Hoerbelt, P., Hung, L.W., Heifets, B.D., DeLoach, K.E., Mosca, T.J., Neuner, S., Deisseroth, K., Luo, L., and Malenka, R.C. (2017). Rabies screen reveals GPe control of cocaine-triggered plasticity. Nature *549*, 345–350. https://doi.org/10.1038/nature23888.

25. Jiang, Q., Shao, L.-X., Yao, S., Savalia, N.K., Gilbert, A.D., Davoudian, P.A., Nothnagel, J.D., Tian, G., Hung, T.S., Lai, H.M., et al. (2026). Psilocybin triggers an activity-dependent rewiring of large-scale cortical networks. Cell *189*, 659–675.e22, *0*. https://doi.org/10.1016/j.cell.2025.11.009.

26. Reardon, T.R., Murray, A.J., Turi, G.F., Wirblich, C., Croce, K.R., Schnell, M.J., Jessell, T.M., and Losonczy, A. (2016). Rabies Virus CVS-N2c ΔG Strain Enhances Retrograde Synaptic Transfer and Neuronal Viability. Neuron *89*, 711–724. https://doi.org/10.1016/j.neuron.2016.01.004.

27. Jin, L., Sullivan, H.A., Zhu, M., Lavin, T.K., Matsuyama, M., Fu, X., Lea, N.E., Xu, R., Hou, Y., Rutigliani, L., et al. (2024). Long-term labeling and imaging of synaptically connected neuronal networks in vivo using double-deletion-mutant rabies viruses. Nat. Neurosci. *27*, 373–383. https://doi.org/10.1038/s41593-023-01545-8.

28. Sumser, A., Joesch, M., Jonas, P., and Ben-Simon, Y. (2022). Fast, high-throughput production of improved rabies viral vectors for specific, efficient and versatile transsynaptic retrograde labeling. eLife *11*, e79848. https://doi.org/10.7554/eLife.79848.

29. Patiño, M., Lagos, W.N., Patne, N.S., Miyazaki, P.A., Bhamidipati, S.K., Collman, F., and Callaway, E.M. (2023). Postsynaptic cell type and synaptic distance do not determine efficiency of monosynaptic rabies virus spread measured at synaptic resolution. eLife *12*, e89297. https://doi.org/10.7554/eLife.89297.

30. Keller, D., Erö, C., and Markram, H. (2018). Cell Densities in the Mouse Brain: A Systematic Review. Front. Neuroanat. *12*. https://doi.org/10.3389/fnana.2018.00083.

31. Elabbady, L., Seshamani, S., Mu, S., Mahalingam, G., Schneider-Mizell, C.M., Bodor, A.L., Bae, J.A., Brittain, D., Buchanan, J., Bumbarger, D.J., et al. (2025). Perisomatic ultrastructure efficiently classifies cells in mouse cortex. Nature *640*, 478–486. https://doi.org/10.1038/s41586-024-07765-7.

32. Rossi, L.F., Harris, K.D., and Carandini, M. (2020). Spatial connectivity matches direction selectivity in visual cortex. Nature *588*, 648–652. https://doi.org/10.1038/s41586-020-2894-4.

33. Bakken, T.E., Jorstad, N.L., Hu, Q., Lake, B.B., Tian, W., Kalmbach, B.E., Crow, M., Hodge, R.D., Krienen, F.M., Sorensen, S.A., et al. (2021). Comparative cellular analysis of motor cortex in human, marmoset and mouse. Nat *598*, 111–119. https://doi.org/10.1038/s41586-021-03465-8.

34. Siletti, K., Hodge, R., Mossi Albiach, A., Lee, K.W., Ding, S.-L., Hu, L., Lönnerberg, P., Bakken, T., Casper, T., Clark, M., et al. (2023). Transcriptomic diversity of cell types across the adult human brain. Science *382*, eadd7046. https://doi.org/10.1126/science.add7046.

35. Yao, Z., van Velthoven, C.T.J., Nguyen, T.N., Goldy, J., Sedeno-Cortes, A.E., Baftizadeh, F., Bertagnolli, D., Casper, T., Chiang, M., Crichton, K., et al. (2021). A taxonomy of transcriptomic cell types across the isocortex and hippocampal formation. Cell *184*, 3222–3241.e26. https://doi.org/10.1016/j.cell.2021.04.021.

36. Zeisel, A., Muñoz-Manchado, A.B., Codeluppi, S., Lönnerberg, P., La Manno, G., Juréus, A., Marques, S., Munguba, H., He, L., Betsholtz, C., et al. (2015). Cell types in the mouse cortex and hippocampus revealed by single-cell RNA-seq. Science *347*.

37. Zeisel, A., Hochgerner, H., Lönnerberg, P., Johnsson, A., Memic, F., van der Zwan, J., Häring, M., Braun, E., Borm, L.E., La Manno, G., et al. (2018). Molecular Architecture of the Mouse Nervous System. Cell *174*, 999–1014.e22. https://doi.org/10.1016/J.CELL.2018.06.021.

38. Osakada, F., and Callaway, E.M. (2013). Design and generation of recombinant rabies virus vectors. Nat. Protoc. *8*, 1583–1601. https://doi.org/10.1038/nprot.2013.094.

39. Osakada, F., Mori, T., Cetin, A.H., Marshel, J.H., Virgen, B., and Callaway, E.M. (2011). New Rabies Virus Variants for Monitoring and Manipulating Activity and Gene Expression in Defined Neural Circuits. Neuron *71*, 617–631. https://doi.org/10.1016/j.neuron.2011.07.005.

40. Inoue, H., Nojima, H., and Okayama, H. (1990). High efficiency transformation of Escherichia coli with plasmids. Gene *96*, 23–28. https://doi.org/10.1016/0378-1119(90)90336-P.

41. Ciabatti, E., González-Rueda, A., Mariotti, L., Morgese, F., and Tripodi, M. (2017). Life-Long Genetic and Functional Access to Neural Circuits Using Self-Inactivating Rabies Virus. Cell *170*, 382–392.e14. https://doi.org/10.1016/j.cell.2017.06.014.

42. Hochgerner, H., Singh, S., Tibi, M., Lin, Z., Skarbianskis, N., Admati, I., Ophir, O., Reinhardt, N., Netser, S., Wagner, S., and Zeisel, A. (2023). Neuronal types in the mouse amygdala and their transcriptional response to fear conditioning. Nat. Neurosci. *26*, 2237–2249. https://doi.org/10.1038/s41593-023-01469-3.

43. Ting, J.T., Daigle, T.L., Chen, Q., and Feng, G. (2014). Acute brain slice methods for adult and aging animals: Application of targeted patch clamp analysis and optogenetics. Methods Mol. Biol. *1183*, 221–242. https://doi.org/10.1007/978-1-4939-1096-0_14.

44. Gerlach, J.P., van Buggenum, J.A.G., Tanis, S.E.J., Hogeweg, M., Heuts, B.M.H., Muraro, M.J., Elze, L., Rivello, F., Rakszewska, A., van Oudenaarden, A., et al. (2019). Combined quantification of intracellular (phospho-)proteins and transcriptomics from fixed single cells. Sci. Rep. *9*, 1469. https://doi.org/10.1038/s41598-018-37977-7.

STAR★METHODS

KEY RESOURCES TABLE

REAGENT or RESOURCE	SOURCE	IDENTIFIER
**Bacterial and virus strains**		
XL1-Blue MRF' Kan Supercompetent Cells	Agilent	Cat# 200248
ssAAV-2/1-Syn-H2B-mCherry-TVA-N2c.G-WPRE3	This paper	Cat# BA-413, serotype 1, VCF Charite Berlin
ssAAV-8/2-hCMV-hHBbI/E-TVA_mCherry_2 A_oG-hGHp(A)	This paper	Cat# v534, serotype 8, VVF Zürich
BC-DG-RV barcoded library of rabies virus (SADB19) with GFP or BFP	This paper	N/A
**Chemicals, peptides, and recombinant proteins**		
Lipofectamin 3000	Thermo Fisher Scientific	Cat# L3000150
Recombinant RNase Inhibitor	Takara	Cat# 2313 A
Maxima H Reverse Transcriptase	Thermo Fisher Scientific	Cat# EP0753
KAPA HiFi HotStart ReadyMix	Roche/Kapa	Cat# 07958935001
SPRIselect DNA Size Selection	Beckman Coulter	Cat# B23318
DSP	Thermo Fisher	Cat# 22585
SPDP	Thermo Fisher	Cat# 21857
**Critical commercial assays**		
Megaprep Kit Plasmid DNA	Macherey Nagel	Cat# 740576
Chromium Next GEM Single Cell 3' GEM	10× Genomics	Cat# 1000121
Visium Gene Expression	10× Genomics	Cat# 1000187
Stereo-Seq Transcriptomics Set v1.2	STOmics	Cat# 111KT114
**Deposited data**		
Single-cell RNA-seq	This paper	https://doi.org/10.6084/m9.figshare.29952065
Spatial transcriptomics 10× Visium	This paper	https://doi.org/10.6084/m9.figshare.29952065
Spatial transcriptomics STOmics Stereo-Seq	This paper	https://doi.org/10.6084/m9.figshare.32204544
**Experimental models: Cell lines**		
B7GG cells	Osakada et al.[38]	N/A
BHK-EnvA cells	Osakada et al.[38]	N/A
HEK293T-TVA cells	Osakada et al.[38]	N/A
**Experimental models: Organisms/strains**		
Mouse: C57BL/6JOlaHsd	Envigo	ENVIGO:057; MGI:2164189
**Oligonucleotides**		
N21Bsi2: ccctcgctgctcgtacggtgnnnnn nnnnnnnnnnnnnnnnGTGTCAGATTATATCCCGCA	This paper	N/A
N30Pst: GGCGATGGATCCTGCAGGTCC	This paper	N/A
T1-UMI-RV_M: TTTCCCTACACGACGCTCTTCCGATCTN NNNNNNNNNNNNTGTTATATTGCTCCCAAGGGTTGG	This paper	N/A
T1- ROI_index UMI-OdT: CTACACGACGCTCTTCCGATCTNNNNNTAGCTA NNNNNNNNNNNNNNNNNNTT TTTTTTTTTTTTTTTTTTTTTTTTTTTTTVN	This paper	N/A
RV_M-T2-PCR-forward: GTGACTGGAGTTCAGACGTGTGCTCTTCCGAT CTGGACAAAGATTCCTCTCTGCT	This paper	N/A
T1-PCR-reverse: ACACTCTTTCCCTACACGACGCTC	This paper	N/A

(Continued on next page)

Continued

REAGENT or RESOURCE	SOURCE	IDENTIFIER
AAV-T2-PCR forward: GTGACTGGAGTTCAGACGTGTG CTCTTCCGATCTGACGTGCACGAACGGATCAGTG	This paper	N/A
Recombinant DNA		
pSADdeltaG-GFP-F2	Osakada et al.[38]	Addgene #32635
BC-plasmid library D151pSADdeltaG-FP-F2-ins2	This paper	N/A
pcDNA-SADB19N	Osakada et al.[39]	Addgene #32630
pcDNA-SADB19P	Osakada et al.[39]	Addgene #32631
pcDNA-SADB19L	Osakada et al.[39]	Addgene #32632
pcDNA-SADB19	Osakada et al.[39]	Addgene #32633
pAAV-CMV-TVAmCherry-2A-oG		Addgene #104330
pAAV-EF1a-DIO-HTB	Edward Callaway, unpublished	Addgene #44187
pCAG-N2cG	Ian Wickersham, unpublished	Addgene #100811
Software and algorithms		
Original code	This paper	https://doi.org/10.6084/m9.figshare.29952065 https://github.com/zeiselamit/ROInetseq
Other		
Silica column	Geneaid Biotech	Cat# DFH300

EXPERIMENTAL MODEL AND STUDY PARTICIPANT DETAILS

Adult wild-type C57BL/6JOlaHsd mice (6–8 weeks, females) were purchased from Envigo and were group housed at the Technion SPF Pre-Clinical Research Authority under standard conditions in a 12-h light:dark cycle, at ambient temperature 21°C–23°C and and 30–40% relative humidity. Standard chow and water were provided *ad libitum*. This study used females only for improved post-surgical recovery under group-housing conditions. This choice potentially affects generalizability of the described connectivity patterns, as reported for sex dimorphic connectivity in other brain regions.[23] All animal procedures followed the legislation under the National Institute of Health guidelines and were approved by the Institutional Animal Care and Use Committee of the Technion Israel Institute of Technology.

For rabies virus generation in this study, B7GG, BHK-EnvA, and HEK293T-TVA cell lines were used.[39] B7GG and BHK-EnvA cells were maintained at 37°C with 5% CO_2 and cultured in DMEM supplemented with 10% fetal calf serum (FCS) and 1% penicillin/streptomycin. For virus production, these cells were incubated at 35°C with 3% CO_2 under otherwise identical culture conditions. HEK293T-TVA cells were maintained at 37°C with 5% CO_2 in DMEM supplemented with 10% FCS and 1% penicillin/streptomycin.

B7GG cells exhibited distinct morphological features, including a rounded cell shape and a fluorescently labeled nucleus. BHK-EnvA cells displayed an elongated morphology comparable to wild-type BHK cells. HEK293T-TVA cells did not show obvious morphological differences compared to standard HEK293T cells.

METHOD DETAILS

Plasmid library generation ΔG-SAD-B19

All cloning was conducted at DNA Cloning Service, Hamburg (https://dna-cloning.com). A DNA fragment of 263bp was amplified with Pfu from vector D150pSADdeltaG-GFP-F2-ins1 using the primers:

N21Bsi2: ccctcgctgctcgtacggtgnnnnnnnnnnnnnnnnnnnnnGTGTCAGATTATATCCCGCA.

N30Pst: GGCGATGGATCCTGCAGGTCC.

The amplified DNA from 8 × 50µL PCR tubes was purified with 8× DFH300 silica column (Geneaid Biotech). The total yield was 3.4µg. 486ng of the purified PCR fragments where digested with BsiWI and PstI and cloned into the corresponding restriction sites of 18µg plasmid D151pSADdeltaG-GFP-F2-ins2 in a total volume of 60µL. Chemical competent E. coli cells (XL1-Blue MRF’ Kan, Agilent) were prepared by the method of Inoue et al.[40] To 60 tubes with 50µL cells 1µL ligation reaction was added, incubated for 5min, heat shocked at 42°C and regenerated for 20min with 450µL SOC medium. An aliquot of the collected transformation reactions was used to calculate the complexity of the library, which was around 812.000 transformated cells. The cells from one plate were harvested with 5mL TB-medium and grown in 100mL TB-medium for 6 h to an OD600 of 1. About 7.5mg DNA was isolated from 2 L cells with the Megaprep Kit (Macherey Nagel).

Barcoded ΔG-SAD-B19 rabies virus generation

Pseudotyped Rabies virus particles were prepared at the Viral Core Facility of the Charité – Universitätsmedizin Berlin (vcf.charite.de) using standard protocols.[38] Briefly, in order to recover G-deleted rabies virus from cDNA, B7GG cells (gift from Edward Callaway, Salk Institute) were transfected with a mix of the rabies virus genomic vector D151pSADdeltaG-GFP-F2-ins2 (Barcode vector BR-16) or D151pSADdeltaG-BFP-F2-ins2 (BR-17) (internal numbering), pcDNA-SADB19N (Addgene plasmid # 32630), pcDNA-SADB19P (Addgene plasmid # 32631), pcDNA-SADB19L (Addgene plasmid # 32632) and pcDNA-SADB19G (Addgene plasmid # 32633). All vectors were gifts from Edward Callaway.[39] Transfection was performed with Lipofectamin 3000 (ThermoFischer Scientific).

BR-16.1

Three days after transfection of three 75cm^2 flasks (80% confluency), cells were transferred into three 175cm^2 flasks. After 3 to 4 days supernatant was collected and 15mL per flask were used for infection of another three 175cm^2 flasks (80% confluency). This was repeated 4 more times to amplify the virus. Finally, five 175cm^2 flasks (80% confluency) were used for the infection with the 7mL amplified virus supernatant. After inoculation of further 4 days the supernatant was harvested and filtered through a 0.45μm filter.

BR-16.2/BR-16.3

Three days after transfection of three 75cm^2 flasks (80% confluency), cells were transferred into three 175cm^2 flasks. After 3 to 4 days supernatant was collected and 20mL per flask were used for infection of another two 175cm^2 flasks (80% confluency). This was repeated 2 more times with four 175cm^2 flasks to amplify the virus. After inoculation of further 4 days the supernatant was harvested and filtered through a 0.45μm filter.

BR-16.4/BR-16.5/BR-16.6 and *BR-17.1*

Three days after transfection of ten 75cm^2 flasks (80% confluency), cells were transferred into ten 175cm^2 flasks. After 4 days supernatant was collected filtered through a 0.45μm filter and concentrated by ultracentrifugation (22 K rpm, SW32 rotor, Beckmann). 2mL per flask were used for infection of another four 175cm^2 flasks. After inoculation of further 4 days the supernatant was harvested and filtered through a 0.45μm filter. Production of BR-17.1 was identical, except that GFP in the rabies virus genome vector used had been replaced with BFP.

For pseudotyping the rabies with the envelope protein EnvA of the Avian Sarcoma and Leukosis virus (ASLV) the obtained supernatant containing unpseudotyped viruses was applied onto ten 175cm^2 flasks containing BHK-EnvA cells (90% confluency) (gift from Edward Callaway, Salk Institute) for 5 h followed by a transfer of the cells into new flasks with fresh media. After one day, media was changed a second time. After further 2 days EnvA-pseudotyped rabies viruses containing supernatant was collected, filtered and concentrated by ultracentrifugation. Rabies virus titer was determined by infecting a serial dilution of the virus in HEK293T-TVA cells (gift from Edward Callaway, Salk Institute). The efficiency of pseudotyping was tested *in vivo,* where we confirmed no infection by BC-ΔG-RV without prior helper virus administration (Figure S1).

Quality control barcoded ΔG-SAD-B19 *in vitro*

Barcodes counting is based on bulk amplicon sequencing. Briefly, HEK293T-TVA cells were infected with barcoded ΔG SAD-B19. 48h later, ∼20% of the cells were infected with rabies. Cells were then trypsinized and aliquoted into 10^5 cells per vial. Total RNA was extracted from 5 vials of cells with the RNA extraction kit (NucleoSpin, Macherey Nagel). To count the barcode number of each library, we reverse transcribed the RV-*M*-gene, using an *M*-complementary primer with the Trueseq Read 1 sequence, and a 12bp UMI (T1-UMI-RV_*M*). First-strand was carried out on 40ng total RNA, in a 20μL reaction (20U/μL Maxima H Reverse Transcriptase (Thermo), 2.5mM dNTP, 1μM T1-UMI-RV_M, 1 M Betaine, 6mM MgCl2 and 2U/μL RNAse Inhibitor), incubated 45min at 50°C, 5min at 85°C, then 4°C; followed by clean-up with 2× SPRI beads, and eluted in 10μL EB. Next, the RV barcoded region was amplified with primers complementary to RV-*M* gene RV_M-T2-PCR-forward (introducing Truseq 2) and Truseq 1 (T1-PCR-reverse) in a 20μL reaction (1× KAPA, 0.5 M Betaine, 0.5μM primer mix), 3min 98°C, then 16 cycles of 15s 98°C, 20s 60°C, 1min 72°C, finally 1min 72°C, followed by 1.2× SPRI beads cleanup, and elution in 10μL EB. Finally, sequencing adaptors P5 and P7 were introduced following the 10xGenomics dual-index setup: 20-50ng PCR product was added to library PCR mix (50% KAPA, 10% Betaine, 20% primer Dual index (Illumina)), total 20μL; 45s 98°C, then 16 cycles of 20s 98°C, 30s 54°C, 20s 72°C finally 1min 72°C. PCR was followed by double-sided SPRI clean-up (0.6× SPRI and 0.8× SPRI). Amplicon libraries were sequenced on Illumina NextSeq2000.

Viral injections

In vivo barcoded monosynaptic tracing is based on two stereotactic injections for AAV helper virus and RV respectively as described previously. Briefly, surgeries were performed on naive C57BL/6 mice, aged 6–8 weeks. Mice were anesthetized using isoflurane, and maintained on 1.8% isoflurane (SomnoSuite Kent Scientific). Anesthetized mice were fixed onto the stereotactic rig (Neurostar). Optic ointment (Duratears) was applied on both eyes and body temperature was maintained at 37°C with a heating pad. We observed heart rate and oxygen saturation throughout the procedure. Craniotomy was performed after applying Lidocain/Bupivacaine to the skull for local pain relief, with a 200μm diameter drill. Virus was injected with pulled glass pipette at steady rate (25-50nL/min). After injection, we waited 8–10 min before withdrawing, to prevent reflux. Target coordinates were obtained from The Mouse Brain in Stereotaxic

Coordinates, 3rd edition, Keith B.J. Franklin, George Paxinos. Buprenorphine (0.05–0.1mg/kg) was given for analgesia on the day of surgery and the day after. 21±4 days after AAV injection, RV was injected to the same target, following the same surgical procedure. Brains were harvested 7±1 day after RV injection.

For the scRNA-seq and Visium ST experiments we used helper virus with the B19 optimized glycoprotein; ssAAV-8/2-hCMV-hHBbI/E-TVA_mCherry_2 A_oG-hGHp(A). This virus was generated by Viral Vector Facility VVF Zurich (v534 serotype 8, VVF Zürich), based on pAAV-CMV-TVAmCherry-2A-oG; a gift from Marco Tripodi (Addgene #104330).[41]

For all other surgeries, we used helper virus with the N2c-version of glycoprotein; ssAAV-2/1-Syn-H2B-mCherry-TVA-N2c.G-WPRE3. This virus was generated by the Viral Core Facility of the Charité Universitätsmedizin Berlin (Cat# BA-413, serotype 1), based on Addgene plasmids pAAV-EF1a-DIO-HTB (#44187, gift from Edward Callaway) and pCAG-N2cG (#100811, gift from Ian Wickersham).

Details for BC-ΔG-RV libraries are provided in the section "Barcoded ΔG-SAD-B19 rabies virus generation".

scRNA-seq of barcoded δg-SAD-B19 injected brains

To isolate single BC-ΔG-RV (GFP-library BR-16.1) infected cells for scRNA-seq, we modified a protocol for gentle adult neuron dissociation.[42] 8 days after bilateral stereotaxic injections to the CA1, one mouse was sacrificed by an overdose of Ketamine/Xylazine, and transcardially perfused with ice-cold carboxygenated NMDG-aCSF.[43] The brain was extracted, then mounted and sectioned on a Leica VT1200S automated vibrating microtome, to 500μm slices. Fluorescent regions were quickly microdissected in ice-cold aCSF under a fluorescent stereomicroscope (Nikon SMZ18). Next, collected tissue pieces were digested in 800-100μL papain digest solution with DNase (Worthington Papain system, concentrations as recommended by manufacturer, but vials were reconstituted in aCSF), 25min at 35°C–37°C. Starting 15min into incubation, tissue was gently aspirated in wide-bore fire-polished Pasteur pipettes and returned to incubate, until most tissue easily broke apart. We filtered the suspension through a 30μm strainer (CellTrix, Partec) to ice-cold microtubes coated with 30% BSA, and pelleted cells at 4°C, 5min, 200 g. Supernatant was fully removed, cells immediately resuspended in 500μL fixative (DSP and SPDP in sodium phosphate buffer pH8.4),[44] and incubated 40min, at RT, gently shaking. The fixation was stopped with 1 volume quenching buffer (0.1 M Tris-HCl 150mm NaCl, pH7.4) 10min, fixed cells pelleted, and resuspended in PBS with 0.3%BSA and 1% recombinant RNase inhibitor (RRI, Takara Bio). Next, we enriched for GFP+ cells on a FACS Aria II (BD), or Bigfoot Spectral Cell Sorter (Thermo Fisher). ∼19 k sorted cells were immediately processed for encapsulation and scRNA-seq on the 10xChromium NextGEM, in two samples, following the manufacturer's instructions. Libraries were sequenced on the Illumina NovaSeq NGS platform, at a depth of ∼60 K reads per cell.

Histology and ROInet-seq

Histology: 7 days after RV injection, mice were sacrificed after an overdose of Ketamine/Xylazine, and transcardially perfused with PBS followed by 4% paraformaldehyde (PFA). Brains were dissected, postfixed in 4% PFA overnight and cryoprotected in 30% sucrose solution with PBS for 24 h. After OCT embedding, brains were cryosectioned full-volume to 50μm floating sections, mounted to Superfrost Plus slides (Epredia), counterstained (NucBlue, Thermo) and coverslipped (Fluoromount-G, Thermo). Images were taken using an epifluorescence microscope (Nikon Eclipse Ti2) at 4× magnification. Slides were stored at -80°C until further use.

ROI microdissection and lysis: For ROInet-seq, coverslips were gently removed in PBS, and under a fluorescent stereoscope, ROIs (tissue with RV-tagged cells) were microdissected on ice into stripes filled with lysis buffer (30mM Tris-HCL pH 7.5, 10mM EDTA, 1% SDS, 0.1μg/μl Proteinase K). Dissected tissue was vortexed vigorously and lysed for 1 h at 50°C, followed by 10min at 70°C, then placed on ice. Lysates were stored at -80°C until further use.

Bead preparation and polyA capture: To capture all polyA-transcripts, introduce a 12bp UMI, 16bp ROI-specific indexes, and Truseq read 1, we loaded RT-primer (T1-ROI_index UMI-OdT) to Streptavidin beads, as follows. First, Streptavidin-C beads (5μL per ROI, Thermo) were washed twice with 2xBWT buffer (10mM Tris-HCl pH 7.5, 1mM EDTA, 2 M NaCl) and resuspend with equal volume of 2xBWT buffer. In separate wells, 3μL of each unique RT primer (T1-ROI_index UMI-OdT) were added to 5μL washed beads, completed with UPW to total volume of 10μL, mixed and incubated for 20min in room temperature, 1200rpm. After incubation, RT primer-loaded beads were magnetically immobilized and washed with TNT (20mM Tris-HCl pH 7.5, 50mM NaCl, 0.02% Tween 20) and resuspend with Capture mix (50μL per ROI_index: 2xBWT, 0.5U/μl RRI (Takara)). Equal volume of lysate was added to capture mix and incubated for 1min at 56°C followed by 20min incubation at room temp, shaking 2000rpm. After incubation beads were placed on a magnet, supernatant was removed and beads were washed thoroughly 3× with ice-cold LiWT buffer (10mM Tris-HCl pH 7.5, 1mM EDTA, 150mM LiCl) and resuspend with 10μL UPW (suitable for 3 reactions). RT primers with captured polyA-transcripts were released from beads by 2min incubation at 80°C, and transferred. Samples were stored at -80°C until further use.

Reverse transcription: Reverse transcription was carried out on 2.7μL sample, by adding RT mix (1× Maxima buffer, 20U/μl Maxima enzyme, 1 M Betaine, 2mM dNTPs, 2U/μl RRI, 20mM DTT, 6mM MgCl2) to a total reaction volume of 6μL, and incubating 45min at 50°C, 5min at 85°C, then 4°C.

Clean-up: After RT, samples were cleaned up using 2× volumes CleanUp buffer (5 : 1 volumes of SPRI: Guanidine Thiocyanate 6 M solution) by 10min incubation followed by 2 × 30s 80% ethanol wash using bead magnet, 1min air dry and elution with 7.4μL elution solution 1 (1× Reducing Agent B (10xGenomics), 0.01% Tween 20 in buffer EB (Qiagen)).

PCR amplification: The RV barcoded region was amplified with primers complementary to RV-*M* gene (introducing Truseq read 2) and Truseq read 1, as follows. The eluate was added to 12.6μL PCR mix (1× KAPA HS, 0.5 M Betaine. Total reaction volume 20μL)

with 1μM primers (RV_M-T2-forward and T1-PCR-reverse), for targeted amplification of the RV M-gene with BC; 3min 98°C, then 17 cycles of 15s 98°C, 20s 60°C, 1min 72°C, finally 1min 72°C. After PCR, samples were cleaned with 1.2× SPRI protocol and finally eluted in 10μL EB. Sample concentration was evaluated using Qubit.

Library indexing and sequencing: Finally, sequencing adaptors P5 and P7 were introduced following the 10xGenomics dual-index setup: 20-50ng PCR product was added to library PCR mix (50% KAPA, 10% Betaine, 20% primer Dual index (Illumina)) total 20μL; 45s 98°C, then 16 cycles of 20s 98°C, 30s 54°C, 20s 72°C finally 1min 72°C. At this point, all ROI samples were pooled to one tube, followed by double-sided SPRI clean-up (0.6× SPRI and 0.8× SPRI). Concentration was evaluated using Qubit and fragment size on Bioanalyzer or TapeStation. The final ROIs amplicon libraries resemble 10xChromium v3/NextGEM sequencing libraries and were sequenced at a depth of ~10 M reads per sample (one brain) on Illumina NextSeq2000.

In its final implementation, a typical ROInet-seq experiment was microdissected and processed for RNA-sequencing within 2 experimental days (day 1, microdissection and lysis; day 2, molecular biology processing); when preceded by ~1 day for careful brain alignment and ROI-dissection strategy design. PCR amplification introducing the final library indexes can in principle be carried out with all ROI samples in pool. This saved ~2 h of manual handling time but increased representation of noisy BCs; requiring more stringent computational processing, detailed in "ROInet-seq data processing and analysis".

Stereo-seq cell-resolved spatial transcriptomics networks reconstruction

Tissue collection and spatial transcriptomics: Transcardial perfusion with aCSF was performed under deep anesthesia on the harvest day. Brains were snap frozen on dry ice and stored at -80°C until sectioning the following day. Tissue mounting was performed according to the steps outlined by the manufactorer (STOmics, Stereo-seq transcriptomics set for chip-on-a-slide V1.2, manual version A1). The tissue was sectioned into 10μm-thick slices, and the target region (hippocampus) was trimmed with a blade and transferred onto Stereo-seq transcriptomics chips, such that each chip contained nine consecutive hippocampal sections. The anterior-posterior distance between every two consecutive sections was 50μm. Subsequent steps, including image acquisition, tissue permeabilization, reverse transcription, cDNA release and amplification, and library preparation, were all carried out according to the protocol.

Visium ST networks sequencing and analysis

Tissue collection and spatial transcriptomics: Transcardial perfusion with aCSF was performed under deep anesthesia on the harvest day. Brains were snap frozen on dry ice and stored at -80°C until sectioning the following day. The brain was cryo-sectioned into 20μm, by cutting only the hippocampus region and collecting to capture areas on the Visium Spatial Transcriptome slide (Figures 6 and S8). The gap between two consecutive sections collected to the capture area was 100μm. All adjacent sections were collected to a SuperFrost Plus Slide (Epredia) and stored at -80°C until processing. Visium ST slides were processed as per the manufacturer's instructions, with the following specifications: Slides were fixed with methanol, then DAPI counterstained and imaged on an inverted epifluorescence microscope (Nikon Eclipse Ti2). Tissue was permeabilized for 30 min. The mRNAs from the sections were then captured by the primers including UMI, spatial barcode and sequencing primer on the Visium ST slides. After the RT reaction and second strand synthesis on slides, the cDNA library was transferred into tubes, fragmented and indexed. For detection of RV-barcodes and AAV transcripts, cDNA was additionally amplified in two separate PCR reactions, with RV_M-T2-PCR-forward/T1-PCR-reverse (RV-*M*-gene) or AAV-T2-PCR forward/T1-PCR-reverse (AAV-*oG*-gene), using 20-30ng cDNA, each in a 10μL reaction (1× KAPA, 0.5 M Betaine, 1μM primer mix), 3min 98°C, then 8 cycles (RV-*M*) or 10 (AAV-*oG*) cycles of 15s 98°C, 20s 53°C, 1min 72°C, finally 1min 72°C, followed by 1× SPRI beads cleanup, and elution in 4μL EB. Finally, sequencing adaptors P5 and P7 were introduced following the 10xGenomics dual-index setup: Eluted PCR product was added to library PCR mix (50% KAPA, 10% Betaine, 20% primer Dual index (Illumina)), total 20μL; 45s 98°C, then 12 cycles of 20s 98°C, 30s 54°C, 20s 72°C finally 1min 72°C. PCR was followed by double-sided SPRI clean-up (0.6× SPRI and 0.8× SPRI). Amplicon libraries were sequenced to 140-260 M reads per sample on Illumina NextSeq2000.

osmFISH

To detect viral transcripts in sections adjacent to the ones sampled for Visium ST, we performed *in situ* hybridization according to osmFISH,[19] with minor modifications. We collected 10μm sections to Superfrost Plus slides (Epredia) and stored them at -80°C until processing. After the sections were balanced at RT, the tissue was fixed in 4% PFA for 10 min, washed 5 min with 2× SSC buffer, and cleared with 8% SDS for 30 min. Tissue is incubated with hybridization mix (2× SSC, 10% dextran sulfate, 10% formamide, 1mg/mL E. coli tRNA, 2mM ribonucleoside vanadyl complexes, 0.2mL/mL bovine serum albumin) without probes for 5 min at RT and then 4 h at 38.5°C in hybridization mix with probes (250nM). After wash with 20% formamide in 2× SSC, slides were mounted (ProLong Diamond Antifade, Thermo) and imaged at 10× magnification, on an inverted epifluorescence microscope (Nikon Eclipse Ti2).

QUANTIFICATION AND STATISTICAL ANALYSIS

ROInet-seq data processing and analysis

BC detection and library verification. ROInet-seq fastq files contained ROI-index, UMI (both Read 1) and the target sequence on the 3′UTR of the RV-*M*-gene, including the BC-read (Read 2). First, we aligned ROI-index reads with the known list of ROIs wells that were collected. In Read 2, we called the BC read by aligning the known, permanent sequences flanking the BC in the *M*-3′UTR, and

extracting every variable 21nt sequence inside. Reads per ROI were restricted to 1000 per fluorescent cell detected in that ROI. This was necessary since sequencing depth per ROI could not be accurately controlled in the pooled setup. Only BCs that were detected in the *in vitro* library (>10 reads in HEK-TVA culture, see "Quality control barcoded ΔG-SAD-B19 in vitro") were included in analysis.

BC detection thresholds. Building on quantitative BC detection with unique molecular identifiers (UMIs), we next applied the following thresholds: For confident detection, any BCs detected with under 50 UMIs over all ROIs were removed from downstream analysis. This threshold disproportionally removed BCs labeling smaller networks that were of lesser interest to the current work ("Sum UMI BC threshold", Figures 4A and S4). Per ROI, BCs were included only when detected at a threshold of 10UMIs. This reduced average detected network size in favor of robust detection ("UMI per ROI BC threshold", Figures 4A and S4).

Focus on confident, wide-spread network BCs. Next, we restricted network analysis to the most widely spread unique BCs, relative to the number of recorded starter cells in the respective brain. First, unique BCs were ranked by network size (i.e., number of ROIs where the BC was detected; Figures 4B and S4)). We then included all top ranked BCs up to the number of counted starter cells plus 50% ("1.5× starter cells threshold"). This was based on the observation that individual BC-ΔG-RV-infected cells in scRNA-seq (Figure S2) and Stereo-seq ST (Figure 7) contained no more than ~1.5BCs per starter cell. In the current analysis, the BC-spread ranking was applied to preferentially analyze BCs that (1) were detected with a high degree of confidence and that (2) labeled larger networks.

Figures 3 and S3 present all BCs passing these criteria. Further, central biological findings were reproduced independent of BC thresholds, and across a wider inclusion of networks (Figure S6; overrepresented convergent inputs motives; divergent thalamic inputs to SSp).

Injection site and abundance filter. Here, we filtered BCs not detected in the injection site (compare Figure 3G). Finally, to avoid the risk of multicell-infection of identical BCs, we removed BCs that were highly abundant in the *in vitro* library (>10^{-4} in HEK-TVA) or in all three *in vivo* experiments (>10^{-4} in 98-1, -5 *and* -6) (Figure 4C). As expected for abundant BCs, where a single BC was likely to infect several starter cells, this step disproportionally removed wide-spread BCs (Figure 4D) and likely reduced the detection of erroneously large networks.

Together, this pipeline resulted in 627 (*98-1*), 2057 (*98-5*) and 879 (*98-6*) BCs that were included toward the final BC count matrix (Table S1), used to plot overlaps of BCs between ROIs, and global or single-barcode networks.

Visium ST networks sequencing and analysis

Data analysis: The methodology for analyzing Visium data and detecting barcodes is implemented through a comprehensive MATLAB script designed for the integration of rabies barcode information with spatial context derived from Visium experiments. It proceeds to process barcode data from distinct experimental conditions, including Vis11_A1, Vis11_B1, Vis11_C1, and Vis11_D1, as well as RFP-AAV SAM files. This involved organizing data into: cell IDs, unique sequences, and read frequencies, leading to the creation of a unified table combining rabies and AAV data. Further, since sequencing depth of individual capture spots was unequal, we next normalized spots to each other; accounting for the total number of reads per spot, and the number of BCs detected in that spot, as follows: For each spot, the total read count was divided by the square root of the number of unique BCs in that spot. Finally, we filtered noisy BCs, by removing BCs that were below an average expression threshold or had null empty entries. Genes and BCs shown in the Figures were plotted in a binary fashion, i.e., each spot with a detected gene or BC is shown positive. We further incorporate functionality to subset and process specific barcode sequences from FASTQ files, saving the resulting subset and identifying common elements with loaded barcodes. Additionally, we used DAPI images of the Visium ST sections to map each section in its anatomical context by semi-manual aligning sections to the Allen Reference Atlas—Mouse Brain (atlas.brain-map.org) and correcting for distortion caused by sectioning along the dorsoventral and mediolateral axes. Sections were on average 100μm apart. mRNA-capture spot diameters were 55μm, centers 100μm apart (x–y resolution). 18,177 capture spots covered the tissue and contained on average ~3,300 genes and ~9,900 UMIs. 1848 of the capture spots were positive for RV (expressing positive values higher than one). We called the BC read by aligning the known, permanent sequences flanking the BC in the *M*-3′UTR and extracting every variable 21nt sequence inside. We finally corrected for sequencing errors by pooling any BCs with Hamming distance of ≤3 with its most abundant version.

Stereo-seq cell-resolved spatial transcriptomics networks reconstruction

Spot clustering: Bin50 gene expression matrix of all 27 sections was loaded and spots with <300 UMI were excluded. All data was scaled to a sum of 3000 counts per spot. Genes expressed in less than 200 spots, or in more than 80% of spots, were excluded. To cluster the spots, we applied our standard scRNA-seq pipeline,[42] except that we used UMAP instead of tSNE embedding. Clusters (resulting from DBSCAN) were visualized on UMAP and in the sections; where anatomical features appear as a greyscale of the number of total detected molecules in the spot (light, low UMI; dark, high UMI). No additional constrain was added to generate spatially continuous clusters.

Network cells segmentation: To identify potential network cells, we first summed the number of detected molecules for rabies transcripts *GFP*, *M* and *P* per spot. Spots with detected RV-expression that had at least 5 neighbors in a radius of 20 spots (~10μm) were retained for segmentation of RV cell somas. We next performed spatial clustering using DBSCAN (epsilon 15, minpoint 5). This resulted in 1893 clusters that were putative RV-infected cells and were segmented. We further subdivided clusters larger than 4000 pixels using k-means (k = #of pixels/4000; rounded to nearest greater integer). Then, clusters smaller than 100 were discarded.

Next, all BC reads with x-y position within the bounds of remaining clusters were assigned to these clusters. When the highest represented BC within a cluster was not detected by at least 3 reads, this cluster was discarded, too. The remaining clusters were considered as putative network cells (n = 774); by their RV transcript expression, cell size and BC detection, and are seen as yellow outlines (Figure 7) or black outlines (Figure S10). All clusters that were discarded according to these criteria are seen as blue outlines in Figure 6 (n = 1119). Finally, all BCs supported by just 1 read per putative network cell were discarded. All remaining BCs are analyzed as networks and presented in Figures 7 and S10.

Cell Reports Methods, Volume *6*

Supplemental information

Mapping brain-wide monosynaptic

inputs to single neurons with ROInet-seq

Zhige Lin, Osnat Ophir, Thorsten Trimbuch, Bettina Gann, Muhammad Tibi, Hermann Schmidt, Christian Rosenmund, Hannah Hochgerner, and Amit Zeisel

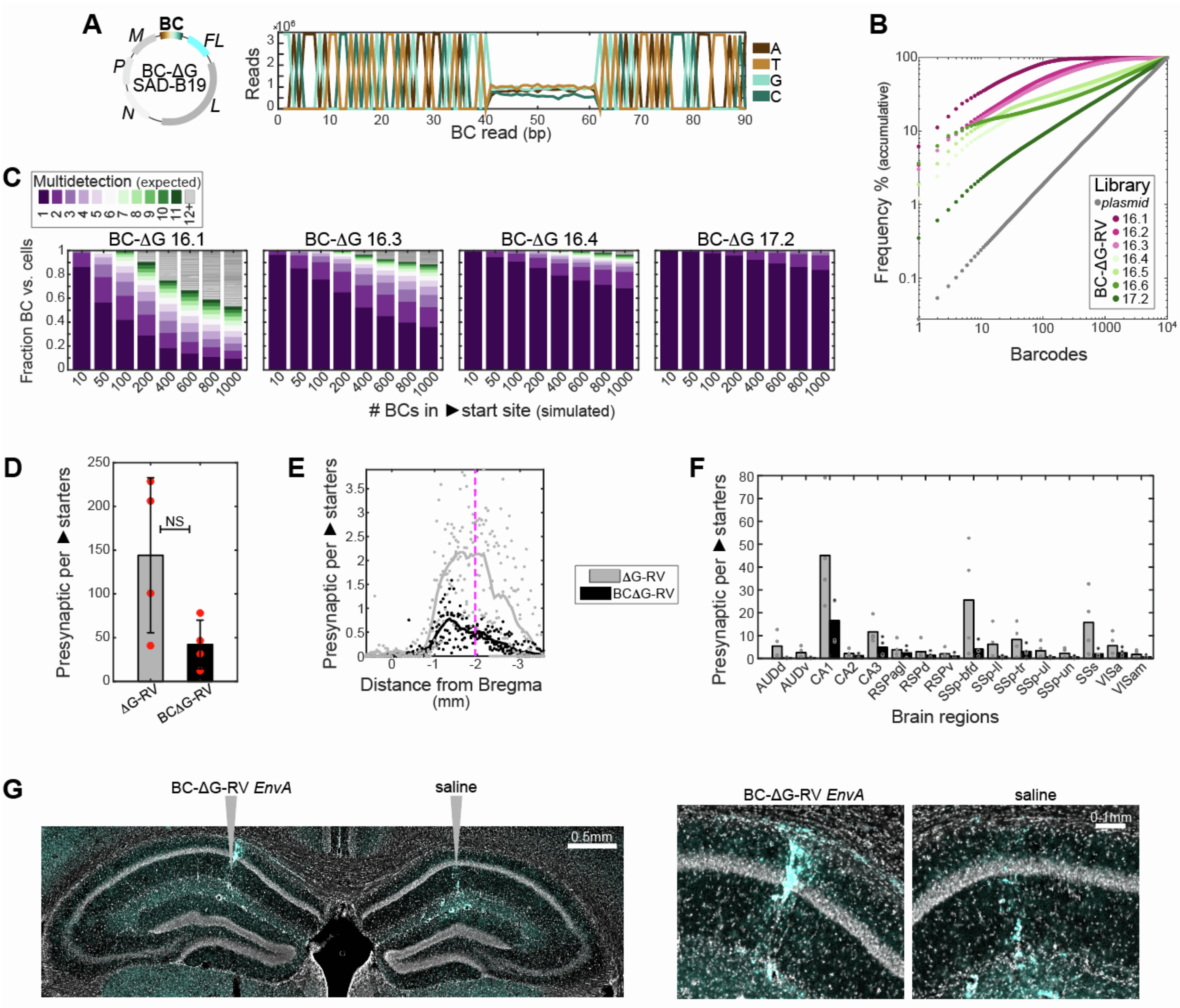

Figure S1. Characterization of BC-DG-RV libraries. Related to Figure 1.

(**A**) Sequencing verification of a uniformly random 21nt BC insert, shown as pseudo-Sanger bp frequency plot.

(**B**) Accumulative frequency of top 10k most frequent barcodes per RV-library, vs. the optimally uniform plasmid library.

(**C**) Expected detection of a unique BC in 1-12+ cells, given 8 scenarios based on 10-1000 starter cells, per library.

(**D**) Quantification of presynaptic-per-starter cells after infection to the CA1 (unilateral) with DG-RV (n=4; grey; 144.1 +/-88.5) and BC-DG-RV (n=4; black; 42.2+/-27.9).

(**E**) As (**D**), quantification of presynaptic-per-starter neurons resolved to the sections along the anterior-posterior axis. Pink line, injection coordinates.

(**F**) As (**D**), quantification of presynaptic-per-starter neurons, summed by brain region.

(**G**) *In vivo* EnvA pseudotype test. Without prior infection with AAV helper virus, we unilaterally injected either BC-DG-RV (left) or saline (right). Both injections show signs of tissue damage along the injections' trajectories (increased autofluorescence), but no labelling of neuronal soma.

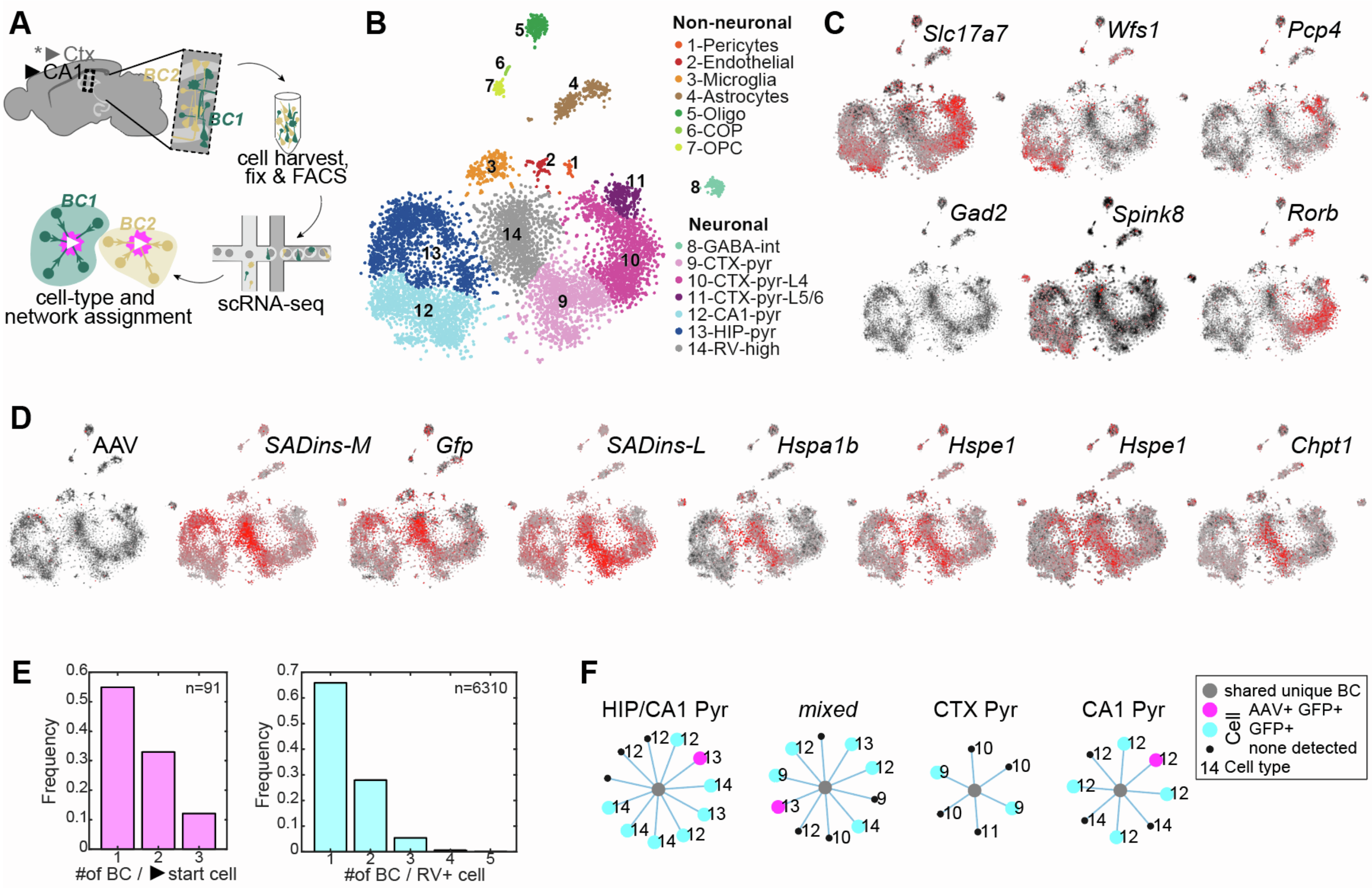

Figure S2. Single-cell RNA-seq on BC-DG-RV-infected neurons *in vivo*. Related to Figure 1.

(**A**) Experimental schematic, BC-DG-RV infection of hippocampus CA1, with secondary infection in isocortex dorsal to CA1, followed by dissociation, fixation, FACS and scRNA-seq; to simultaneously detect transcriptomes and BCs.

(**B**) tSNE visualization of 8032 cells, colored by cell type. Among 6979 neuronal transcriptomes, 129 were GABAergic interneurons, 2744 had a predominantly cortical pyramidal signature (1566 of which L4/5/6) and 2937 a hippocampal pyramidal signature (1418 of which CA1).

(**C**) Expression of six neuronal genes, in all cells visualized in (**B**). Grey, low; red, high; black circle, 0.

(**D**) In all cells visualized in (**B**), expression of AAV (any gene of *TVA, oG, mCherry*), and genes enriched to RV-high cluster 14. RV transcripts *SADins-M* and *Gfp* and *SADins-L*, and four further genes highly correlated with *SADins-M* expression. Grey, low; red, high; black circle, 0.

(**E**) Frequency of detecting single or multiple BCs in a single cell; in starter cells (91 neurons, as in Fig. 1F) or all BC-DG-RV+ cells (total, 6310 BC+ neurons).

(**F**) Four examples of potential single-cell networks; i.e., cells sharing a unique barcode. Over all networks, most have no verified starter cell (cell with AAV and GFP detected), and most have some degree of mixing of transcriptomic types from CTX and HIP, as shown in one example.

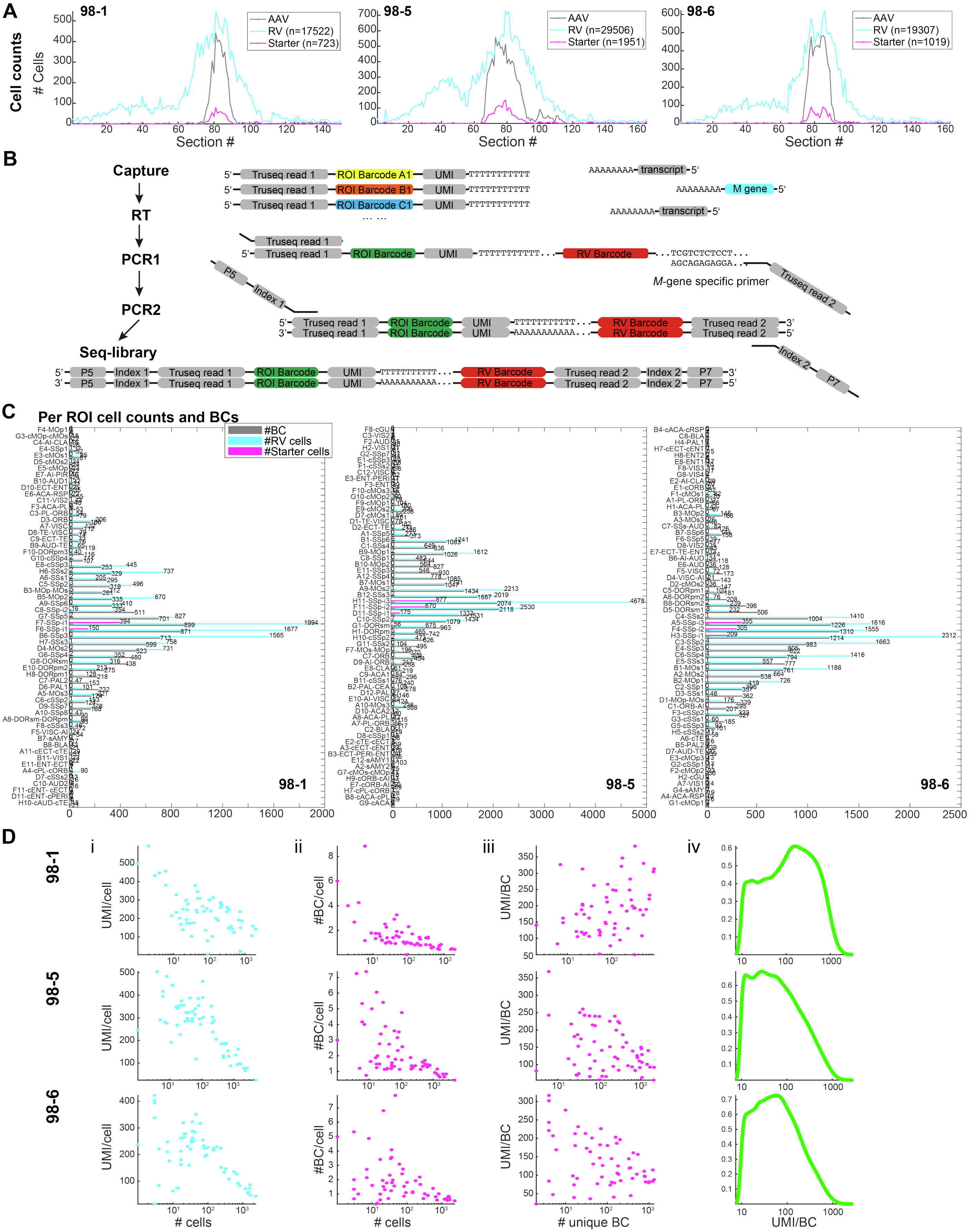

Figure S3. ROInet-seq analysis pipeline: Cell counts, workflow and raw BC detection. Related to Figures 2 and 3.

Figure S3, continued.

(**A**) After BC-DG-RV infection of the SSp, automated cell detection of AAV+ and RV+ cells across the full brain in anterior-posterior section order (50µm, gapless collection). Fluorescent overlap of signals (AAV+RV+) is considered a starter cell.

(**B**) Schematic of RT and BC-specific amplification from fixed tissue ROIs in ROInet-seq. Each ROI is collected to a separate well, loaded with lysis buffer and polyT primer carrying a unique ROI barcode (spatial index). In reverse transcription (RT), the indexed polyT captures polyA transcripts present in that ROI, including RV-genes such as *M* (with the network BC in its 3'UTR). cDNA is amplified in two consecutive PCR reactions, first using an oligo specific to a sequence flanking the *M*-gene (and BC), and then sequencing library prep to introduce library indexes and P5/P7.

(**C**) Summary of all ROIs per experiment, with imaging-based cell counts and BC detection.

(**D**) Per-ROI quantifications of cells, BCs and UMIs in ROInet-seq, per experiment (row). Each ROI is one dot (**i-iii**) (**i**) Scatterplot of UMI/cell as a function of number of cells detected (imaging). (**ii**) Scatterplot of BC/cell as a function of number of cells detected (imaging). (**iii**) Scatterplot of UMI/BC as a function of BCs. (**iv**) Distribution of copies (UMI) detected per unique BC.

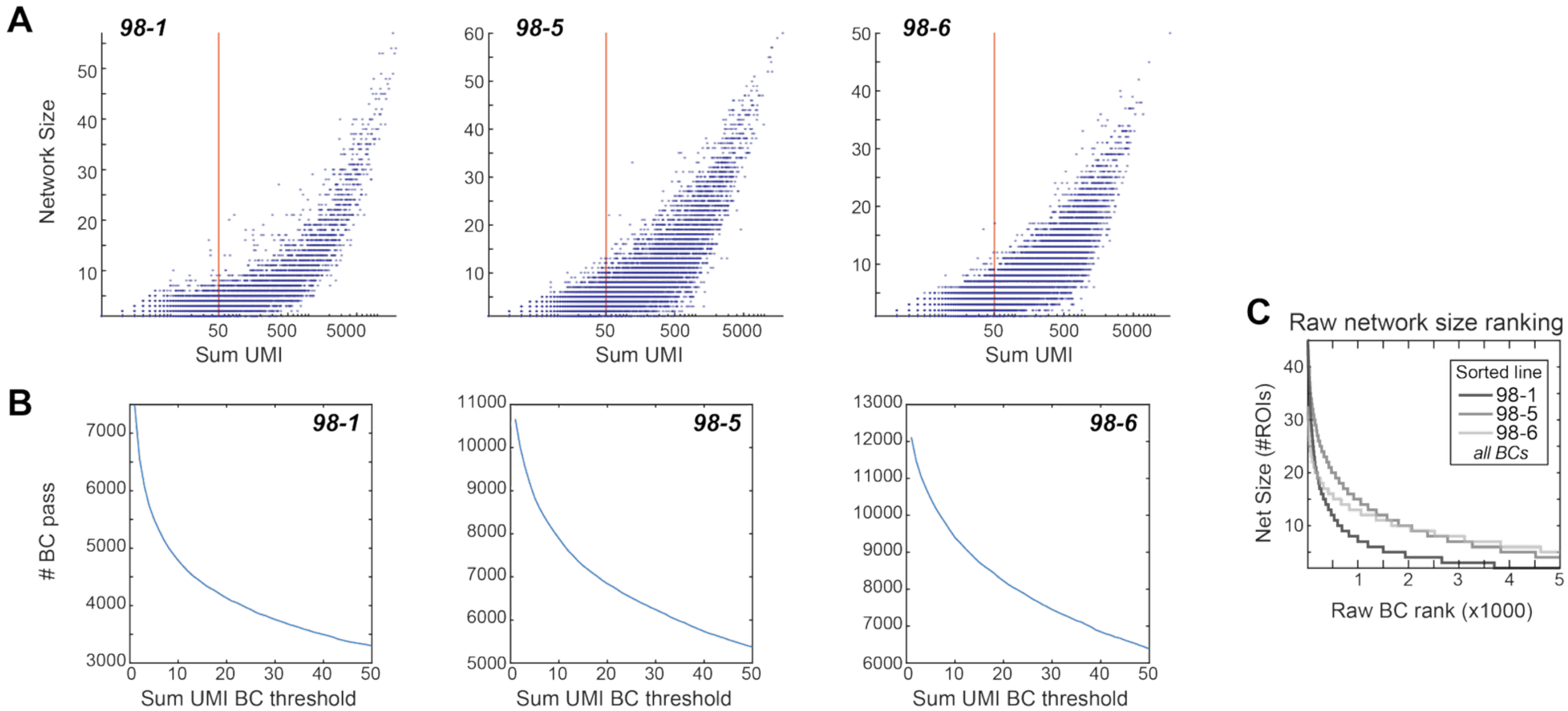

Figure S4. ROInet-seq analysis pipeline: BC detection and threshold evaluation for experiments 98-1, 98-5, 98-6. Related to Figure 4.

(**A**) For all BCs and pre-filtering, total number of UMIs and their potential network size (# of ROIs detected at ≥1UMI).

(**B**) Simulation of the number of BCs included in analysis, by Sum BC thresholds (total detected UMIs) of 1-50UMIs.

(**C**) BC ranking by network size, including all BCs (pre BC-thresholds).

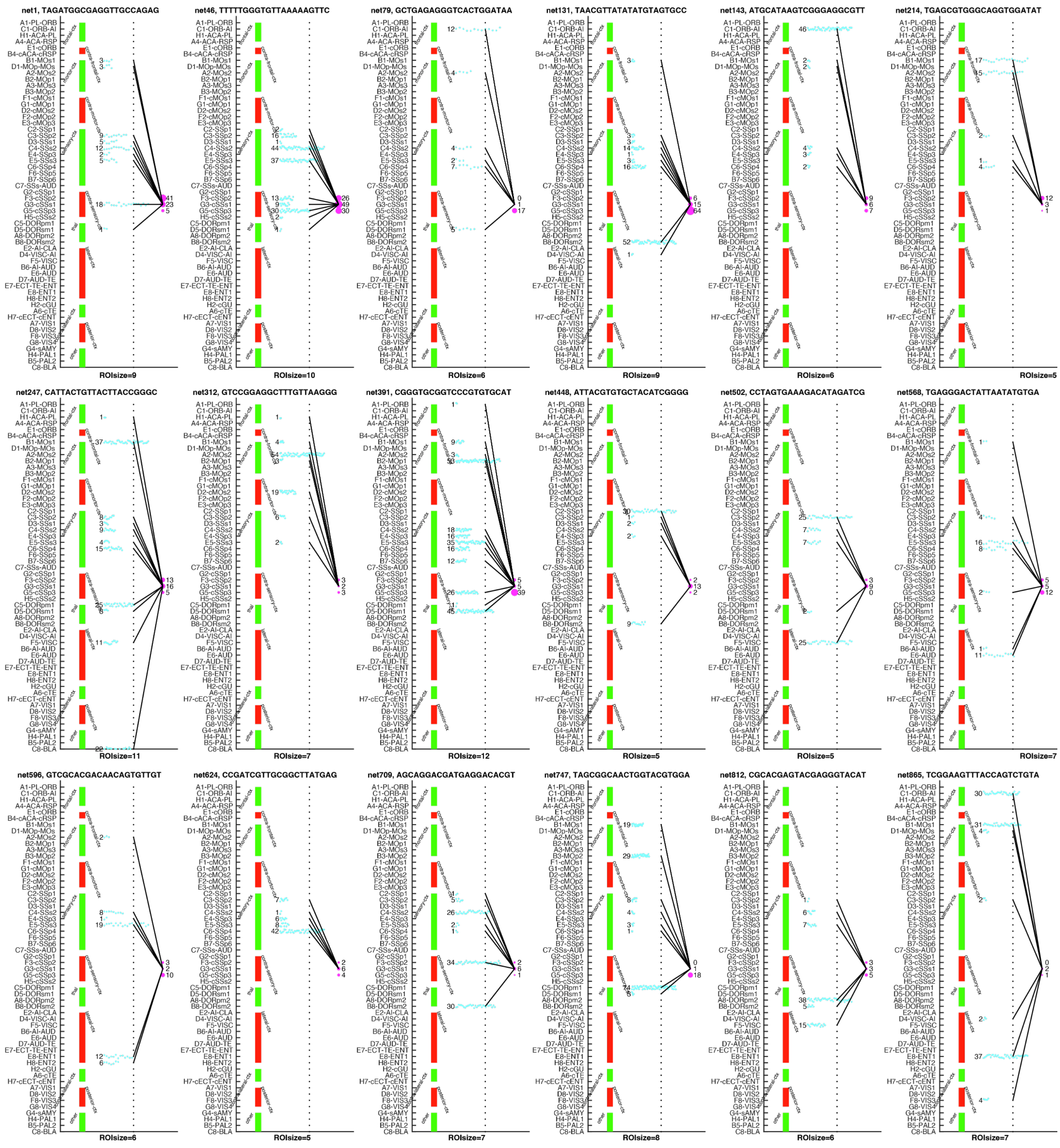

Figure S5. Input networks to single SSp neurons. Related to Figure 5.

Brain-wide input networks to 18 single SSp start site neurons in brain 98-6 at ROI resolution, visualized as dots, where each cyan dot represents detection of 10 unique network BC copies (UMI) in input ROIs, and magenta blob size represents unique BC copies in SSp start site.

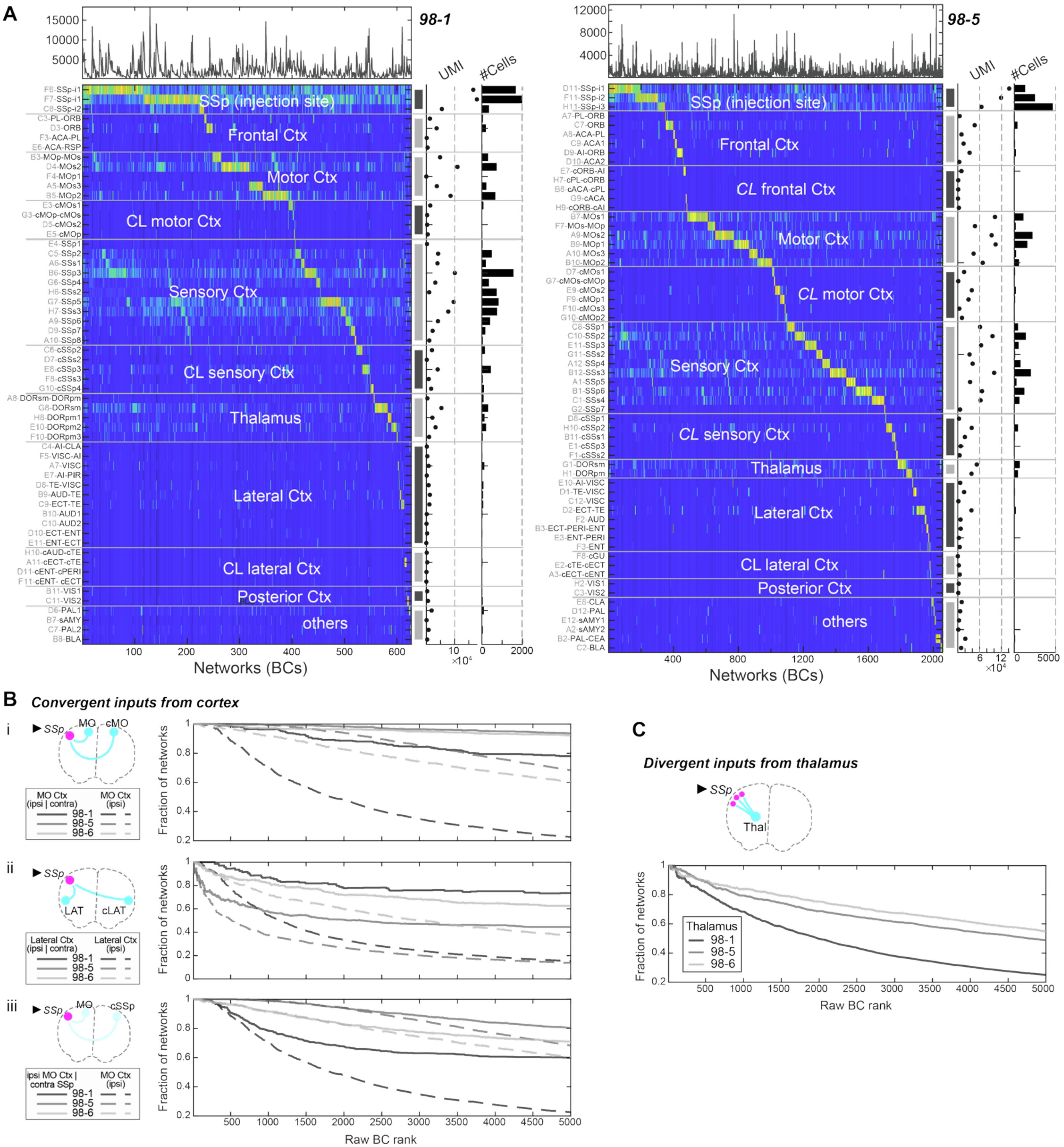

Figure S6. Brain-wide input networks and reproducible motives of convergent and divergent inputs to single SSp start site neurons. Related to Figure 5.

(**A**) Single-cell networks across all ROIs in brains 98-5, 98-6, ordered by dominantly detected ROI. Colormap represents network BC copies (UMI) (blue, low; yellow, high).

(**B-C**) Before thresholds, all BCs were ranked by their detection in #ROIs, from most to least widely spread BC. Lines indicate, per experiment, the fraction of networks that include the indicated ROIs; schematics to the left.

Figure S6, continued.

(**B**) Convergent inputs from ipsi- and contralateral cortical areas. Full lines show ipsilateral inputs conditioned on the simultaneous detection of contralateral inputs; i.e., the fraction of SSp neurons with ipsilateral inputs, given they received a contralateral input: (i) ipsi given contra in the MO cortex, (ii) ipsi given contra in the Lateral cortex, and (iii) ipsi MO cortex given contra SSp cortex. Dashed lines show the fraction of ipsilateral inputs alone; i.e., the fraction expected at chance. For bilateral input convergence within MO and Lat areas ((i) and (ii)), conditional and expected lines quickly separate; i.e., the convergent bilateral network motif is overrepresented (higher than expected). In (iii), in the case of the "unrelated" areas, ipsilateral MO inputs converge with contralateral SSp inputs at a rate as expected by chance (see long overlap of full and dashed lines in 98-5 and 98-6).

(**C**) Fraction of networks that include thalamus, showing that the thalamus maintains a high degree of connectivity to the SSp start site independent of rigorous BC thresholds or exclusion of smaller networks. As discussed in Fig. 3G, thalamic inputs to SSp were considered divergent as the regions had only ~4.5% of fluorescently labeled input cells, while ~57% of BCs were detected in the thalamus (after BC thresholds).

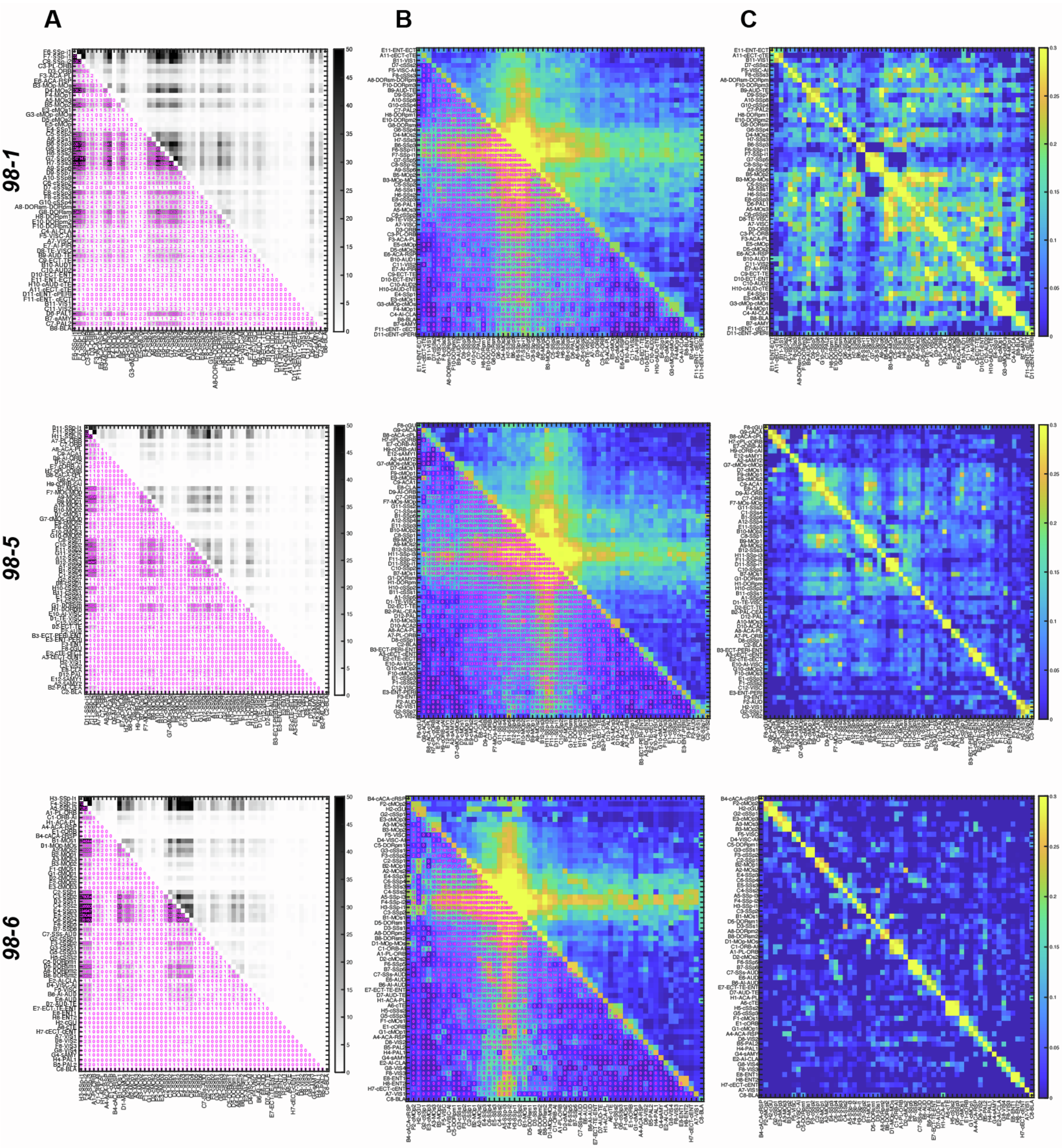

Figure S7. Brain-wide regional overlaps of SSp-cell network. Related to Figure 5.

Convergent regional inputs to single SSp start site neurons in brains 98-1, 98-5 and 98-6. Co-detection of network BCs between individual ROIs;

(**A**) Absolute overlap of all detected network BCs, in ROI order (as Fig. 5, S6);

(**B**) Absolute overlap of all detected network BCs, in order of similarity;

(**C**) As pairwise correlation, in order of similarity (as (**B**)).

Color-/greyscale represents min to max values normalized per ROI (excluding the diagonal).

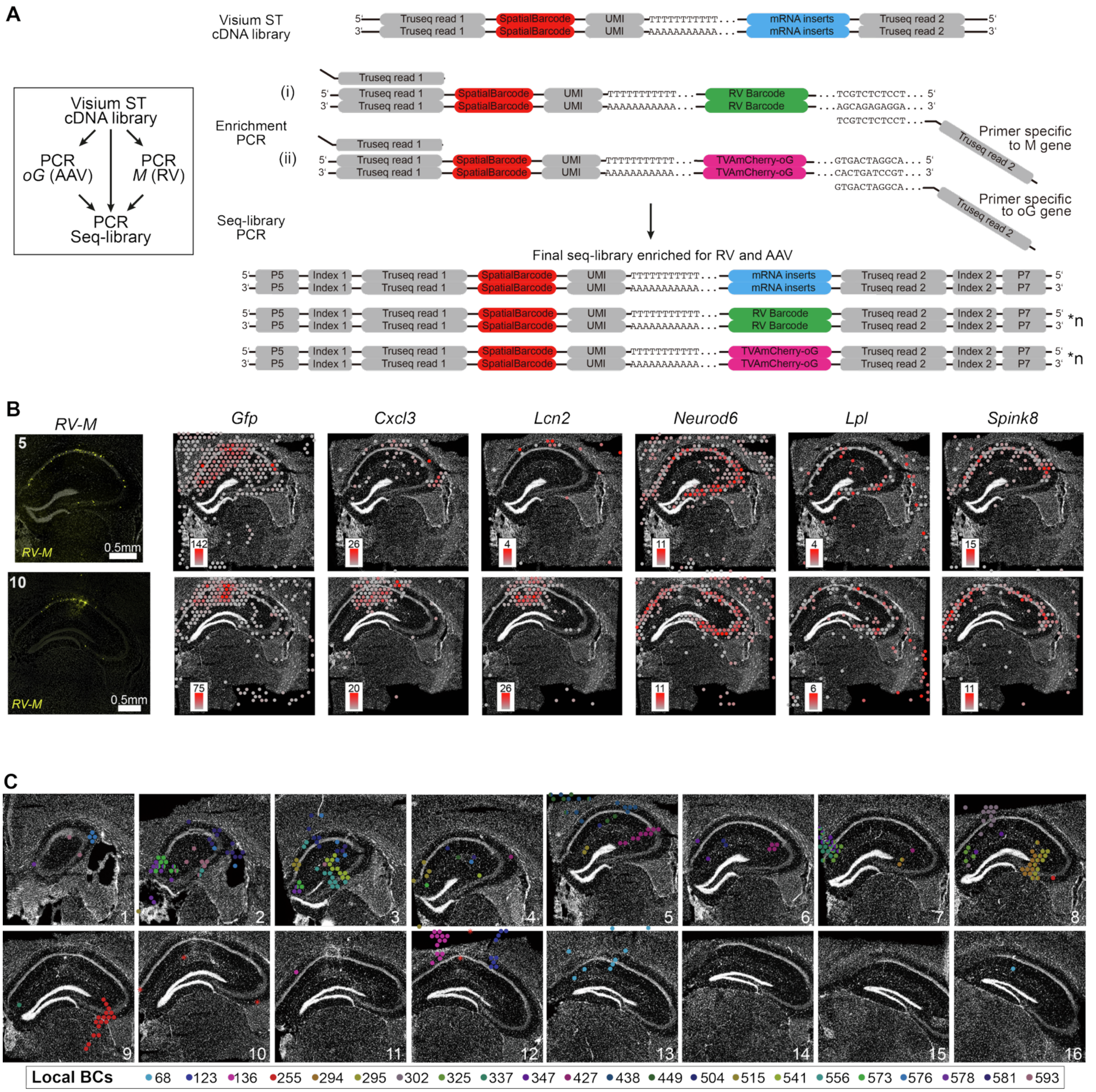

Figure S8. Visium Spatial Transcriptomics (ST) for increased resolution of local network reconstruction. Related to Figure 6.

(**A**) Workflow for amplification of RV (BC) and AAV (TVA) -related transcripts, from full cDNA.

(**B**) Visium ST gene expression of RV (*Gfp*), pyramidal neuron (*Neurod6, Spink8, Lpl*) and inflammatory markers (*Lcn2, Cxcl13*) in sections 5 and 10; with single molecule FISH-validation of RV matrix gene (*M*) expression in consecutive sections shown on the left. Grey, low; red, high; maximum UMI indicated per section. Scale bars, 0.5mm.

(**C**) Spatially resolved detection of 22 abundant, but locally restricted barcodes (blue in Fig. 4E), in 16 a.-p. hippocampus sections of Visium ST; white, nuclei counterstain (DAPI).

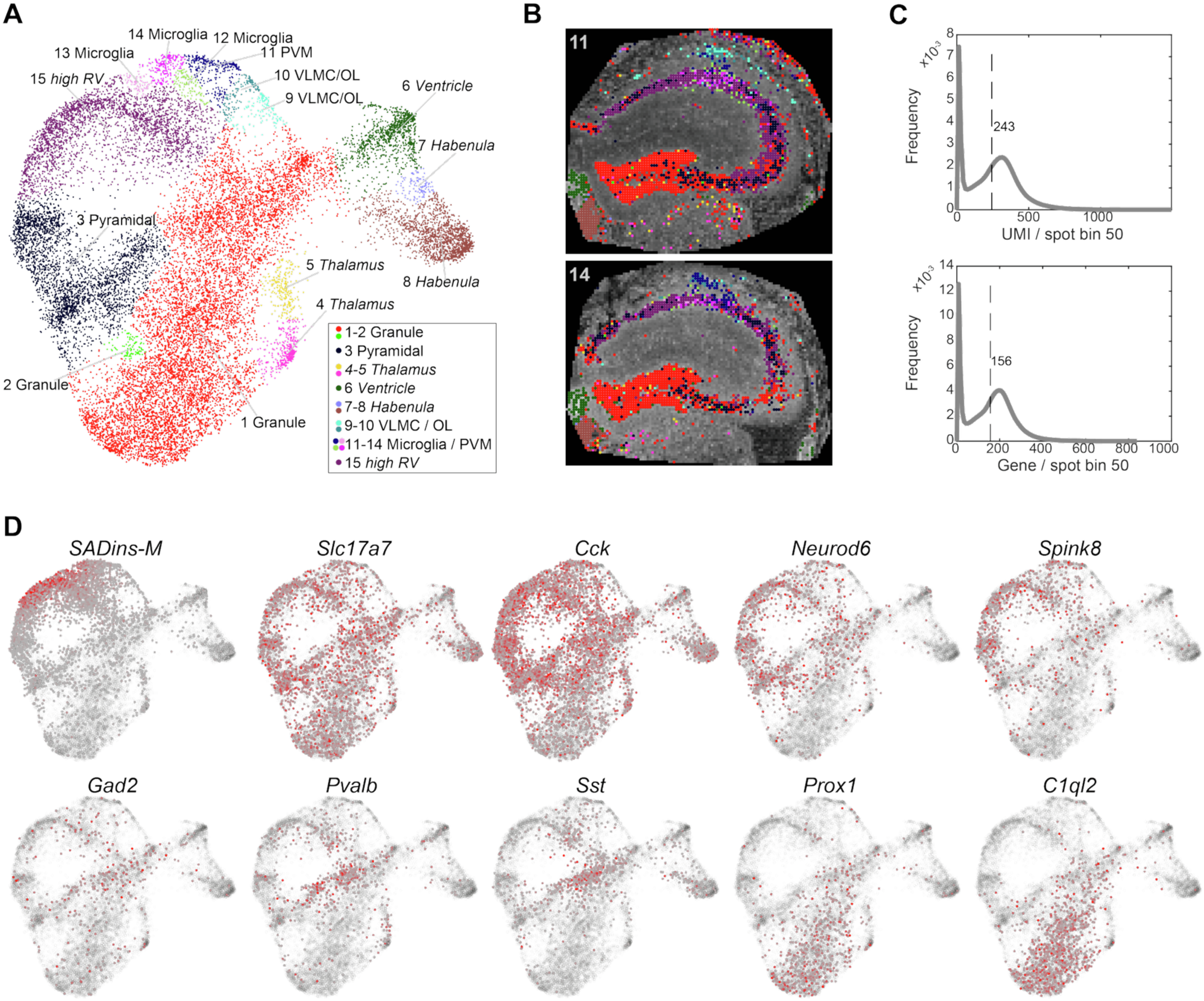

Figure S9. Clustering and gene expression in Stereo-seq STOmics. Related to Figure 7.

(**A**) UMAP clustering of spatial spots, bin 50, colored by molecular type.

(**B**) Molecular types according to UMAP clustering overlayed on sections 11 and 14.

(**C**) UMI and genes detected (bin 50) with median values indicated.

(**D**) Detection of the SAD-B19 RV-transcript M, and excitatory neuron markers (*Slc17a7, Cck*, *Neurod6, Spink8*), GABAergic type markers (*Gad2, Pvalb, Sst*) and granule markers (*Prox1, C1ql2*).

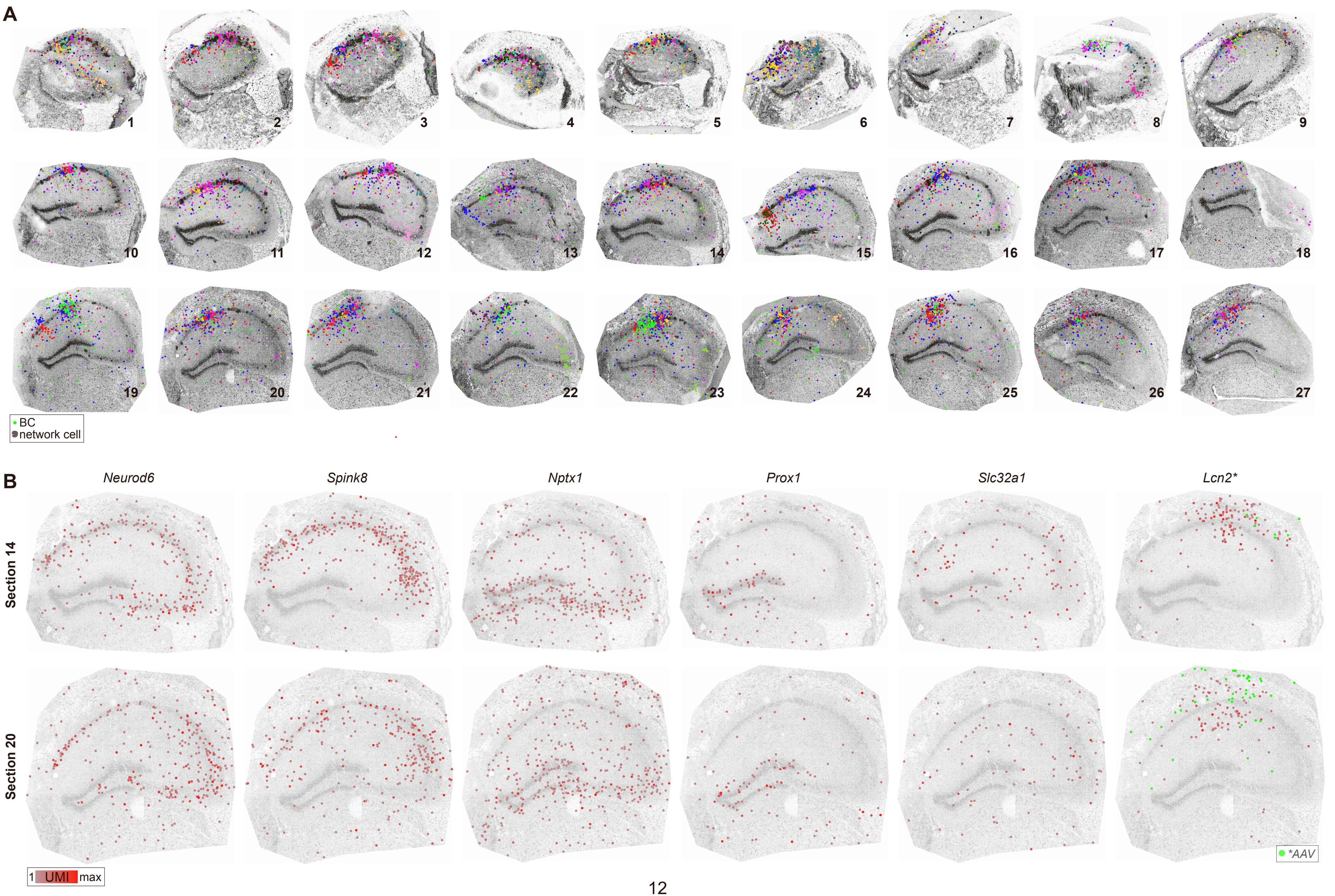
A
1
2
3
4
5
6
7
8
9
10
11
12
13
14
15
16
17
18
19
20
21
22
23
24
25
26
27
BC
network cell
B
Neurod6
Spink8
Nptx1
Prox1
Slc32a1
Lcn2*
Section 14
Section 20
1 UMI max
*AAV

Figure S10. Cell-resolved hippocampus networks on Stereo-seq STOmics, Related to Figure 7.
(**A**) Spatially resolved detection of the top 20 abundant barcodes (colors), in all 27 a.-p. hippocampus sections. Black, network cell outlines. The grey scale showing section anatomy is based on total detected molecules; where dark is the maximum detected molecules.
(**B**) Spatial gene expression of neuronal markers *Neurod6* (pyramidal), *Spink8* (CA1-CA2), *Nptx1* (CA3), *Prox1* (DG), *Slc32a1* (inhibitory) and inflammatory marker *Lcn2*, as well as AAV-transcripts (green) to indicate the injection site, in sections 14 and 20. Maximum spot expression (bin 1) is 4 UMI, except *Slc32a1* section 14 (max=3) and *Nptx1* (max=5 both sections). Grey scale, total detected molecules; where dark is maximum.
